# Long non-coding RNA-derived peptides are immunogenic and drive a potent anti-tumour response

**DOI:** 10.1038/s41467-023-36826-0

**Published:** 2023-02-25

**Authors:** Wojciech Barczak, Simon M. Carr, Geng Liu, Shonagh Munro, Annalisa Nicastri, Lian Ni Lee, Claire Hutchings, Nicola Ternette, Paul Klenerman, Alexander Kanapin, Anastasia Samsonova, Nicholas B. La Thangue

**Affiliations:** 1 Laboratory of Cancer Biology, Department of Oncology, University of Oxford, Old Road Campus Research Building, Oxford, OX3 7DQ UK; 2 Argonaut Therapeutics Ltd, Oxford Science Park, Robert Robinson Avenue, Oxford, OX4 4GA UK; 3 The Jenner Institute, Nuffield Department of Medicine, University of Oxford, Oxford, OX3 7BN UK; 4 Peter Medawar Building for Pathogen Research, University of Oxford, Oxford, OX1 3SY UK; 5 Centre for Computational Biology, Peter the Great Saint Petersburg Polytechnic University, St. Petersburg, 195251 Russia

**Keywords:** Tumour immunology, Tumour-suppressor proteins, CD8-positive T cells, Non-coding RNAs

## Abstract

Protein arginine methyltransferase (PRMT) 5 is over-expressed in a variety of cancers and the master transcription regulator E2F1 is an important methylation target. We have explored the role of PRMT5 and E2F1 in regulating the non-coding genome and report here a striking effect on long non-coding (lnc) RNA gene expression. Moreover, many MHC class I protein-associated peptides were derived from small open reading frames in the lncRNA genes. Pharmacological inhibition of PRMT5 or adjusting E2F1 levels qualitatively altered the repertoire of lncRNA-derived peptide antigens displayed by tumour cells. When presented to the immune system as either ex vivo-loaded dendritic cells or expressed from a viral vector, lncRNA-derived peptides drove a potent antigen-specific CD8 T lymphocyte response, which translated into a significant delay in tumour growth. Thus, lncRNA genes encode immunogenic peptides that can be deployed as a cancer vaccine.

## Abstract (web summary)

Long noncoding RNA molecules are RNA transcripts long thought to remain untranslated. In this study, the authors demonstrate that certain lncRNA can be translated into peptides that are immunogenic to CD8^+^ T cells and promote anti-tumour responses when delivered as vaccine vectors in mice.

## Introduction

Most of the human genome consists of non-classical genes, including, for example, genes encoding microRNA and long non-coding (lnc) RNA molecules^1,2^. LncRNA genes are a major source of transcription in mammalian cells, typically encoding transcripts with lengths of over 200 nucleotides, most of which are believed to exist as untranslated RNAs^2^. A relatively small number of lncRNA transcripts have been shown to be processed in the same way as mRNA, and in rare cases suggested to perform biological roles^2^. Although a cancer connection has been established for certain lncRNAs (for example, *MALAT1* as a prognostic marker for patient survival in colorectal cancer^3–5^), it remains unclear what role, if any, the majority of lncRNAs serve in malignant disease.

The retinoblastoma protein (pRb)-E2F pathway is a key point of control in the cell cycle and is often under aberrant control due to oncogenic mutation in human tumours, and deregulation of the pathway is widely regarded as a ‘hallmark’ of cancer^6^. Classically, the pRb tumour suppressor protein is viewed as a negative regulator of E2F transcription factors, where E2F acts as a transcriptional hub through which pRb exerts its cellular effects. However, it has become apparent that the pRb-E2F pathway regulates a much broader gene network than originally envisaged^7–9^. The extended target gene repertoire is regulated in part by PRMT5, which catalyses an influential residue-specific methylation event in a central arginine (R)-rich cluster. This modification affects the biological properties of E2F1^7,8^ and switches E2F1 from its primary role as a transcriptional regulator to one with a wider effect on the regulation of gene expression, including alternative RNA splicing^9^. The frequent over-expression of PRMT5 in diverse human tumours and the critical role that E2F plays in the cancer cell cycle^10^ argues strongly for the importance of the interplay between PRMT5 and E2F1 in malignant disease.

Here, we describe a group of lncRNA genes that are translated and further processed into small antigenic peptides presented on MHC class I protein complexes. Both PRMT5 and E2F1 regulated the expression of lncRNA genes and therefore impacted on the repertoire of peptides presented to the immune system by cancer cells. A stand-alone therapeutic vaccine composed of lncRNA-derived peptide antigens was found to be immunogenic and drove a CD8 T lymphocyte response that resulted in a significant delay in tumour growth. Our results identify the lncRNA non-coding genome as an unexpected source of immunogenic tumour antigens which can be engineered into a cancer vaccine to facilitate effective antitumour immunity.

## Results

### E2F1 and PRMT5 control lncRNA expression in murine tumours

**Fig. 1:**
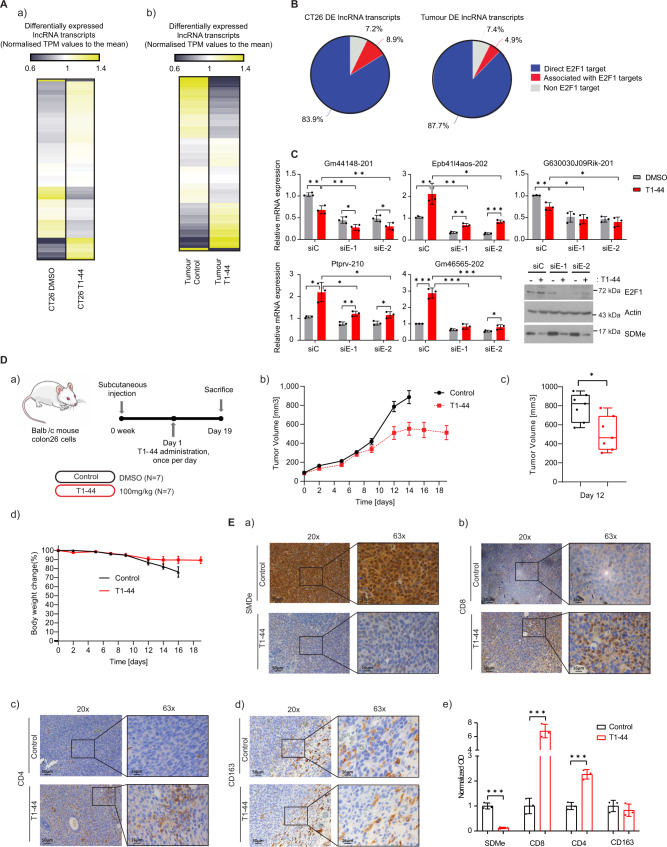
Differential expression analysis of lncRNA transcripts in CT26 cells and mouse tumours. **A** (a) Differential expression changes in lncRNA transcripts in T1-44 treated CT26 (*q* < 0.05) cells with respect to DMSO treatment. Transcript Per Million (TPM) expression values were normalised to the mean. Yellow, up-regulation; blue, down-regulation; ivory, minimal change. Expression data was derived from three independent experiments (each with one technical sample). (b) As above, but displaying differential expression in lncRNA transcripts in T1-44-treated colon26 tumours (*p* < 0.05) with respect to control. Expression data were derived from tumours collected from three mice (from one experiment). **B** Percentage of lncRNA genes differentially regulated at a statistically significant level in CT26 cells (left) or Colon26 tumours (right) that score as potential direct E2F1 target genes, or are associated with other potential E2F1 target genes. **C** RT-qPCR analysis of CT26 cells transfected with E2F1 siRNAs and treated with 1 µM T1-44 (indicated lncRNA transcripts are labelled with their ENSEMBL transcript name). An immunoblot to display input protein levels is included. SDMe was used as a marker for T1-44 activity; *n* = 3 independent experiments (each with three technical replicates). Results are mean values ± SD; one-way ANOVA with Tukey’s multiple comparisons test; * adjusted *P* value < 0.05, ** adjusted *P* value < 0.01, *** adjusted *P* value <0.001. **D** (a) Colon26-bearing BALB/c mice received T1-44 orally at 100 mg/kg for 19 days with respect to vehicle only control; *n* = 7 mice per group; experiment was performed twice; (b) Absolute tumour volume presented as a mean ± SEM, *n* = 7. (c) Absolute tumour volume of individual mice at day 12 (two-tailed Student’s *t* test; * *p*  =  0.0143), *n* = 7; box and whiskers are defined as minimum, first quartile, median, third quartile, and maximum of data. (d) Relative body weight presented as a mean value ± SEM, *n* = 7. **E** Immunohistochemical staining of SDMe (a), anti-CD8 (b), anti-CD4 (c), or anti-CD163 (d) in Colon26 tumours collected at 19 days post treatment with T1-44, or at day 14 from non-treated controls. Original magnification: 20×, scale bar, 50 μm; and 63×; scale bar, 16 μm. *n* = 4 mice from Fig. 1D; (e) Optical density is presented as mean ± SD; two-tailed Student’s *t* test; *n* = 4 independent experiments (each performed on two separate slides); *** adjusted *P* value <0.001.

We reasoned that the interplay between PRMT5 and E2F1 may influence the non-coding genome and focused our attention on atypical non-coding RNA genes. We therefore examined the effect of pharmacological inhibition of PRMT5 activity using a small molecule active site inhibitor, T1-44, which is an effective and selective inhibitor of PRMT5 (supplementary Fig. 1A,^11^) in murine CT26 colorectal cancer (CRC) cells. We performed a genome-wide RNAseq analysis on the T1-44 treated cells, which was compared to the control RNAseq. We mined the RNAseq datasets to evaluate the effect of PRMT5 on lncRNA transcripts. A set of lncRNAs was identified to be significantly regulated (q < 0.05) with 109 up-regulated and 282 down-regulated upon PRMT5 inhibition relative to the control treatment (Fig. 1A, supplementary Fig. 1B and supplementary Data 2). Remarkably, 83.9% of these differentially expressed lncRNAs were derived from genes that contained E2F1 binding site ChIP-seq peaks with close proximity to their transcription start site (TSS), whilst an additional 8.9% of the lncRNA genes were at the genomic level located close to or within another predicted E2F target gene (Fig. 1B). We chose a small group of the lncRNA genes that were hypothetical targets for E2F1 (from the ChIP-seq analysis) for further analysis. We observed a significant effect on lncRNA expression in T1-44 treated cells compared with untreated cells, with *Gm46565*, *Ptprv* and *Epb41l4aos* showing increased expression, and *Gm44148* and *G630030J09Rik* reduced expression (Fig. 1C). We also evaluated lncRNA expression in siE2F1-treated cells (using two independent siRNAs), where lncRNA expression level was generally down-regulated across the lncRNA genes examined (Fig. 1C). These results show that murine lncRNA genes exist that are susceptible to control by PRMT5 and E2F1.

We followed on to investigate whether lncRNA genes were under a similar level of control in mouse tumours. For this study, we used the syngeneic colon26 tumour model growing in vivo and assessed the impact of T1-44 treatment. Treating tumour bearing mice with T1-44 caused a significant delay in tumour growth (Fig. 1D and supplementary Fig. 1C). RNAseq performed on untreated compared to T1-44 treated tumours identified a significant set of lncRNA transcripts that were differentially regulated between the two treatments (*p* adj < 0.05; Fig. 1A, supplementary Fig. 1B and supplementary Data 2). By comparing with annotated ChIP-seq data sets, ~88% of the differentially regulated lncRNA genes were potential E2F1 targets (Fig. 1B). We took a small subset of lncRNAs from the differentially regulated set and analysed their expression using qPCR. Whereas *4930473A02Rik*, *Gm45441*, *Gm15156*, *Lncppara*, *Kcnmb4os1*, *Lncenc1* and *Epb41l4aos* transcripts were up-regulated, *Gm36445* was down-regulated following treatment with T1-44 (supplementary Fig. 1D). Moreover, some of the lncRNAs identified in the CT26 cell RNAseq and characterised at the single gene level, including *Epb41l4aos*, *Gm44148* and *Gm46565*, exhibited a similar expression pattern in CT26 cells in vitro and colon26 tumours in vivo (supplementary Fig. 1E compared to Fig. 1C). These results suggest therefore that PRMT5 and E2F1 regulate lncRNA gene expression in mouse tumours in a similar way to that seen in the murine cancer cell line.

### PRMT5 regulates the immune response in the tumour micro-environment

The inhibition of tumour growth upon T1-44 treatment coincided with a reduced level of the PRMT5 symmetric-dimethyl (SDMe) mark within colon26 tumour biopsies (Fig. 1E), thus confirming catalytic inhibition of PRMT5 in treated mice. Upon further examination, we found that T1-44 treatment had a striking impact on the infiltrating T lymphocyte population in the tumour micro-environment (TME), most clearly evidenced by the influx of cytotoxic CD8 and a modest increase in helper CD4 T lymphocytes (Fig. 1E); on other relevant cell populations, like tumour-associated macrophages (detected by anti-CD163 immuno-staining), the effect of T1-44 treatment was minimal (Fig. 1E). Furthermore, IL-6 levels were elevated in serum from T1-44 treated mice (supplementary Fig. S1F). We considered that the increased level of CD8 T lymphocytes was due to an effect on the adaptive immune response, and because CD8 T lymphocytes principally engage with the MHC class I antigen complex through their T cell receptor, it was plausible that T1-44 treatment altered antigen presentation via the MHC class I protein complex.

**Fig. 2:**
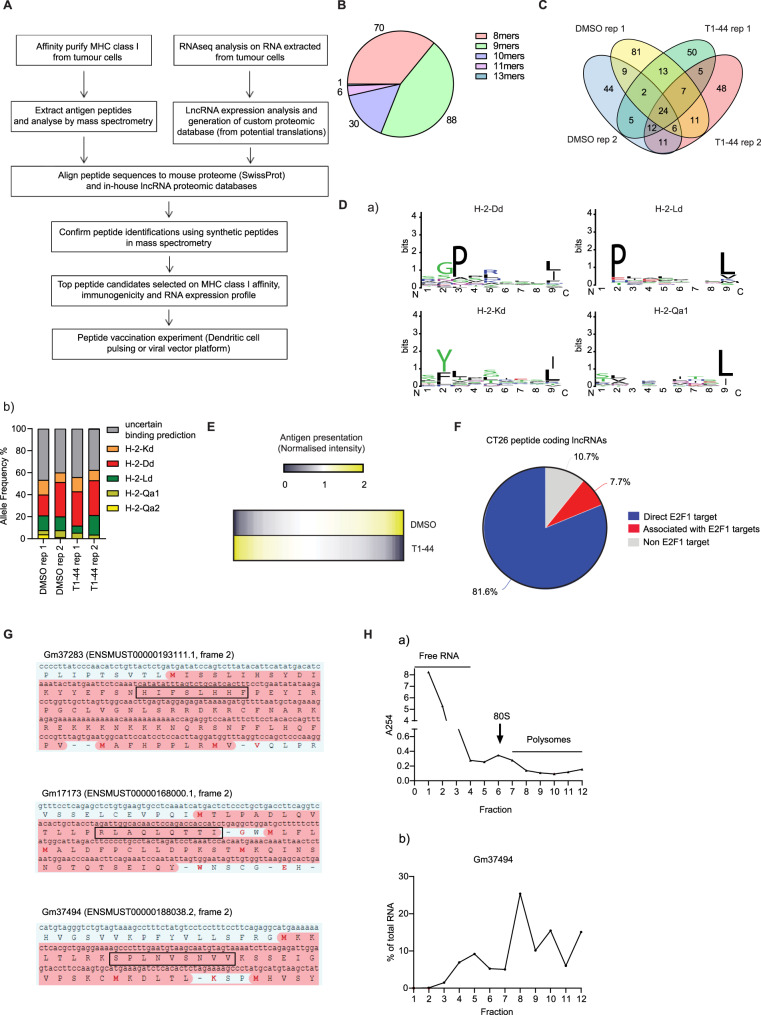
Immunopeptidomics analysis of CT26 cells. **A** Workflow of the immunopeptidomics platform. **B** Peptide length of each murine lncRNA-derived peptide is displayed as pie chart. 195 quantifiable peptides in total (pooled from two independent experiments) were detected (data derived from an immunopeptidomics experiment performed in biological independent replicate. **C** Immunopeptidomics analysis in T1-44 treated (1 µM for 72 h) or DMSO treated CT26 cells. The experiment was performed in biological replicate (rep1, rep2). Indicated is the overlap of MHC-bound lncRNA-derived peptides identified from the qualitative immunopeptidomics analysis. 328 peptides were detected. **D** (a) Sequence logos of amino acid conservation in lncRNA-derived peptides for each of the indicated MHC alleles; (b) Predicted MHC allele frequency for identified lncRNA-derived peptides are displayed, represented as a percentage of total. **E** Heatmap of lncRNA derived peptide abundance from the quantitative immunopeptidomics analysis (195 peptides) (both up- and down-regulated in T1-44 treatment with respect to DMSO). Relative abundance values were converted by normalisation to the mean. Yellow colour represents up-regulation, whilst blue colour represents down-regulation. Ivory colour represents no change in abundance. *n* = 2 independent experiments (each with two technical replicates); **F** The percentage of lncRNA genes giving rise to MHC class I bound peptides that score as potential direct E2F1 target genes or are associated with other potential E2F1 target genes. The analysis was performed on all unique peptide coding lncRNA genes identified in our immunopeptidomics analyses. **G** Part of the *Gm37283*, *Gm17173*, and *Gm37494* lncRNA transcripts are displayed, with the predicted ORF (shown in red) giving rise to the identified MHC class I bound peptide (boxed in black). Potential start methionine residues are highlighted in red text. **H** (a) Example polysome profiling assay from CT26 cells, indicating total RNA quantity detected in each collected fraction (by absorbance reading at 254 nm). (b) Polysome profiling assay for *Gm37494* is displayed. Data are presented as percentage of total RNA in each fraction; *n* = 3 independent experiments (each with three technical replicates).

To address whether the peptide antigen content of the MHC class I complex was altered upon treating cancer cells with compound T1-44, we performed a mass spectrometry (MS) immunopeptidomics analysis to assess the repertoire of peptides displayed by the MHC class I complex in treated relative to untreated cells. The results revealed a large group of MHC class I bound peptides (Supplementary Data 3 and 4). Given the presence of open reading frames (ORFs) in many lncRNA genes^12^, we were interested to examine whether lncRNAs were capable of encoding peptides which contributed to the MHC class I peptide repertoire. To this end, we first generated an in-house proteomic database containing predicted translations from all 3-frames of every lncRNA transcript expressed at detectable levels in our CT26 RNA-seq dataset, which included all theoretical ORFs. Peptides identified in the immunopeptidomics analysis were then matched to either this database or a standard proteomic database (containing all reviewed mouse SwissProt protein entries; Fig. 2A). Interestingly, we identified 382 unique peptides derived from lncRNA genes (with a mean size of 9 residues; Fig. 2B, C), representing 6.5% of the total peptides detected in the immunopeptidomics analysis (Fig. 2C and supplementary Data 3 and 4).

### LncRNAs encode MHC class I bound peptides

The lncRNA-derived peptides had predicted high affinity for the murine MHC class I alleles H-2-K^d^, D^d^, L^d^, Qa1, Qa2 (Fig. 2D) and exhibited the conserved residues required for efficient MHC class I binding (Fig. 2D), closely resembling the characteristics of peptide sequences derived from protein-coding genes^13^. To validate the peptide output from the analysis, a selection of the peptides identified in the immunopeptidomics analysis was subsequently confirmed using mass spectrometry to compare to the synthetic peptide sequence, where complete identity was established (supplementary Fig. 3A). Most importantly, the lncRNA peptides exhibited qualitative and quantitative differences between T1-44 treated and untreated cells (presented as heat map in Fig. 2E, supplementary Data 3 and 4).

We then examined the expression properties of lncRNA genes that encoded MHC class I bound peptides. We found that many of the lncRNAs were up-regulated in CT26 cells grown in vitro (supplementary Fig. 2A) and in tumours upon T1-44 treatment (supplementary Fig. 2B), with a smaller group down-regulated. Moreover, the expression profile of lncRNAs in CT26 cells typically reflected a similar relative change in the derived peptide measured by immunopeptidomics, such as *Gm37283* (encoding peptide sequence HIFSLHHF) and *Gm17173* (encoding peptide sequence RLAQLQTTI) which were up-regulated, and *4732463B04Rik* (encoding peptide sequence RGPLLEKLF) which was down-regulated upon T1-44 treatment (highlighted in supplementary Fig. 2A and supplementary Data 4). Further, the majority of MHC class I bound peptides were derived from lncRNA genes that score as E2F targets (by reference to ChIP-seq data sets); namely, around 81% were direct E2F1 targets, with a further 8% associated with or over-lapping known E2F1 target genes (Fig. 2F). A small number of these lncRNAs were evaluated for the role of E2F1 using siE2F1 silencing; most of the lncRNAs tested showed an E2F1-dependency, together with an impact of T1-44 treatment (supplementary Fig. 2C).

MHC class I-associated peptides are usually generated from larger proteins that are subject to proteolytic degradation and funnelled into the endo-lysosomal vesicular system^14^. Because we identified many peptides derived from lncRNA genes, widely regarded as non-coding^12^, we wished to test whether the lncRNAs encoded larger proteins that could, theoretically, be processed to generate a small peptide. For many of the lncRNAs that encode a MHC class I bound peptide, we identified an ORF in the gene sequence (examples shown in Fig. 2G). Notably, most of the lncRNA ORFs were small, encoding polypeptides with less than 100 residues (supplementary Fig. 3B). Further, transcripts derived from the lncRNA genes were able to associate with the translating polysomal fraction of ribosomes (for example *Gm 37494* in Fig. 2H; *Gm37283*, *Gm17173*, *Gm47761*, *Gm29253*, *Gm42047* and *Gm20939* in supplementary Fig. 3C), an observation consistent with a conventional translation mechanism. However, we wanted to directly test this idea and therefore cloned the predicted ORF cDNA, together with its upstream sequences where the ribosome binding site would be located, into an expression vector tagged with the FLAG epitope at the C-terminal end (supplementary Fig. 3D). As an example, the *Gm29253* lncRNA had an ORF encoding a theoretical polypeptide of 26kD (supplementary Fig. 3D). In transfected cells, a specific polypeptide derived from ectopic expression of the *Gm29253* ORF was detected by immuno-staining and immunoblotting, with the anticipated molecular weight for the predicted ORF (supplementary Fig. 3D). We therefore conclude that lncRNAs that give rise to MHC class I bound peptides can associate with ribosomes and be translated into polypeptides which, then, are likely processed to generate peptides that associate with the MHC class I protein complex.

### E2F1 and PRMT5 control expression of the human lncRNA genes

**Fig. 3:**
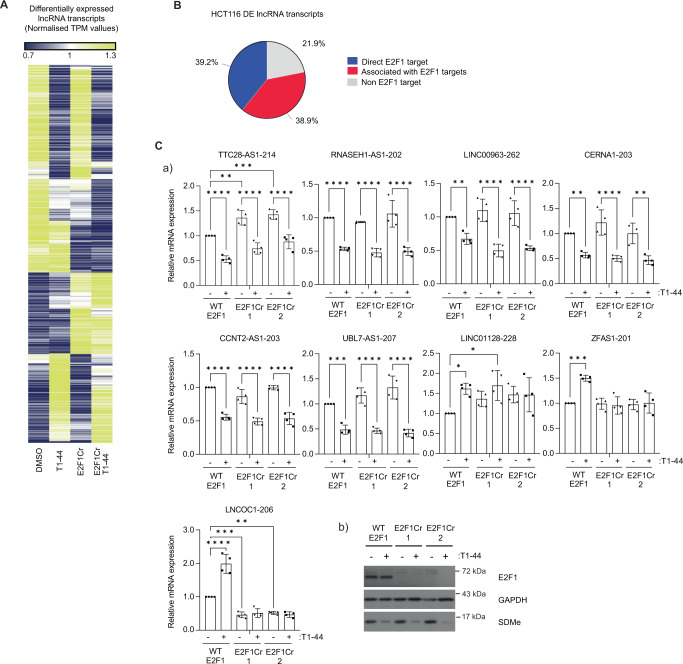
Differential expression analysis of lncRNA transcripts present in HCT116 cells. **A** Differential expression changes in lncRNA transcripts (*q* < 0.05) in WT E2F1 and E2F1Cr cells treated with T1-44 for 48 h, with respect to WT E2F1 DMSO treatment. Transcript Per Million (TPM) expression values were converted by normalisation to the mean. Yellow colour represents up-regulation, whilst blue colour represents down-regulation. Ivory colour represents minimal change. Expression data were derived from three independent experiments. **B** Percentage of lncRNA genes differentially regulated at a statistically significant level (*q* < 0.05) in HCT116 cells that score as potential direct E2F1 target genes or are associated with other potential E2F1 target genes. **C** (a) WT E2F1 and two separate E2F1 Cr cell line clones were treated with 1 µM T1-44 for 48 h prior to RT-qPCR analysis to determine the expression of the indicated lncRNA transcripts (labelled with their ENSEMBL transcript name). *n* = 4 biologically independent experiments (each performed in technical triplicate), results presented as mean values ± SD; one-way ANOVA with Tukey’s multiple comparisons tests and * adjusted *P* value <0.05, ** adjusted *P* value <0.01, *** adjusted *P* value <0.001, **** adjusted *P* value <0.0001; (b) An immunoblot to display input protein levels. SDMe was used as a marker for T1-44 activity.

To examine whether human lncRNA genes behave in a similar way to that observed in murine cancer cells and further investigate the effect of PRMT5 and E2F1, we explored lncRNA expression upon PRMT5 inhibition and CRISPR knock-out (KO) of the *E2F1* gene in HCT116 cells derived from human CRC^11^. RNA-seq datasets derived from these cell lines and treatment conditions were mined for significant changes in lncRNA gene expression (q < 0.05) which revealed transcripts that were differentially expressed between each condition (Fig. 3A, supplementary Fig. 4A and supplementary Data 1); differentially-expressed lncRNA transcripts dependent on PRMT5, E2F1 and PRMT5/E2F1 together were evident (Fig. 3A). Furthermore, lncRNA transcripts were either up- or down-regulated (with 237 up- compared to 303 down-regulated), with some overlap between the conditions (Fig. 3A, supplementary Fig. 4A). By inspecting annotated E2F1 ChIP-seq data sets^15^, we found that many of the lncRNA transcripts that scored as differentially expressed upon manipulating PRMT5 and/or E2F1 were derived from genes that had E2F1 ChIP-seq reads in close proximity to the TSS or within the body of the transcribed sequence (examples shown in supplementary Fig. 5). Thus, 39.2% of the differentially expressed lncRNAs were derived from genes with recognisable E2F binding sites in the promoter region, whilst another 38.9% were located close to or within a predicted E2F target gene (Fig. 3B); this contrasted with 13.5% of non-regulated lncRNA genes exhibiting E2F binding sites (supplementary Fig. 4B).

We validated the role of PRMT5 and E2F1 by choosing a small number of lncRNA genes for detailed expression analysis. Upon PRMT5 inhibition, expression patterns were apparent that decreased like *TTC28-AS1*, *RNASEH1-AS1*, *LINC00963*, *CERNA1*, *CCNT2-AS1* and *UBL7-AS1*, or increased like *LINC01128*, *ZFAS1* and *LNCOC1* (Fig. 3C). When lncRNA expression was compared between WT and KO E2F1 cells, *LNCOC1* expression decreased whereas *TTC28-AS1* was at higher levels (Fig. 3C). In some cases, the effect of T1-44 on lncRNA expression was dependent on the presence of WT E2F1, as there was no or reduced impact in E2F1 KO cells (see *LNCOC1*, *LINC01128* and *ZFAS1*) (Fig. 3C). We also evaluated a number of other established PRMT5 inhibitors, including JNJ-64619178 and LLY-283^16,17^, where the observed effects on lncRNA expression were the same as treatment with T1-44 (Supplementary Fig. 4C).

We further assessed whether the speculative binding sites identified in the ChIP-seq data sets were real binding sites in HCT116 cells by designing primers around the E2F binding sites and performing gene-specific ChIPs. E2F1 was detected in the chromatin of the lncRNA genes that we tested (examples shown for *UBL7-AS1*, *CERNA1*, *CCNT2-AS1*, *LINC00963*, *RNASEH1-AS1*, *TTC28-AS1*, *ZFAS1*) in E2F1 expressing HCT116 cells, in contrast to the E2F1 KO cells where no E2F1 enrichment was evident (Supplementary Fig. 4D). We conclude therefore that a large set of lncRNA genes exist where PRMT5 and E2F1 play a significant role in regulating their expression.

### LncRNA-derived MHC class I associated peptides in human tumour cells

**Fig. 4:**
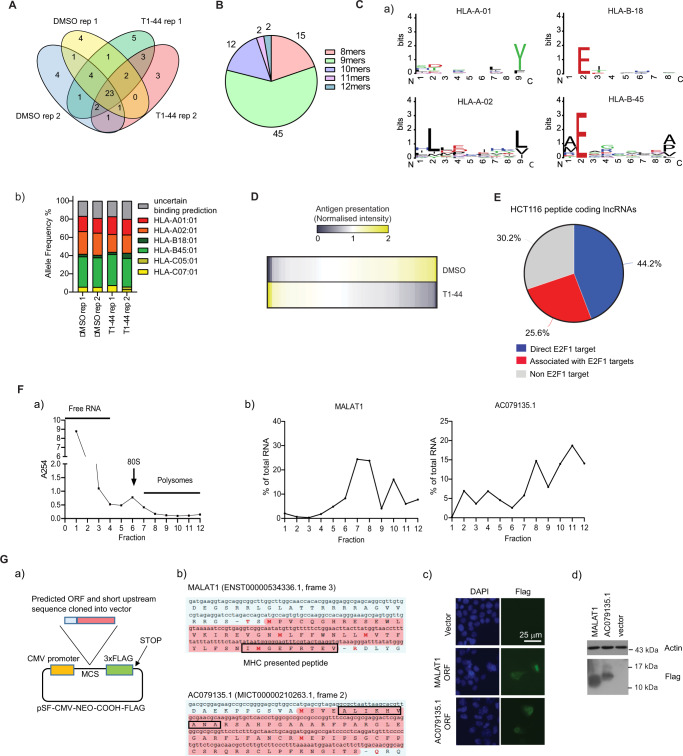
Immunopeptidomic analysis of HCT116 cells. **A** Immunopeptidomics analysis in T1-44 (1 µM for 48 h) or DMSO-treated HCT116 cells. The experiment was performed in biological duplicate. Indicated is the overlap of MHC-bound lncRNA-derived peptides identified from the qualitative analysis. 55 peptides were identified. **B** Peptide length of each human lncRNA-derived peptide is displayed as pie chart. 76 quantifiable peptides in total (pooled from two independent experiments) were detected (data derived from an immunopeptidomics experiment performed in biological independent replicate, each with 2 technical replicates. **C** (a) Sequence logos of amino acid conservation in lncRNA-derived peptides for each MHC allele; b) Predicted MHC allele frequency for identified lncRNA-derived peptides is displayed as a percentage of total. **D** Heatmap of lncRNA-derived peptide abundance from the quantitative immunopeptidomics analysis (76 peptides) (both up- and down-regulated in T1-44 treatment vs. DMSO). Relative abundance values were converted by normalisation to the mean. Yellow, up-regulation; blue, down-regulation; ivory, no change in abundance. *n* = 2 independent experiments (each with two technical replicates); **E** The percentage of lncRNA genes giving rise to MHC class I-bound peptides that score as potential direct E2F1 target genes, or are associated with other potential E2F1 target genes. Analysis was performed on all unique peptide-coding lncRNA genes identified. **F** (a) Example polysome profiling assay from HCT116 cells, indicating total RNA quantity detected in each collected fraction. (b) Polysome profiling assays for *MALAT1* and *AC079135.1* lncRNAs are displayed. Data are presented as percentage of total RNA in each fraction; *n* = 3 independent experiments (each with three technical replicates); **G** (a) Diagram of lncRNA ORF cloning strategy. The predicted ORF and potential endogenous ribosome binding site were inserted in frame with a 3xFLAG tag. A ribosome binding site is not provided in the vector itself. (b) Part of the *MALAT1* and *AC079135.1* lncRNA transcripts are displayed, with the predicted ORF (shown in red) giving rise to the identified MHC class I-bound peptide (boxed in black). Potential start residues are highlighted in red text. (c) Immune-fluorescence of HCT116 cells transfected with *MALAT1* and *AC079135.1* ORF-Flag plasmids. *n* = 2 independent experiments (d) Transfected HCT116 cells analysed by immunoblot. *n* = 3 independent experiments.

We performed another immunopeptidomics analysis on MHC class I associated peptides in human HCT116 cells, comparing untreated with T1-44 treated cells, which also identified a significant number of peptides derived from human lncRNA genes (118 unique peptides in total; Fig. 4A, Supplementary Fig. 6A and Supplementary Data 5–7). Individual lncRNA-derived peptide sequences were confirmed by comparing the mass spectrometry immunopeptidomics peptide spectrum to its synthetic peptide counterpart (Supplementary Fig. 6C). The size of the peptides was on average 9 residues (Fig. 4B) with the conserved residues apparent that are required for human HLA MHC class I binding (Fig. 4C)^13^ and were predicted to have high affinity for the human HLA-A, -B, and -C MHC class 1 proteins (Fig. 4C). Furthermore, quantitative analysis of the peptides (from the immunopeptidomics analysis) indicated that 10% of the peptides were up-regulated, 32% down-regulated and 58% unchanged upon T1-44 treatment (Fig. 4D and Supplementary Fig. 6B).

We measured the expression of some of the human lncRNA genes that encode the MHC class I bound peptides by qPCR and found that upon T1-44 treatment many were differentially expressed (Supplementary Fig. 7A), in some cases exhibiting a similar relative expression change to that seen for the peptide. For example, the increased level of peptides derived from *HELLPAR* (peptide sequence LSLSLSLQFS) and *RP11-660L16.2* (peptide sequence RLATHIDGA) lncRNAs reflected increased lncRNA expression under T1-44 treatment, whilst a number displaying reduced expression including *AC079135.1* (peptide sequence AEKPPGSVA), *RP11-319G6.1* (peptide sequence EETYFHLF) and *VPS9D1-AS1* (peptide sequence RLLQETHQA) lncRNAs coincided with reduced levels of the peptide (compare supplementary Fig. 7Aa and 7Ab). The expression of most of the lncRNAs tested was also impacted by E2F1, displaying either increased (*AC004943.2*, *PPM1F-AS1*, *AC018445.6*) or decreased (*C5orf34-AS1*, *RP11-319G6.1*, *AC079135.1*) expression in the E2F1 KO cell line (supplementary Fig. 7A).

We confirmed that in human cancer cells, many of the peptide-encoding lncRNAs represent E2F1-target genes. We used ChIP-seq data to identify E2F1 binding sites, designed primers surrounding these sites and then by ChIP confirmed the presence of chromatin-bound E2F1 (supplementary Fig. 7B). E2F1 was observed to be enriched at the promoters of many of the lncRNA genes that produced peptides; around 44% of lncRNAs appeared to be potential direct E2F1 target genes, whilst a further 26% of peptide-encoding lncRNA genes were associated with other predicted E2F1 target genes (Fig. 4E).

Numerous human MHC bound peptides were derived from ORFs with less than 100 residues coding capacity, with a weak translation initiating sequence (supplementary Fig. 7C). We chose *MALAT1* and *AC079135.1* to investigate further and performed polysome profiling assays; RNA derived from both lncRNA genes could associate with translating polysomal ribosomes (Fig. 4F, with other lncRNA examples in supplementary Fig. 7D). We then addressed whether RNA derived from *MALAT1* and *AC079135.1* could be translated by cloning the cDNA (together with upstream sequence that should contain the intrinsic ribosome binding site) into an expression vector. Small proteins of the expected size for the ORF (containing the MHC-associated peptide) could be detected by immuno-staining and immunoblotting (Fig. 4G).

### Tumour growth inhibition with lncRNA-derived peptide vaccines

**Fig. 5:**
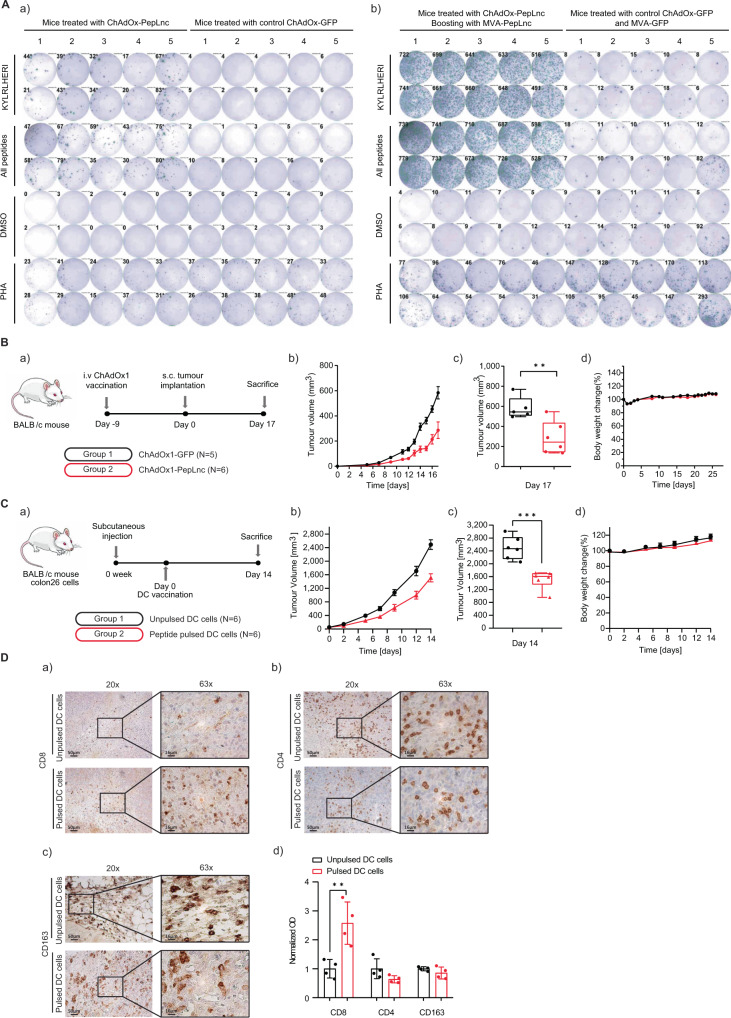
LncRNA derived MHC class I peptides as cancer vaccines in a colon26 tumour model. **A** Groups of 10 BALB/c mice were vaccinated with ChAdOx1-PepLnc adenoviral vectors expressing a poly-antigen cassette containing 20 lncRNA-derived peptides, or a control ChAdOx1-GFP adenoviral vector. At 9 days post vaccination, half the mice were sacrificed and their splenocytes collected for ELISpot assay (a). The other half received a booster vaccination 4 weeks later with MVA-PepLnc or MVA-GFP as indicated, for a further 9 days (b). Splenocytes were stimulated with the indicated peptide, or a pool of peptides contained within the poly-antigen cassette, and activity was measured in interferon-γ-based ELISpot. DMSO and PHA were used as negative and positive controls respectively; n = 2 independent experiments. **B** (a) BALB/c mice were vaccinated with ChAdOx1-PepLnc or ChAdOx1-GFP adenoviral vectors. At day 9, post-vaccination mice were challenged with CT26 cells; *n* = 5 ChAdOx1-GFP, *n* = 6 ChAdOx1-PepLnc. (b) Absolute tumour volume is presented as mean ± SEM, *n* = 5 ChAdOx1-GFP, *n* = 6 ChAdOx1-PepLnc; (c) Absolute tumour volume of individual mice at day 17 (two-tailed Student’s t test; ** *p*  =  0.0067), *n* = 5 ChAdOx1-GFP, *n* = 6 ChAdOx1-PepLnc; box and whiskers are defined as minimum, first quartile, median, third quartile, and maximum of data. (d) Relative body weight of BALB/c mice presented as a mean value, *n* = 5 ChAdOx1-GFP, *n* = 6 ChAdOx1-PepLnc. **C** (a) Colon26-bearing BALB/c mice were vaccinated with DCs pulsed with pools of 15 lncRNA derived peptides at Day 0. As a control, unpulsed dendritic cells were used; *n* = 6 mice per treatment. (b) Absolute tumour volume in BALB/c mice presented as mean ± SEM, *n* = 6 mice per treatment; (c) Absolute tumour volume of individual mice at day 12 (two-tailed Student’s t test; *** *p*  = 0.0004), *n* = 6 mice per treatment; box and whiskers are defined as minimum, first quartile, median, third quartile, and maximum of data. (d) Relative body weight of BALB/c mice presented as a mean value ± SEM, *n* = 6 mice per treatment. **D** Immunohistochemical staining of anti-CD8 (a), anti-CD4 (b), or anti-CD163 (c) in colon26 tumours at 14 days post vaccination with pulsed DCs (see Fig. 5C). Original magnification, 20x, scale bar, 50 μm; and 63x; scale bar, 16 μm. *n* = 4 independent experiments; d) Optical density is presented as mean ± SD; two-tailed Student’s *t* test; *n* = 4 independent experiments (each performed on two separate slides); ** adjusted *P* value < 0.01.

Given the increased immunogenicity within the TME in T1-44 treated tumours, suggested by the infiltrating CD8 T lymphocytes (Fig. 1E), we tested whether the lncRNA-derived MHC class I bound peptides could in part be responsible and therefore examined their immunogenicity in mice. Twenty of the peptides encoded by murine lncRNA genes that were identified in the immunopeptidomics experiment were chosen for immunogenicity analysis based on their predicted high affinity for H-2 MHC class I proteins, low expression of the lncRNA gene in normal mouse thymocytes and differential regulation upon PRMT5 inhibition (Supplementary Fig. 8A, B). A poly-antigen cassette was designed to express the selected peptides, and the cassette cloned into the ChAdOx1 and modified Vaccinia Ankara (MVA) viral vectors (ChAdOx1-PepLnc and MVA-PepLnc respectively) for immunising mice^18–20^. For the first experiment, mice were immunised with the ChAdOx1-PepLnc vector when, at day 9, splenocytes were harvested and IFN γ production measured by ELISpot (Fig. 5Aa, Supplementary Fig. 8C). When splenocytes were re-stimulated with the pool of peptides corresponding to those included in the poly-antigen PepLnc cassette, a robust IFN γ response was apparent, reflecting activated CD8 T lymphocyte cells, as compared to mice immunised with a control ChAdOx-GFP viral vector (Fig. 5Aa, supplementary Fig. 8C). The level of immunogenicity could be further enhanced when the first ChAdOx1-PepLnc immunisation was followed 28 days later by a booster vaccination with MVA-PepLnc (Fig. 5Ab, supplementary Fig. 8D). These results indicate that lncRNA-derived MHC class I bound peptides are immunogenic and stimulate an adaptive antigen-specific T lymphocyte response in mice. We noted that the most immunogenic peptides included in the poly-antigen cassette were derived from lncRNAs that exhibited a trend towards low expression in normal thymocytes, as compared to other tissues (Supplementary Fig. 8B).

We progressed on to test whether the T lymphocyte activity against the lncRNA-derived peptides could translate into a therapeutic benefit when they were delivered in the context of a cancer vaccine, namely whether the peptide vaccine enabled an anti-tumour immune response. We took two separate approaches. The first used a prophylactic vaccination strategy with the ChAdOx1-PepLnc or ChAdOx1-GFP control vector, when after 9 days CT26 cells were implanted subcutaneously into the mice (Fig. 5B). The second approach used an ex vivo dendritic cell (DC) delivery platform, where bone marrow DCs were harvested from mice, matured and then pulsed with the pooled peptides^21,22^. After 7 days, the peptide-pulsed and control dendritic cells were introduced into BALB/c mice with established syngeneic colon26 tumours (Fig. 5C). In both experimental settings, we measured the effect on tumour growth. Strikingly, vaccination with the ChAdOx1-PepLnc vector or transfer of the peptide-pulsed dendritic cells delayed growth of the tumours compared to the control groups vaccinated with ChAdOx1-GFP or treated with unpulsed dendritic cells (Fig. 5B, C). Significantly, the DC peptide vaccine-treated animals exhibited increased levels of CD8 T lymphocytes in the TME whereas the level of CD4 T lymphocytes and tumour-associated macrophages remained unchanged (Fig. 5D). In the context of dendritic cell delivery, lncRNA derived peptides were able to stimulate an effective CD8 T cell response and hinder the growth and thus provide a therapeutic benefit.

### Relevance to human cancer

It is noteworthy that some of the PRMT5-E2F1 responsive lncRNAs which encode MHC bound peptides, like *MALAT1* and *DANCR*, are already known to exhibit deregulated expression in human cancer^23^. We, therefore, evaluated the expression patterns of some of the less well characterised peptide-encoding lncRNAs identified here. As part of this exercise, we confirmed *MALAT1* and *DANCR* expression across a range of cancers and normal tissue (Supplementary Figs. 9 and 10). The expression pattern of other lncRNAs was variable; for example, *VPS9D1-AS1* exhibited heterogenous expression, with high expression in some cancers and generally low expression in normal tissue. This contrasted with *CTC-459F4* which had uniformly low expression in cancer and normal tissue. It was noteworthy that human lncRNAs which encoded MHC class I bound peptides exhibited a similar expression profile to murine lncRNAs, namely low expression in normal thymus (Supplementary Fig. 10).

In a detailed analysis across a range of CRC tumour cell lines, some lncRNA genes exhibited high and others low expression (Supplementary Fig. 9). Interestingly, when lncRNA expression was analysed in human colorectal, stomach and oesophageal cancer, there was clear differentiation between expression in the micro-satellite stable (MSS) and micro-satellite instable (MSI) sub-groups; for example, in colorectal cancer the majority of lncRNA expression occurred in the MSS sub-group and not the MSI sub-group, which was less marked in stomach and oesophageal cancers (Supplementary Fig. 9). Generally, therefore, the expression profile of the lncRNAs that encode MHC class I associated peptides is influenced by the type of cancer and the stage of disease.

## Discussion

Genomic analysis has suggested that the human genome harbours a diverse and extensive group of lncRNA genes^2,24^. A relatively small number of lncRNAs have been described to be processed and spliced in a similar way to mRNA^2^. Whether lncRNAs are biologically important remains a widely debated topic; some lncRNAs have been ascribed cellular functions, for example, in chromatin biology^25,26^ and other studies implicate lncRNAs in RNA biogenesis^2^. Moreover, some lncRNA genes have been connected with cancer, such as *MALAT1*, which is a highly conserved lncRNA that is abundantly expressed in cells, and was initially identified as exhibiting elevated expression in metastatic lung cancer^27^. In a murine metastatic cancer model, loss of *MALAT1* resulted in differentiation of primary tumours and a significant reduction in metastasis^28^. Additionally, *MALAT1* RNA has been suggested to play a role in regulating genes at both the transcriptional and post-transcriptional levels^29,30^.

**Fig. 6:**
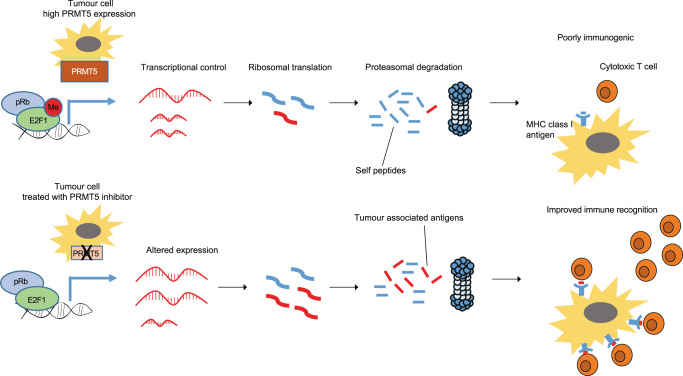
Model diagram to indicate regulation of lncRNA-derived antigen presentation by E2F1 and PRMT5. It is proposed that PRMT5 enzyme activity and E2F1 transcriptional activity influences the expression of many lncRNAs, which are subsequently translated into polypeptides that can be processed to generate epitopes for presentation on MHC class I protein complexes. Pharmacological manipulation of PRMT5 activity with compound T1-44 results in altered expression of several lncRNA transcripts encoding immunogenic peptides. We propose that subsequent presentation of these immunogenic peptides by MHC class I complexes contributes to the increased immune cell infiltration of the tumour micro-environment observed.

Our study has connected the pRb-E2F pathway and PRMT5, a key cancer-relevant enzyme, with control of lncRNA gene expression. The pRb-E2F pathway is a central regulator of cell growth and division and represents one of the principal pathways that is subjected to oncogenic de-regulation in human cancer. PRMT5 expression is frequently elevated in cancer, where its activity is integrated with E2F1 through a methylation event which expands the target genes under E2F control^8^. Given the over-expression of PRMT5 in many cancers^10,31–33^ combined with the frequent if not universal de-regulation of the pRb-E2F pathway^6^, the intersection of these two central regulators is highly likely to be important in driving the malignant phenotype. Consistent with this idea, we have here extended the role of PRMT5 and E2F1 by showing that they take on a regulatory role in the non-coding genome. One of the important findings identified many lncRNAs, under PRMT5 and E2F1 control, to encode peptides that assemble with MHC class I proteins. The results, therefore, highlight the interplay between the E2F pathway and PRMT5, with antigen presentation by tumour cells to the immune system (Fig. 6).

Our conclusions reflect a thorough immunopeptidomic analysis of the peptide composition of MHC class I proteins on cancer cells, performed on both human and mouse cells, which identified a significant proportion of peptides that are encoded by lncRNA genes. Many of these lncRNA genes are regulated upon pharmacological inhibition of PRMT5 in addition to being direct E2F1 target genes. It is through this interplay that both PRMT5 and E2F1 are able to regulate antigen presentation by cancer cells. Interestingly, *MALAT1* was one of many lncRNAs that we found to encode an antigenic peptide, thus extending the significance of *MALAT1* in cancer by connecting its lncRNA expression with a derived peptide and antigen presentation. Whilst we cannot currently comment on the potential immunogenicity of this MALAT1 derived peptide, other studies where bioinformatics approaches have been deployed suggested that tumour associated antigens can be derived from non-canonical parts of the genome although, to our knowledge, this concept remains to be proven^34–38^.

LncRNA genes are a heterogeneously expressed family of genes, which is particularly apparent in cancer^39^. In the context of the results described here, the altered lncRNA expression seen across diverse tumours may translate into differences in the repertoire of antigenic peptides (encoded by lncRNA genes) presented to the immune system by the MHC complex. It is noteworthy however that the peptides derived from lncRNA genes are self-antigens and therefore, theoretically, T cells directed against such peptide antigens should be eliminated during development or the immune response suppressed through other mechanisms^40^. Many of the peptides that we tested in mice could drive an antigen-specific T cell response when delivered as a vaccine, suggesting that any immunological mechanisms that do exist to suppress the immune response against this type of self-antigen can be over-ridden, and it will be interesting to establish whether a similar phenomenon exists in humans. Interestingly, it appears that the most immunogenic peptides exhibited a trend towards low expression in normal thymocytes. This could suggest that the peptides escape central tolerance (namely clonal negative selection) in the thymus due to low expression and are perhaps subjected to peripheral tolerance mechanisms. Indeed, there are numerous reports of immunisation regimes that produce a favourable immune response against self-antigens with anti-tumour effects^41,42^. In fact, we gained evidence for a strong antigen-specific T lymphocyte response against the lncRNA-derived peptides in vaccinated mice, which led us on to test whether the adaptive T cell immunity would translate into an immune response against tumours. Remarkably, in the colon26 syngeneic mouse model, lncRNA derived peptides delivered through ex vivo peptide loaded dendritic cells or directly by the ChAdOx1 viral vector platform could stimulate an immune response which delayed tumour growth. This is the first demonstration that a cancer vaccine, derived from genes within the non-coding genome, can be engineered and clinically delivered to create an effective anti-cancer immune response.

Our results highlight the non-coding genome as an unanticipated rich source of tumour associated antigens that can be presented to the immune system through the classical route of MHC class I associated peptides (Fig. 6). The ability to unlock their expression through pharmacological manipulation of PRMT5 and E2F1 activity enables what is potentially a powerful therapeutic approach to control the immunogenicity of tumour cells. Ultimately, this information could allow us to engineer effective cancer vaccines that are aligned, through manipulating antigens derived from the non-coding genome, to a specific type of cancer.

## Methods

### Cell line generation, culture and compound treatments

Generation of human p53-/- HCT116 E2F1 CRISPR and CAS9 control cells have been described previously^11^. Mouse CT26 cells were acquired from ATCC (CRL-2638) and were used in culture and with the ChAdOx1-based vaccine tumour challenge experiment. The genetically similar colon26 cells were used by Charles Rivers Laboratories for out-sourced tumour challenge models. Cells were cultured in Dulbecco’s modified Eagle medium (DMEM) (Sigma-Aldrich) supplemented with 10% foetal bovine serum (Labtech) and 1% penicillin/streptomycin (Gibco). All cell lines were tested for mycoplasma contamination before use. Selective PRMT5 inhibitor (T1-44) (synthesised by Argonaut Therapeutics) has been described and characterised previously^11^ and was used for 48 h at 1 μM final concentration unless otherwise stated. For comparison, established inhibitors of PRMT5; JNJ-64619178 and LLY-283 were used (Selleck).

### Plasmid/siRNA transfections

Plasmid transfections were performed for 48 h using the GeneJuice transfection reagent (Novagen), as per the manufacturer’s instructions. RNA interference was performed with 25 nM siRNA for 72 h using the Oligofectamine transfection reagent (Invitrogen), as per the manufacturer’s instructions. Sequences for siRNA are as follows: nontargeting control, 5′-AGCUGACCCUGAAGUUCUU-3′; E2F1 (human and mouse), 5′-CUCCUCGCAGAUCGUCAUCUU-3′; E2F1 (mouse) (EMU075181, Merck).

### Immunoblots and antibodies

For immunoblots, cells were harvested in modified RIPA buffer (50 mM tris-HCl pH 7.5, 150 mM NaCl, 1% Igepal CA-630 [v/v], 1 mM EDTA, 1 mM NaF, 1 mM Na3VO4, 1 mM AEBSF, protease inhibitor cocktail) and incubated on ice for 30 min prior to SDS–PAGE and transfer to nitrocellulose. The following antibodies were used in immunoblots: β-actin (clone AC-74, Sigma-Aldrich; dilution 1:2000), E2F1 (3742S, Cell Signaling Technology, dilution 1:1000), symmetric di-methyl arginine (SDMe) (13222S, Cell Signaling Technology, dilution 1:1000), FLAG (clone M2, F1804, Sigma, 1: 1000), GAPDH (clone 6C5, MAB374, Millipore, 1:2000). Uncropped versions of immunoblots are presented in the supplementary figure 11.

### In vitro T1-44 methyltransferase specificity screen

The in vitro methyltransferase screen (AMS Biotechnology Europe) was performed to determine any off-target effects of compound T1-44 (10 µM) on the enzymatic activities of other arginine and lysine methyltransferases. The assay was performed in duplicate and reference inhibitor compounds were included as controls for each enzyme. All reactions were conducted in wells of a plate pre-coated with the appropriate substrate, and were performed at room temperature for 60–960 min in 50 µl reaction volumes containing methyltransferase assay buffer, S-adenosylmethionine (SAM), enzyme, and the test compound. Enzyme and inhibitor were added first to the assay wells and pre-incubated for 30 min, before the addition of SAM. After the enzymatic reaction was performed, each well was washed three times with TBS-T, before blocking for 10 min in blocking buffer. 100 μl of diluted primary antibody was added and incubated for 60 min. Plate wells were washed three times in TBS-T, and blocked again for 10 min prior to addition of 100 µl of diluted secondary antibody for 30 min. The plate was then washed and blocked as before prior to addition of 100 μl HRP chemi-luminescent substrate. Sample luminescence was measured in a Synergy 2 microplate reader (BioTek).

### RNA isolation and quantitative RT-PCR

RNA was isolated from cells using TRIzol (Thermo Fisher Scientific) or the Direct-zol RNA MiniPrep kit (Zymo Research) according to the manufacturer’s instructions. 1 μg of total RNA was used for complementary DNA (cDNA) synthesis. Reverse transcription with oligo (dT)20 (Invitrogen) was performed using SuperScript III Reverse Transcriptase (Invitrogen) as per the manufacturer’s instructions. Quantitative PCR (qPCR) was then carried out in technical triplicate using the indicated primer pairs and the Brilliant III SYBR Green qPCR Master Mix (Stratagene) on an AriaMx (Agilent) instrument. Results were expressed as average (mean) fold change compared to control treatments using the ΔΔCt method from at least three biological repeat samples. Glyceraldehyde-phosphate dehydrogenase (GAPDH) primer sets were used as an internal calibrator. Error bars represent SD unless otherwise indicated. For primer lists, please see Supplementary Table S1.

### RNA sequencing

WT E2F1, E2F1 Cr HCT116, and CT26 cells were treated with 1 µM concentration of PRMT5 inhibitor (T1-44) or DMSO as a negative control, for 48 h (HCT116) or 72 h (CT26). Total RNA from WT E2F1, WT E2F1 T1-44, E2F1 Cr, E2F1 Cr T1-44, CT26, and CT26 T1-44 (biological triplicates) was isolated using Direct-zol RNA MiniPrep kit (Zymo Research) according to the manufacturer’s instructions. Alternatively, RNA isolated from mouse tumours in situ was used for RNA-seq analysis. RNA-sequencing was performed by BGI Genomics. Briefly, an Agilent 2100 Bioanalyzer (Agilent RNA 6000 Nano Kit) was used for RNA sample quality control purposes (RNA concentration, RIN value, 28S/18S, and the fragment length distribution). mRNAs were isolated from total RNA using the oligo(dT) method. Then the mRNAs were fragmented, and first strand/second strand cDNA were synthesised. cDNA fragments were purified and resolved with EB buffer for end reparation and single nucleotide A (adenine) addition. Subsequently, the cDNA fragments were linked with adaptors. Those cDNA fragments with suitable size were selected for the PCR amplification. An Agilent 2100 Bioanalyzer and ABI StepOnePlus Real-Time PCR System were used in quantification and qualification of those libraries. The RNA sequencing was carried out using Illumina HiSeq Platform, and 5.12 Gb per sample was generated. RNA sequencing data from the HCT116 cells have previously been published^11^ (Gene Expression Omnibus (GEO) under accession code GSE142430).

### Long non-coding RNA (LncRNA) expression data processing

FASTQ files for WT E2F1, E2F1 Cr HCT116, and CT26 cells treated with PRMT5 inhibitor or DMSO control were generated from three biological repeat experiments. These were trimmed to remove adaptors and low-quality bases with TrimGalore v.0.4.3 (http://www.bioinformatics.babraham.ac.uk/projects/trim_galore/).

LncRNA expression analysis was performed using Kallisto (v. 0.44.0) with k-mer length 31 and 100 bootstrap samples (WT E2F1, E2F1 Cr HCT116, and CT26 cells). GENCODE mouse lncRNA annotation version M22 and human lncRNA annotation version 34 were used as a reference dataset to construct kallisto indices. Differential expression of lncRNA was computed with sleuth package (v 0.30.0). The log2 (fold-change) in expression was computed from estimated counts values (provided by kallisto) averaged across all replicates for a given condition. Significantly, differentially expressed transcripts were identified using FDR threshold (q-value) of 0.05. Sequencing reads for colon26 tumour tissue experiments were aligned to the mm10 version of the mouse genome with STAR (version 020201) using GENCODE mouse lncRNA annotation version M22. Differential gene expression analysis was conducted with DESeq2 R Bioconductor package (v.1.25.17). Significant differentially expressed genes were identified using FDR threshold (adjusted *p* value) of 0.05. In all lncRNA data processing, the Benjamini-Hochberg procedure was used to correct for multiple testing.

HCT116 p53-/- and HCT116 p53-/- E2F1 Cr RNA-seq datasets have been deposited to the GEO under accession code GSE142430. CT26 and colon26 tumour sample RNA-seq datasets have been deposited to GEO under accession code GSE181401.

### Data processing for liquid chromatography mass-spectrometry analysis (lncRNA-derived peptide databases)

Nucleotide sequences of all lncRNAs expressed at detectable levels in our HCT116 and CT26 RNA-seq datasets (mouse CT26: annotated with GENCODE; human HCT116: annotated separately with FANTOM 5 and GENCODE) were converted into peptide sequences using 3-frame translation. The peptide sequence data were broken down into 3 groups, in accordance with expression values of corresponding lncRNAs: non-expressed (TPM = 0); weakly expressed (0.5 <TPM < 1.0 or ‘low’); expressed (TPM > 1.0 or ‘high’). The non-expressed group were used as a decoy database for MS proteomic experiments (please see ‘Mass spectrometry data analysis’ subsection). For databases used, please see Supplementary data 8–10.

### HLA class I immunoprecipitation

Antibodies were sourced from hybridoma supernatants (ATCC, HB-95 and −79, respectively) using a standard purification procedure using Sepharose-protein A beads (Expedeon). 0.5 ml/sample beads were incubated with 5 mg/sample of W6/32 antibody (specific for HLA class I for HCT116), or antibody clone 34.1.2 s (recognising H-2 K^d^, D^d^, L^d^ for CT26), for 30 min at room temperature. The resin was washed with 10 cv (column bed volumes) of borate buffer (50 mM borate, 50 mM KCl, pH 8.0) and antibodies were cross-linked by adding 10 cv of 40 mM dimethyl pimelimidate in borate buffer (pH 8.3) for 30 min at room temperature. The reaction was stopped with 10 cv of ice-cold 0.2 M Tris, pH 8.0, followed by a washing step of 10 cv of 0.1 M citrate, pH 3.0, to remove any unbound antibody, and finally equilibrated with 10 cv of 50 mM Tris, pH 8.0.

Cell pellets were lysed in 3 ml lysis buffer (1% Igepal CA-630; 100 mM Tris, pH 8.0; 300 mM NaCl; supplemented with complete Protease Inhibitor Cocktail, EDTA-free, [Roche]) by mild agitation. Samples were incubated for 45 min on ice. Lysates were then cleared by sequential centrifugation steps at 500 *g* for 10 min then 20,000 *g* for 1 h at 4 °C. Peptide-HLA class I complexes were captured by overnight incubation with the antibody-coated beads at 4 °C under mild agitation. The lysate was then removed by gravity flow, and the column was washed consecutively with 10 ml wash buffer 1 (0.005% Igepal, 50 mM Tris pH 8.0, 150 mM NaCl, 5 mM EDTA), 10 ml wash buffer 2 (50 mM Tris pH 8.0, 150 mM NaCl), 10 ml wash buffer 3 (50 mM Tris pH 8.0, 450 mM NaCl) and 10 ml wash buffer 4 (50 mM Tris pH 8.0). Peptide-HLA complexes were eluted by addition of 5 cv of 10% acetic acid.

### HLA peptide purification strategies

Samples were loaded onto a Ultimate 3000 HPLC system (ThermoFisher Scientific) and peptides were separated from larger complex components using a monolithic column (4.6 × 50 mm ProSwift RP-1S, ThermoFisher Scientific) by applying a 10 min gradient from 2 to 35% buffer B (0.1% TFA in acetonitrile) with a flow rate at 1000 µl/min. Each sample was fractionated in 15 fractions and alternate fractions containing the HLA peptides but not ß2-microglobulin, were pooled in two final fractions. Samples were dried, re-suspended in 20 µl of loading buffer (0.1% TFA, 1% ACN) and stored at −80 °C prior to MS analysis.

### LC-tandem mass spectrometry (LC-MS/MS)

For HCT116 cell samples, HLA peptides were analysed by either an Orbitrap Fusion Lumos Tribrid mass spectrometer (Thermo Scientific) or a Q Exactive HF-X mass spectrometer (Thermo Scientific). CT26 cell samples were measured on a Q Exactive HF-X (Thermo Scientific). Either mass spectrometer instrument was coupled with an Ultimate 3000 RSLCnano System supplemented with a PepMap C18 column, 2 µm particle size, 75 µm × 50 cm (Thermo Scientific). Peptides were eluted using a 60 min linear gradient of 3% to 25% acetonitrile in 5% DMSO, 0.1% formic acid in water at flow rate of 250 nl/min and 40 °C, and introduced into the mass spectrometer using a nano EASY-Spray source at 2000 V (Thermo Scientific). The ion transfer tube was set to 305 °C for both instruments.

For samples analysed by the Orbitrap Fusion Lumos, the resolution for full MS was set at 120,000 with ACG target of 400,000 and scan range of 300–-1500 m/z. Precursor selection and isolation were performed using TopSpeed in a 2 s cycle time and 1.2 amu quadrupole isolation width. MS2 resolution was set at 30,000 and peptide ions were accumulated at a maximal injection time of 120 ms with an AGC target of 300,000. Precursor ions were fragmented using high-energy collisional dissociation: Collision energy was set to 28 for peptides with charge state of 2–4, and set to 32 for singly-charged ions. For samples analysed on the Q Exactive HFX, full MS (320–1600 m/z scan range) resolution was set at 120,000, and an AGC target of 300,000. Peptide ions were isolated at 1.6 amu isolation width. MS2 resolution was set to 60,000 at an AGC target of 50,000 and the collision energy was set at an energy of 28 for peptides with a charge state of 2–4 fragmentation of precursor ions and 25 for those with a charge state of 1–4.

### Mass spectrometry data analysis

MS data were analysed with Peaks v8.5 (Bioinformatics Solutions) for identification of peptide sequences matching to databases generated by integration of all reviewed human SwissProt protein entries (20,413 entries, current at 22/01/2019), or all reviewed mouse SwissProt protein entries (17,019 entries, current at 17/07/2019), combined with the respective three frame translations of the open reading frames obtained from the in-house RNA sequencing assemblies generated for the HCT116 data and CT26 cell lines, respectively. Searches were performed with the following parameters: no enzyme specificity, no peptide modifications, peptide tolerance: ±5 ppm and fragment tolerance: ±0.03 Da. The results were filtered using a false discovery rate of 4.3% and 5.8% established through parallel decoy database searches for HCT116 and CT26 data, respectively. For quantitative analysis, the data were analysed by Progenesis QI v2.0 for proteomics (Waters). A one-way ANOVA analysis was applied to assess significant regulation of peptides between conditions. GraphPad Prism 8 (GraphPad Software Inc), and Bio Venn (http://www.biovenn.nl/) were used for visualisation of the data. HLA class I peptides prediction was performed using NetMHC4.0 online algorithm^43^ and Seq2Logo2^44^ or WebLogo^45^. HCT116 and CT26 datasets are available via ProteomeXchange (PRIDE database) with identifiers PXD029613 and PXD029594, respectively. In CT26, between the qualitative (328 peptides) and quantitative (195 peptides) analysis, 382 unique lncRNA-derived peptide identifications were detected. In HCT116, between the qualitative (FANTOM5 and GENCODE annotated) (55 peptides) and quantitative analysis (76 peptides), 118 unique lncRNA-derived peptide identifications were detected.

### Chromatin immunoprecipitation (ChIP)

E2F1 ChIPs were performed as described previously^46^, using 3 μg of appropriate antibody (control rabbit IgG, anti-E2F1 [A300-766A], Bethyl Laboratories) and pre-blocked protein A beads. The recovered DNA was purified and real-time PCR was performed in triplicate with Brilliant III Ultra-Fast SYBR green QPCR master mix on an AriaMx QPCR instrument (Agilent) using primers flanking proposed E2F sites in gene promoters. DNA occupancy was investigated by calculating the percentage enrichment of input for both the E2F1 ChIP and IgG controls from triplicate biological repeat experiments. In all cases, the presented figure displays SD unless otherwise stated. The CDC6 and actin promoters were used as positive and negative control for E2F1 occupancy, respectively. For primer lists, please see Supplementary Table S2.

### Human and mouse lncRNA promoter analysis

LncRNA gene promoter characterisation was performed utilising bioinformatics tools present in UCSC Genome Browser (https://genome.ucsc.edu; GRCh37/h19 assembly) and analysing ChIP-seq data for E2F tracks from the ENCODE project (http://genome.ucsc.edu/ENCODE/) for three cell lines (K562, MCF7, HeLa). The ‘Transcription factor ChIP-seq clusters from ENCODE 3′ (ENCODE Regulation Txn Factr ChIP E3 Track Settings (ucsc.edu)), ‘Transcription factor ChIP-seq clusters from ENCODE with factorbook motifs’ (ENCODE Regulation Txn Factor ChIP Track Settings (ucsc.edu)), ‘Transcription factor ChIP-seq peaks from ENCODE 3′ (ENC TF Binding ENCODE 3 TFBS Track Settings (ucsc.edu)), ‘Transcription factor ChIP-seq uniform peaks from ENCODE/Analysis’ (ENC TF Binding Uniform TFBS Track Settings (ucsc.edu)) and ‘Transcription factor binding sites by ChIP-seq from ENCODE/Stanford/Yale/USC/Harvard’ (ENC TF Binding SYDH TFBS Track Settings (ucsc.edu)) track tools were used to display E2F1 ChIP-seq peaks or signal as appropriate. Genes were scored as potential direct E2F1 targets if ChIP-seq peaks were apparent within 1000-bp regions centred upon the annotated transcript start site (TSS) (i.e., 500 bp either side) (annotated by GENCODE and FANTOM6). LncRNA genes were scored as being associated with other E2F1 target genes if they overlapped the gene boundaries of a potential E2F1 target gene on the same or opposite strand, or were contained within the gene boundaries of an E2F1 target gene on the same or opposite strand. For promoter characterisation in mouse, the GRCm38/mm10 assembly was used, and mouse E2F1 ChIP-seq peak data was loaded as a custom track using data deposited in GEO (GSM288349). ChIP-seq peak coordinates were intersected with 1000 bp wide regions around lncRNA TSS (GENCODE annotation).

### Polysome profiling

Cells were treated with 100 mg/ml cycloheximide for 10 min at 37 °C, treated with 1x trypsin-EDTA solution for 10 min and washed twice with ice cold 1X PBS containing 100 mg/ml of cycloheximide. Polysome lysis buffer composed of 20 mM Tris HCl pH 7.4, 5 mM MgCl_2_, 100 mM KCl, 100 μg/mL cycloheximide, 1% Triton X-100, 1x RNase inhibitor (Invitrogen), and 1x protease inhibitors (VWR) was used to resuspend cells, followed by 30 min incubation on ice (occasional inverting) and 10 min centrifugation at 12,000 *g* at 4 °C. Sucrose gradients were prepared using 10% and 50% sucrose solutions (sucrose diluted in polysome extraction buffer without Triton X-100 and prepared in RNAse-free conditions) in polypropylene, 13.2-ml tube (Beckman Coulter). The gradient was left at 4 °C overnight to become linear. Clear supernatants from lysed cells were loaded (300 µg of RNA measured by Nanodrop [Thermo Fisher Scientific]) onto the 10–50% sucrose gradients and centrifuged at 190,000 *g* (SW40Ti rotor, Beckman Coulter Optima XE) for 90 min at 4 °C. Twelve sucrose gradient fractions were separated using manual collection, and the absorbance was measured at 254 nm to record the polysome profile.

### LncRNA ORF cloning strategy

For those lncRNAs identified in HCT116 and CT26 cells as giving rise to MHC class I peptides, the lncRNA transcripts were translated in all three frames and potential open reading frames (ORFs) were identified by highlighting all sequences contained between every ATG codon (encoding a start methionine) and a subsequent in frame STOP codon. Any potential ORF that would generate a poly-peptide that contained the identified MHC peptide was identified as a sequence for cloning into a plasmid vector expressing a C-terminal FLAG tag (pSF-CMV-NEO-COOH-3xFLAG; OG629, OxGene). Primers were designed to amplify the ORF (minus the STOP codon) and 30 bp upstream sequence (to include any inherent ribosome binding site present in the endogenous transcript. Note that a ribosome binding site is not provided by the vector itself) and contained restriction sites for *EcoRI/XhoI* and *SacI/EcoRV* as appropriate (for primer lists, please see Supplementary table S2). A PCR reaction was performed using Phusion High Fidelity DNA Polymerase (M0530S, New England Biolabs) and cDNA from HCT116 or CT26 cells as a template (generated as described above for quantitative RT-PCR). PCR products were purified using a QIAquick PCR purification Kit (Qiagen) and digested with *EcoRI/XhoI* and *SacI/EcoRV* (Promega) as appropriate. Digested products were gel purified using a QIAquick Gel Extraction Kit (Qiagen) prior to ligation into digested vector using T4 DNA ligase (New England Biolabs). All plasmids were sequenced to confirm correct cloning prior to use in transfections.

### Functional genomics analysis—TCGA

For the analysis of peptide coding lncRNA transcript expression levels in human cancers, Xena browser v1 (University of California; https://xena.ucsc.edu/) and GEPIA v2 (http://gepia2.cancer-pku.cn/) were used. The TCGA TARGET GTEx (Xena browser) dataset was selected, which contained transcript expression data from TCGA (cancer tissue; https://www.cancer.gov/about-nci/organization/ccg/research/structural-genomics/tcga) and Genotype-Tissue Expression (GTEx; healthy tissue; https://gtexportal.org/home/) samples. For subsequent detailed analysis of microsatellite instability and staging, datasets from TCGA were used. Also, Broad Institute Cancer Cell Line Encyclopedia v1 (portals.broadinstitute.org › ccle) was used to analyse the expression of lncRNA genes in colorectal cancer cell lines. Heatmaps were generated using Heatmapper tool v1 (http://heatmapper.ca/).

### Genevestigator analysis

For the normal tissue and thymocyte expression analysis of murine lncRNAs giving rise to peptides, the Genevestigator tool v9.7.0 (Nebion AG) was used. Data from refs. ^47–50^ were collected and presented as heat maps generated using Morpheus software v1 (Broad Institute; https://software.broadinstitute.org/morpheus/).

### Colon26 mouse tumour model with T1-44 treatment

All experiments and protocols were approved by the Charles River Animal Care and Use Committee at Charles River Discovery Research Services Germany (where each experiment was performed) and the National Committee for the Protection of Animals Used for Scientific Purposes for the Federal Republic of Germany. Housing conditions: temperature: 22–24 °C, 12 h day/night cycle, Humidity 40–70%. Fourteen female BALB/c mice at 6–8 weeks of age (7 mice per group: control and treated) (Charles River Laboratories, Germany) received unilateral subcutaneous injections of 5 × 10^5^ colon26 cells in PBS in a total injection volume of 100 µl/mouse. Upon reaching individual tumour volumes of 50–150 mm^3^, mice were assigned to treatment groups based on tumour volumes aiming at comparable group mean/median tumour volumes. Within 24 h of randomisation, mice were daily treated by oral administration (gavage) with 100 mg/kg (dosing volume 10 ml/kg) of T1-44 using 0.5% Tween/PBS as a vehicle. Body weights and tumour volume [mm^3^] by caliper measurement were performed twice weekly. Termination of individual mice was conducted at day 19 of the experiment or at >1000 mm^3^ (unilateral) volume, in case of tumour ulceration or body mass loss at <70% of initial weight. From each group, four snap frozen tumours were collected for RNA isolation and four formalin-fixed samples were prepared for immunohistochemical staining. From this experiment, serum was collected from each mouse, and the panel of twelve cytokines (IFN γ, IL-2, IL-4, IL-5, IL-13, IL-10, IL-9, TNFα, IL-6, IL-17A, IL-17F, IL-22) was analysed using LEGENDplex MU Th Cytokine Panel (12-plex) VbP V03 according to manufacturer’s instruction (741044, Biolegend).

### ChAdOx1 and MVA preparation

The ChAdOx1-PepLnc, ChAdOx1-GFP, MVA-PepLnc and MVA-GFP adenoviruses were manufactured by Viral Vector Core Facility (Jenner Institute, University of Oxford). The poly-antigen cassette, containing the MHC class I presented peptides derived from lncRNAs (Supplementary Fig. 8A) was designed to include each 9-mer sequence plus 24 base pairs of natural flanking upstream and downstream sequence; i.e. for RGPSHFSRL and KYLRLHERI peptides, sequences coding ITDPGTVP*RGPSHFSRL*PLGGWAED and CDKAFLKL*KYLRLHERI*YSGKKPY respectively, were designed. Coding sequences for all peptides were combined into the poly-antigen cassette. The tPA sequence was also added to the 5′ end of the cassette in addition to a Kozak sequence. The cassette was synthesised in the pMA-T-21AAXRHP plasmid (Thermo Fisher Scientific) by GeneArt Gene Synthesis. pMA-T-21AAXRHP and p1990 (the entry vector containing a long CMV promoter) plasmids were digested with *KpnI* and *NotI* to obtain the antigen insert and the entry backbone respectively. DNA was then ligated and transformed into DH10b cells. Bacterial clones were colony screened by PCR and a clone was selected for plasmid purification using midi-prep (Qiagen). This entry clone was named pENTR4LPTOS-PepLnc. pENTR4LPTOS-PepLnc and the destination shuttle vector p2563 were recombined using LR clonase II (Thermo Fisher Scientific) and transformed into DH10b cells. Clones were screened by antibiotic sensitivity and colony PCR to select a single clone for BAC prep. This resulting pChAdOx1-PepLnc plasmid was sequenced to confirm its identity and digested to linearise the plasmid, as per standard protocols. Linearised pChAdOx1-PepLnc was used for virus production in HEK293A T-Rex cells. The presence of the antigen was confirmed by ID PCR. The integrity of the antigenic DNA sequence and absence of contaminating Adenovirus was confirmed by Flank-Flank PCR.

For the MVA plasmid preparation, primers were designed to amplify the antigen from GeneArt plasmid pMA-T-21AAXRHP-PepLnc and the MVA shuttle p5586 (insertion site at the F11 loci under the F11 promoter with GFP) to generate homologous ends. These were recombined using NEB builder and transformed into DH5 alpha cells. Clones were screened by PCR and a single clone selected to amplify DNA using a midi prep kit (Qiagen). The resulting clone MVA-F11-PepLnc-GFP was sequenced to confirm its identity before linearization using *XhoI*, as per standard protocols. Plasmid MVA-F11-PepLnc-GFP was recombined with parental MVA-F11-mcherry. The cell lysate from this recombination was harvested and used to infect DF-1 cells. These cells were single-cell sorted into 96-well plates using a MoFlo cell sorter (Beckman Coulter) and used to culture recombinant virus upon addition of fresh DF-1 cells. Those wells containing suitably infected cells were harvested and screened by PCR to confirm identity and test purity. Plaque picking was performed until the culture was free of parental virus, as determined by PCR. To confirm the presence of the antigen in question and lack of parental virus contamination in the final stock, ID and purity PCR were performed. A PCR spanning the antigen insertion site was also performed to confirm the total length of the antigen and to detect any possible cross-contamination.

### ChAdOx1 and MVA immunogenicity

Ten 8-week old female BALB/c mice (Charles River Laboratories) were vaccinated i.v with ChAdOx1-PepLnc adenoviral vectors (5 × 10^8^ IU). Another ten control mice were vaccinated with ChAdOx1-GFP vector. At day 9 post-vaccination half of the mice in each group were culled and their spleens collected for ELISpot. The rest of the mice were boosted i.v. with MVA-PepLnc (or MVA-GFP control; both 1 × 10^7^ PFU) 4 weeks after prime vaccination. Mice were culled 9 days post-boost and their spleens removed. All animals were housed in specific pathogen-free conditions at the Biomedical Services Building (University of Oxford). All work was performed under UK Home Office license PPL PP3430109 in accordance with the UK Animal (Scientific Procedures) Act 1986. All work was performed by trained and licensed individuals. We performed the experiment twice with successful replication. In the manuscript, we are presenting one representative experiment.

### ChAdOx1 tumour challenge experiment

Six female BALB/c mice at 6–8 weeks of age (Charles River Laboratories) were vaccinated i.v with ChAdOx1-PepLnc adenoviral vectors (5 × 10^8^ IU). Another six control mice were vaccinated with ChAdOx1-GFP vector. At day 9 post-vaccination the mice received unilateral subcutaneous injections of 5 × 10^5^ CT26 cells in PBS in a total injection volume of 100 µl/mouse. Body weights and tumour volume [mm^3^] by caliper measurement were performed twice weekly. After reaching 100 mm^3^ tumour volume (calculated according to formula: ((Length × width^2^)/2), mice were monitored daily. Termination of individual mice was conducted at day 17 post-implantation or at tumour volume not exceeding 1200 mm^3^ (unilateral). All animals were housed in specific pathogen-free conditions at the Biomedical Services Building (University of Oxford). All work was performed under UK Home Office license PPL PP3430109 (Protocol 2) in accordance with the UK Animal (Scientific Procedures) Act 1986 and was approved by The Committee on Animal Care and Ethical Review at the University of Oxford. All work was performed by trained and licensed individuals. Housing conditions: temperature: 22–24 °C, 12 h day/night cycle, Humidity 40–70%. We performed the experiment twice with successful replication. In the manuscript, we are presenting one representative experiment.

### ELISpot assay

The day before culling, Merk Multiscreen 96 well Filter Plates (Merck) were incubated with primary antibody (INFγ mAb clone AN18, Mabtech, 3321-3-1000) diluted 1:200 in sterile PBS (Gibco), at 4 °C. The next day, the antibody was removed, plates were washed four times with PBS at 250 µl/well, then blocked with 200 µl/well R10 (RPMI [Gibco] supplemented with 10% heat-inactivated FCS, Non-essential amino acids, L-Glutamine and penicillin/streptomycin (all from Sigma) for 2 h at 37 °C). Mice were culled, their spleens removed, and passed through a 40 µm cell strainer (Falcon) and the single cell suspension pelleted by centrifugation. The splenocytes were resuspended in 3 ml ACK lysis buffer (Lonza) for 3–5 min to lyse the red blood cells, then stopped with 20 ml PBS, followed by centrifugation at 400 *g*, 5 min at room temperature. The splenocyte pellet was resuspended in 5 ml R10, counted and the cell concentration adjusted to 4 × 10^5^/ml. Blocking buffer was removed and replaced with 50 µl of cells which were stimulated with the respective individual peptides (50 µl of peptide at 15 µg/ml) that the group had been vaccinated with. Each peptide was tested in duplicate. Negative control wells contained DMSO only while cells were stimulated with PHA-L (11249738001, Roche, dilution 1:200) as a technical control. The plates were incubated overnight (15–20 h) in a 37 °C (5% CO_2_) incubator. The cells and peptides were removed and the wells washed 7 times with sterile PBS. Secondary antibody (biotin conjugated anti-INFγ, MabTech, 3321-6-100, clone R4-6A2-Biotin) diluted 1:2000 in assay diluent (AD) (25 mg/ml BSA in PBS), was added (50 µl/well) and incubated for 2 h at room temperature. The plates were then washed four times with PBS then 50 µl of streptavidin-alkaline phosphatase (Mabtech, 3310-10-1000) diluted 1:750 in AD was added and incubated for 2 h at room temperature. Plates were washed four times with PBS then 50 µl BCP/NBT substrate was added to each well and allowed to develop for 5–10 min until spots were visible in the positive control wells. Reaction was stopped by rinsing the plates in DI water three times. The rubber bottom was removed and the membrane was rinsed on both sides with DI water then allowed to dry. The spots were quantitated on an ELISpot counter (AID ELISpot software v7, Autoimmun Diagnostika).

### Dendritic cell vaccine strategy in colon26 mouse tumour model

All experiments and protocols were approved by the animal welfare body at WuXi AppTec (HongKong) Limited (where experiment was performed) and the local authorities, and were conducted according to all applicable international, national and local laws and guidelines (approved by WuXi AppTec Institutional Animal Care and Use Committee). Housing conditions: temperature: 22–24 °C, 12 h day/night cycle, Humidity 40–70%. Twelve female BALB/c mice (Charles Rivers Laboratories) at 6–8 weeks of age (6–8 mice per group: control (unpulsed dendritic cells) and peptide pulsed dendritic cells [Vital River Laboratory Animal Technology Co.]) received unilateral subcutaneous injections of 3 × 10^5^ colon26 cells in PBS in a total injection volume of 100 µl/ mouse. Upon reaching individual tumour volumes of 60–80 mm^3^, mice were assigned to treatment groups based on tumour volumes aiming at comparable group mean/median tumour volumes. Within 24 h of randomisation, mice were vaccinated with 1 × 10^6^ cells/0.2 ml unpulsed or pulsed dendritic cells intravenously. To prepare the vaccine 35 BALB/c mice were humanely sacrificed by CO_2_, and two thighbones of the mice were prepared to harvest bone marrow cells. The bone marrow cells were isolated by flushing the bone cavity by sterile cold saline. All the procedures were conducted in sterile conditions and the bone marrow cells were stored at 4 °C. Next, cells were treated with GM-CSF (250 IU/ml) and IL-4 (5 IU/ml) containing medium, and incubated at 37 °C in 5% CO_2_. Medium was half changed at day 3. At day 6, cells were treated with GM-CSF, IL-4 and LPS to mature dendritic cells. After incubation for 24 h, DC cells were harvested and the phenotype was analysed by FACS (CD11c, CD80, CD86). Then, 2 × 10^5^ DC cells/ml were pulsed with peptides at 75 μg/ml (15 peptides, 5 μg/mL each) and incubated for 5 h. After harvesting and washing DC cells with medium they were ready for injection. Body weights and tumour volume [mm^3^] were performed by caliper measurement twice weekly. Termination of individual mice was conducted at day 14 of the experiment or (unilateral) tumour volume not exceeding 3000 mm^3^, in case of tumour ulceration or body mass loss at <80% of initial weight. From each group formalin-fixed samples were prepared for immuno-histochemical staining. We performed the experiment one time.

### Immuno-histochemical staining

FFPE slides were washed for 5 min with Histochoice (Sigma Aldrich), followed by two times 3 min washing in 100% Ethanol, 3 min in 70% Ethanol and 5 min in tap water. Next, samples were incubated with antigen retrieval solution (sodium citrate buffer or Tris/EDTA depending on the antibody used) at 99 °C in a water bath for 20 min. After 3× washing with purified water, samples were incubated with freshly made 6% Methanol/H_2_O_2_ for 15 min, and washed in tap water. In the next steps, slides were washed in 1% PBS-T for 5 min, blocked in blocking serum solution (Vectastatin ABC kit) for 20 min, washed again in 1% PBS-T for 5 min and incubated overnight at 4 °C with primary antibody: SDMe (13222 S, dilution 1:5000, Cell Signaling), CD8 (ab203035, dilution 1:11,000, Abcam), CD4 (ab183685, dilution 1:8000, Abcam, clone: EPR19514), CD163 (ab182422, dilution, 1:5000, Abcam, clone: EPR19518). On the next day, slides were washed with 1% PBS-T for 5 min followed by 30 min incubation with secondary antibody (Vectastain ABC kit) at room temperature. In the next step ABC solution (VECTASTAIN ABC-HRP Kit, Peroxidase, Rabbit IgG, PK-4001) was added for 30 min, slides were washed in 1% PBS-T and incubated with DAB solution (Vector DAB) for 10 min. Then, slides were washed in purified water and counterstained in haematoxylin (Sigma Aldrich). Photomicrographs were taken using a Leica microscope (at least two images from the centre and/or the margin from each sample) and results were analysed (the mean of the optical density was calculated from four mice in each group) and presented as semi-quantitative data using ImageJ v1 software (Fiji package) (National Institutes of Health).

### Statistical analysis

Statistical analyses were performed using two-tailed, unpaired Student’s *t* test when only two samples were being compared, whilst one-way ANOVA was used in experiments involving multiple comparisons (with GraphPad Prism 8 Software). Data are shown as means with SD, unless otherwise indicated. *P* values lower than 0.05 were considered significant and are labelled using asterisks (*) for *p* < 0.05, (**) for *p* < 0.01, (***) for *p* < 0.001, and (****) for *p* < 0.0001. The exact number of biological replicates is given in every figure legend.

### Reporting summary

Further information on research design is available in the Nature Portfolio Reporting Summary linked to this article.

## Supplementary information

Supplementary information

Reporting Summary

Description of Additional Supplementary Files

Supplementary data 1

Supplementary data 2

Supplementary data 3

Supplementary data 4

Supplementary data 5

Supplementary data 6

Supplementary data 7

Supplementary data 8

Supplementary data 9

Supplementary data 10

## Data Availability

Source data are provided with this paper. Additional data and materials are available from the corresponding author upon reasonable request. The RNA-seq data have been deposited in the Gene Expression Omnibus (GEO) under accession codes GSE142430 and GSE181401. Sequencing reads for colon26 tumour tissue experiments were aligned to the mm10 version of the mouse genome using GENCODE mouse lncRNA annotation version M22. Sequencing reads for HCT116 cells were aligned to GENCODE human lncRNA annotation version 34 and FANTOM5. The Immunopeptidomics data have been deposited in ProteomeXchange (PRIDE database) under accession codes PXD029613 and PXD029594. All lncRNA-derived peptide sequences were reviewed with human and mouse SwissProt protein database. UCSC Genome Browser was used for human (databases: ENCODE Regulation Txn Factr ChIP E3 Track Settings, ENCODE Regulation Txn Factor ChIP Track Settings, ENC TF Binding ENCODE 3 TFBS Track Settings, ENC TF Binding Uniform TFBS Track Settings, ENC TF Binding SYDH TFBS Track Settings) and mouse lncRNA promoter analysis (GSM288349). Functional genomics analysis, Xena browser (The TCGA TARGET GTEx database) and GEPIA v2 (http://gepia2.cancer-pku.cn/) were used. Broad Institute Cancer Cell Line Encyclopedia was used to analyse the expression of lncRNA genes in colorectal cancer cell lines. For the normal tissue and thymocyte expression analysis of murine lncRNAs giving rise to peptides, the Genevestigator tool was used. Source data are provided with this paper.

## Source data

Source data
