# Supplementary material for: Long non-coding RNA-derived peptides are immunogenic and drive a potent anti-tumour response
Source: Nat Commun. 2023 Feb 25;14:1078. doi: 10.1038/s41467-023-36826-0 (PMC9968330; doi:10.1038/s41467-023-36826-0)
Supplement: Supplementary file 1 — Supplementary information [file 41467_2023_36826_MOESM1_ESM.pdf]

Supplementary Materials and Figures

Long non-coding RNA-derived peptides are immunogenic and drive a potent anti-tumour response

Wojciech Barczak[1~], Simon M. Carr[1~], Geng Liu[1], Shonagh Munro[2], Annalisa Nicastri[3], Lian Ni Lee[4], Claire Hutchings[4], Nicola Ternette[3], Paul Klenerman[4], Alexander Kanapin[5], Anastasia Samsonova[5] and Nicholas B. La Thangue[1]*

[1]Laboratory of Cancer Biology, Department of Oncology, University of Oxford, Old Road Campus Research Building, Oxford, OX3 7DQ, United Kingdom

[2]Argonaut Therapeutics Ltd, Oxford Science Park, Robert Robinson Avenue, Oxford, OX4 4GA, United Kingdom

[3]The Jenner Institute, Nuffield Department of Medicine, University of Oxford, Oxford, OX3 7BN, United Kingdom

[4]Peter Medawar Building for Pathogen Research, University of Oxford, OX1 3SY United Kingdom

[5]Centre for Computational Biology, Peter the Great Saint Petersburg Polytechnic University, St. Petersburg, 195251, Russia

~ Each author made an equal contribution

* Corresponding author: nick.lathangue@oncology.ox.ac.uk

- Supplementary figures and figure legends S1-S11

- Supplementary data S1-S10

- Supplementary Tables S1-S2

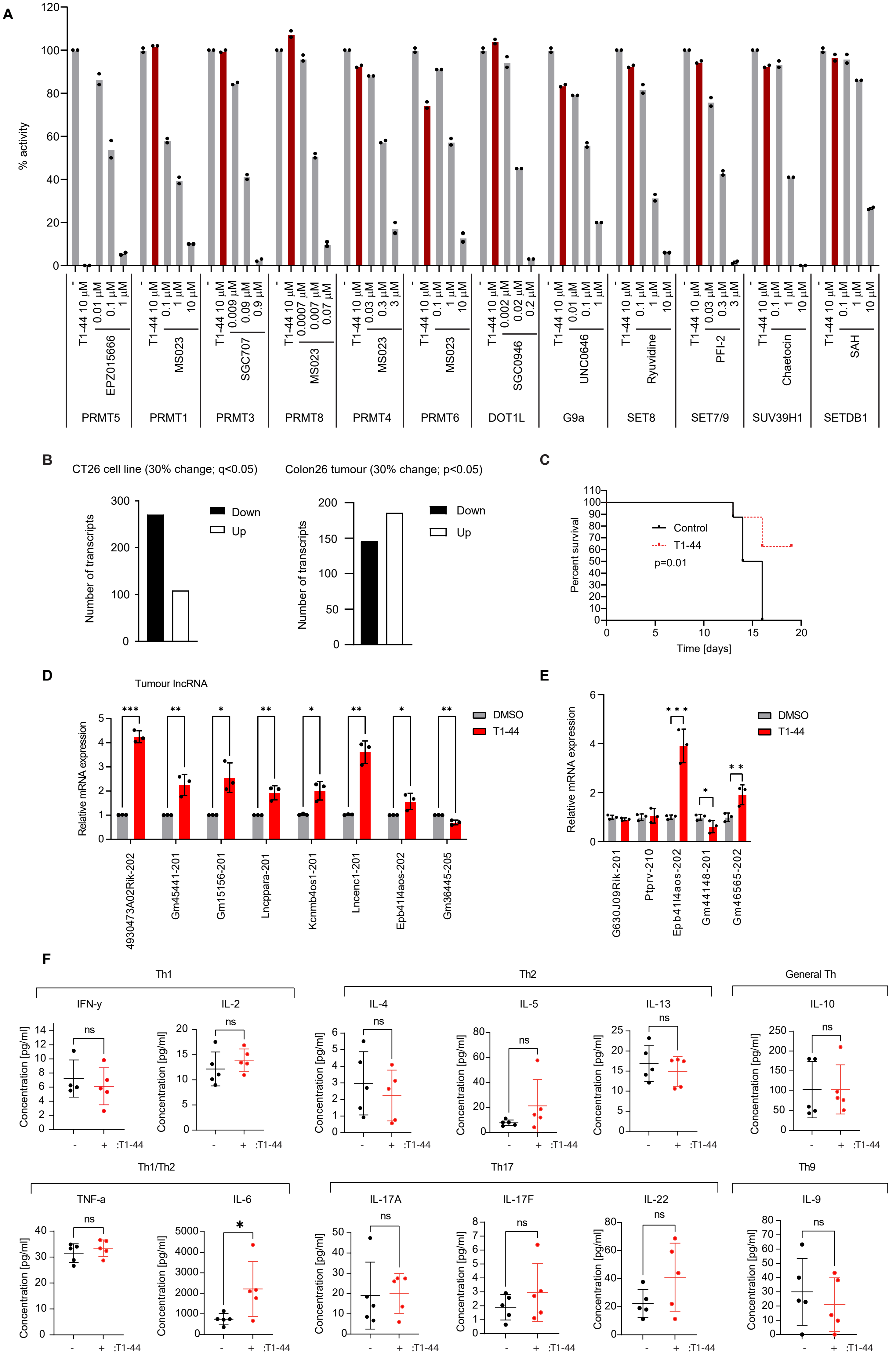
A
% activity
T1-44 10 µM
EPZ015666
MS023
SGC707
SGC0946
UNC0646
Ryuvidine
PFI-2
Chaetocin
SAH
PRMT5
PRMT1
PRMT3
PRMT8
PRMT4
PRMT6
DOT1L
G9a
SET8
SET7/9
SUV39H1
SETDB1
B
CT26 cell line (30% change; q<0.05)
Colon26 tumour (30% change; p<0.05)
Number of transcripts
Down
Up
C
Percent survival
Control
T1-44
p=0.01
Time [days]
D
Tumour lncRNA
Relative mRNA expression
DMSO
T1-44
4930473A02Rik-202
Gm45441-201
Gm15156-201
Lncppara-201
Kcnmb4os1-201
Lncenc1-201
Epb41l4aos-202
Gm36445-205
E
G630J09Rik-201
Ptprv-210
Epb41l4aos-202
Gm44148-201
Gm46565-202
F
Th1
IFN-y
IL-2
Th2
IL-4
IL-5
IL-13
General Th
IL-10
Th1/Th2
TNF-a
IL-6
Th17
IL-17A
IL-17F
IL-22
Th9
IL-9
Concentration [pg/ml]
ns
:T1-44

Supplementary Figure 1: Differential expression analysis of lncRNA transcripts in CT26 cells grown *in vitro* and *in situ* as tumours. (A) Graph showing the selectivity of compound T1-44 against a panel of 12 methyltransferases. PRMT1, PRMT3, PRMT4, PRMT5, PRMT6, PRMT8, G9a, SUV39H1, SETDB1, SET7/9, SET8 and DOT1L *in vitro* assays were performed the presence of T1-44 or a reference inhibitor for each of the enzymes. The percentage enzyme activity relative to the no compound control (-) is plotted. n = 2 biologically independent experiments; **(B)** A bar chart representation of the number of lncRNA transcripts differentially upregulated or downregulated after T1-44 treatment in the CT26 (left) or Colon26 (right) RNA-seq datasets (see supplementary Data 2), as compared to the control treatment. This analysis complements Fig. 1A **(C)** Survival curves of treated and non-treated mice from Fig. 1D; (Log-rank (Mantel-Cox); *p < 0.05), n=7 mice per group. **(D)** RNA was isolated from Colon26 tumours treated with DMSO or T1-44 as indicated, prior to RT-qPCR analysis to determine the expression of the indicated lncRNA transcripts (labelled with their ENSEMBL transcript name). n=3 biologically independent experiments (each with 3 technical replicates); results presented as mean values +/−SD; statistics were performed by two-tailed Student’s t test; * marks adjusted P value <0.05, ** marks adjusted P value <0.01, *** marks adjusted P value <0.001; **(E)** qRT-PCR analysis of the indicated lncRNAs from Colon26 tumours in T1-44 treated or untreated mice. LncRNAs were selected from targets identified in the CT26 RNA-seq. n=3 biologically independent experiments (each with 3 technical replicates); results presented as mean values +/−SD; statistics were performed by two-tailed Student’s t test; * marks adjusted P value <0.05, ** marks adjusted P value <0.01, *** marks adjusted P value <0.001; **(F)** Analysis of a panel of Th1, Th2, Th1/Th2, General Th, Th17, and Th9 cytokines in serum collected from mice treated with T1-44 or from the control group. Colon26 bearing BALB/c mice were treated with orally administrated T1-44 at 100 mg/kg for 19 days with respect to vehicle only control as described in the experiment presented in Figure 1D; serum was analysed in 5 randomly selected mice per group; results presented as mean values +/- SD; statistics were performed by two-tailed Student’s t test; * marks adjusted P value <0.05.

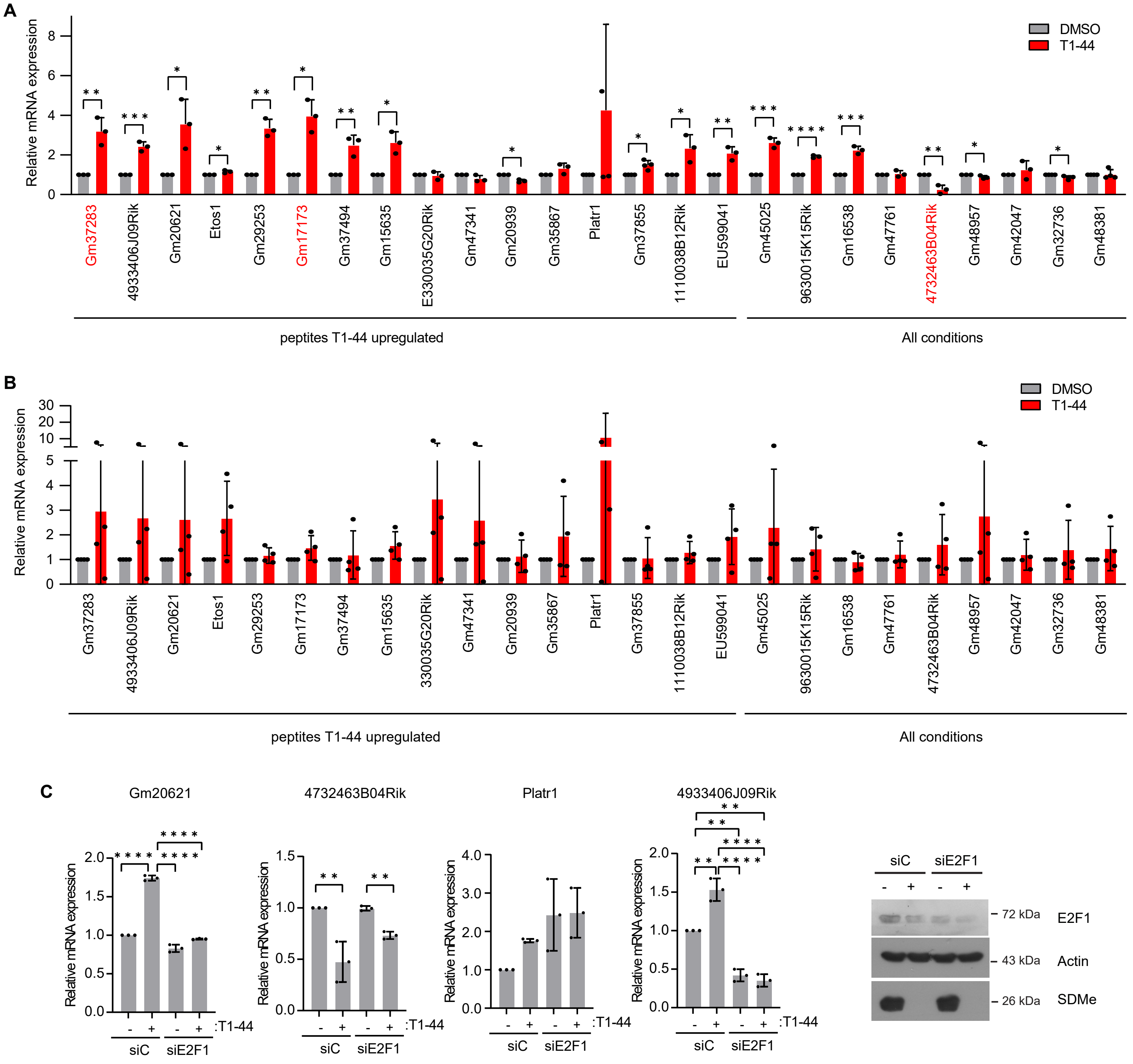
A
B
C
Relative mRNA expression
DMSO
T1-44
Gm37283
4933406J09Rik
Gm20621
Etos1
Gm29253
Gm17173
Gm37494
Gm15635
E330035G20Rik
Gm47341
Gm20939
Gm35867
Platr1
Gm37855
1110038B12Rik
EU599041
Gm45025
9630015K15Rik
Gm16538
Gm47761
4732463B04Rik
Gm48957
Gm42047
Gm32736
Gm48381
peptites T1-44 upregulated
All conditions
330035G20Rik
Gm20621
4732463B04Rik
Platr1
4933406J09Rik
:T1-44
siC
siE2F1
72 kDa
43 kDa
26 kDa
E2F1
Actin
SDMe

Supplementary Figure 2: Differential expression analysis of peptide-coding lncRNA transcripts in CT26 cells grown *in vitro* and *in situ* as tumours. (A) CT26 cells were treated for 72h with 1 µM T1-44 or DMSO as indicated, prior to qRT-PCR analysis with primers against the indicated lncRNAs. The data are expressed as relative mRNA expression as compared to DMSO treated cells. Those lncRNAs specifically giving rise to peptides bound to MHC class I were examined (those giving rise to peptides which were differentially presented after T1-44 treatment, or those observed to be unchanged in all conditions). Highlighted in red are genes described in the main text as exemplifying lncRNAs whose expression profile reflects a similar relative change in the derived peptide measured by immunopeptidomics. n=3 biologically independent experiments (each with 3 technical replicates); results presented as mean values +/−SD; statistics were performed by two-tailed Student's t test; * marks adjusted P value <0.05, ** marks adjusted P value <0.01, *** marks adjusted P value <0.001, **** marks adjusted P value <0.0001; **(B)** RNA isolated from Colon26 tumours grown in mice (see experiment presented in Fig. 1D) (4 separate mice) was used in subsequent qRT-PCR analysis with primers targeting the indicated lncRNAs (all of which give rise to peptides bound to MHC class I, as above); n=4 tumours taken from one experiment (each was analysed with 3 technical replicates); results presented as mean values +/−SD; **(C)** CT26 cells were transfected with control or E2F1 siRNA for 72h as indicated. Cells were also treated with 1 µM T1-44 8h post-transfection, and RNA was isolated from cells and a qRT-PCR experiment performed using primers against the indicated lncRNAs. These targets represent a selection of those used in supplementary Fig. 2A. An immunoblot is also included to demonstrate input E2F1 levels, and SDMe levels were used as a marker for T1-44 activity. n=3 biologically independent experiments (each with 3 technical replicates); results presented as mean values +/−SD; statistics were performed by one-way Anova with Tukey's multiple comparisons tests and * marks adjusted P value <0.05, ** marks adjusted P value <0.01, *** marks adjusted P value <0.001, **** marks adjusted P value <0.0001.

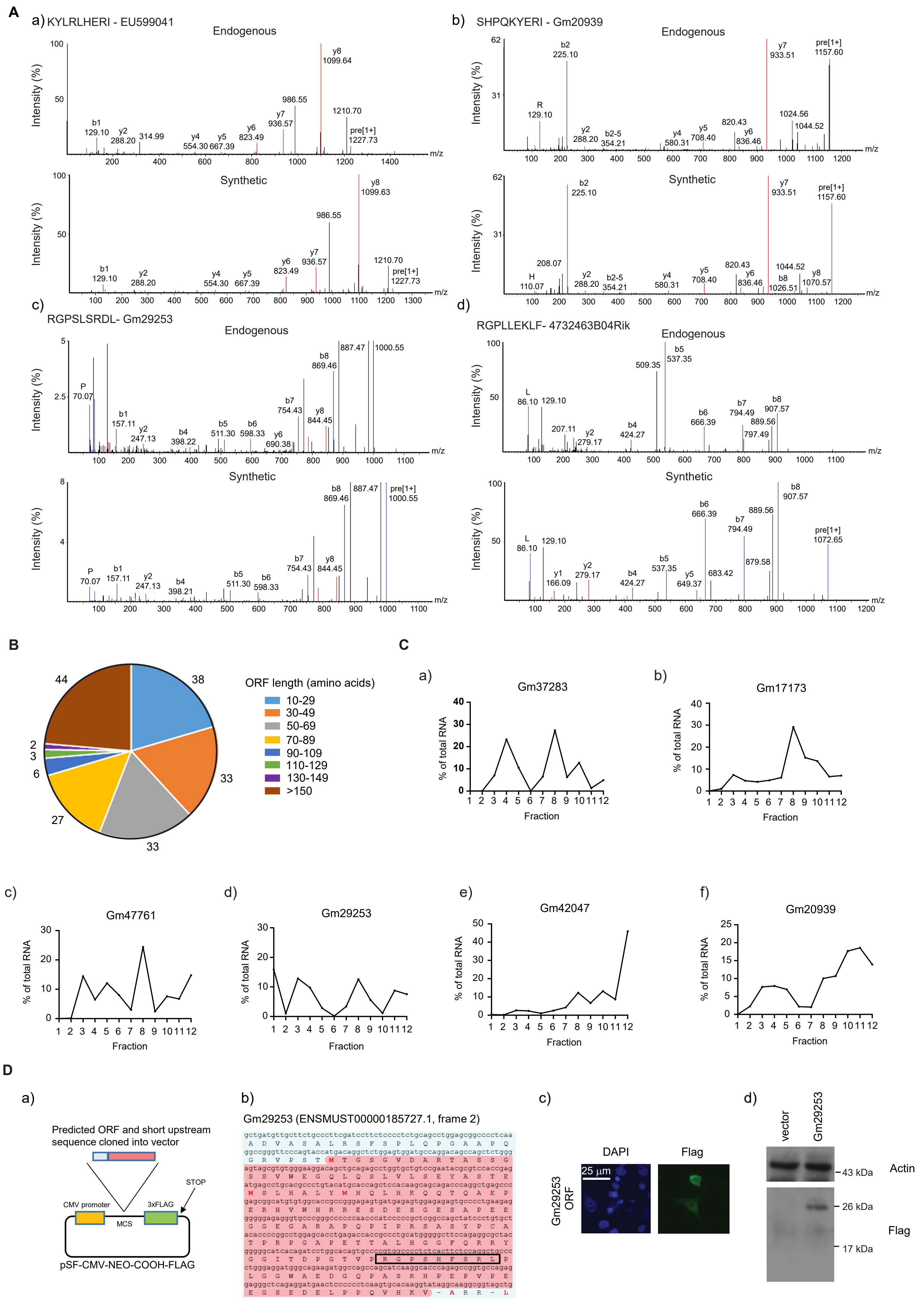
A
a) KYLRLHERI - EU599041
Endogenous
Synthetic
Intensity (%)
m/z
y8 1099.64
986.55
y7 936.57
y6 823.49
y5 667.39
y4 554.30
y2 288.20
b1 129.10
314.99
1210.70
pre[1+] 1227.73
y8 1099.63
b) SHPQKYERI - Gm20939
b2 225.10
R 129.10
y2 288.20
b2-5 354.21
y4 580.31
y5 708.40
820.43
y6 836.46
y7 933.51
1024.56
1044.52
pre[1+] 1157.60
H 110.07
208.07
b8 1026.51
y8 1070.57
c) RGPSLSRDL- Gm29253
P 70.07
b1 157.11
y2 247.13
b4 398.22
b5 511.30
b6 598.33
y6 690.38
b7 754.43
y8 844.45
b8 869.46
887.47
1000.55
b4 398.21
pre[1+] 1000.55
d) RGPLLEKLF- 4732463B04Rik
L 86.10
129.10
207.11
y2 279.17
b4 424.27
509.35
b5 537.35
b6 666.39
b7 794.49
797.49
889.56
b8 907.57
y1 166.09
y5 649.37
683.42
879.58
pre[1+] 1072.65
B
ORF length (amino acids)
10-29
30-49
50-69
70-89
90-109
110-129
130-149
>150
44
38
33
33
27
6
3
2
C
a) Gm37283
b) Gm17173
c) Gm47761
d) Gm29253
e) Gm42047
f) Gm20939
% of total RNA
Fraction
D
a)
Predicted ORF and short upstream sequence cloned into vector
CMV promoter
MCS
3xFLAG
STOP
pSF-CMV-NEO-COOH-FLAG
b)
Gm29253 (ENSMUST00000185727.1, frame 2)
c)
DAPI
Flag
25 µm
Gm29253 ORF
d)
vector
Gm29253
Actin
Flag
43 kDa
26 kDa
17 kDa

Supplementary Figure 3. Characterisation of the lncRNA transcripts giving rise to peptides identified in the CT26 immunopeptidomics. (A) Synthetic peptides corresponding to MHC class I bound peptides identified from the CT26 immunopeptidomics analysis were synthesised and spectra were analysed by mass spectrometry to confirm the identifications made. Examples are shown of the peptide-encoding lncRNAs *EU599041* (a), *Gm20939* (b), *Gm29253* (c), and *4732463B04Rik* (d). The top mass spectrometry profile represents the peptide identification from the endogenous CT26 experiment, whilst the lower spectra represents the ion profile from the synthetic peptide. **(B)** LncRNAs giving rise to peptides identified in the immunopeptidomics analysis were translated in all 3 frames to aid identification of potential open reading frames (ORFs) containing the peptide sequence. Where a predicted ORF could be identified, its length (in amino acids) was scored and the data plotted in a pie chart. ORF lengths were binned into groupings as indicated. **(C)** Polysome profiling for lncRNAs giving rise to MHC class I peptides: *Gm37283* (a), *Gm17173* (b), *Gm47761* (c), *Gm29253* (d), *Gm42047* (e), and *Gm20939* (f). Data are presented as percentage of total RNA in each fraction; n=3 biologically independent experiments (each with 3 technical replicates); results presented as mean values; **(D)** (a) A schematic representation of the pSF-CMV-NEO-COOH-FLAG plasmid that was used as the cloning vector for insertion of predicted ORFs from mouse lncRNA transcripts found to encode peptides presented on MHC class I. The predicted ORF and a short section of upstream sequence (containing any endogenous ribosome binding site) was ligated into the multiple cloning site (MCS) of the vector, in frame with the C-terminal 3xFLAG tag. Note that a ribosome binding site is not provided in the vector itself. (b) Part of the sequence of the *Gm29253* lncRNA transcript is displayed, with the predicted ORF (shown in red) giving rise to the identified MHC class I bound peptide (boxed in black). This ORF and a short section of upstream sequence was cloned into the pSF-CMV-NEO-COOH-3xFLAG vector. (c) CT26 cells were transfected with the *Gm29253* ORF-Flag plasmid for 48 h prior to immune fluorescence analysis with anti-Flag antibodies. Cell nuclei were stained with DAPI. n=2 biologically independent experiments (d) CT26 cells were transfected with *Gm29253* ORF-Flag plasmid prior to immunoblot analysis with Flag antibodies. n=3 biologically independent experiments.

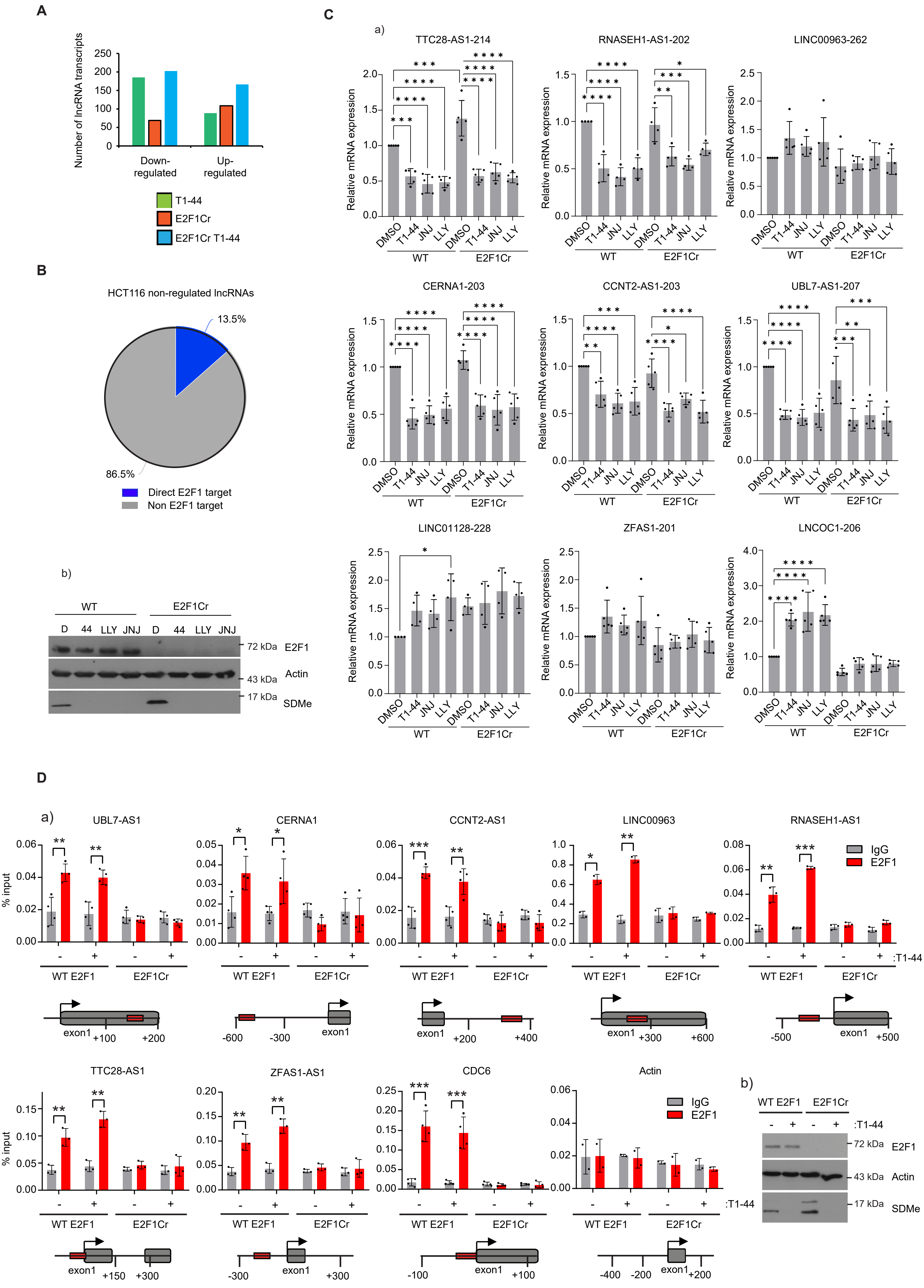

A
Number of lncRNA transcripts
Down-regulated
Up-regulated
T1-44
E2F1Cr
E2F1Cr T1-44
B
HCT116 non-regulated lncRNAs
13.5%
86.5%
Direct E2F1 target
Non E2F1 target
b)
WT
E2F1Cr
D
44
LLY
JNJ
72 kDa
E2F1
43 kDa
Actin
17 kDa
SDMe
C
a)
TTC28-AS1-214
RNASEH1-AS1-202
LINC00963-262
CERNA1-203
CCNT2-AS1-203
UBL7-AS1-207
LINC01128-228
ZFAS1-201
LNCOC1-206
Relative mRNA expression
DMSO
T1-44
JNJ
LLY
D
a)
UBL7-AS1
CERNA1
CCNT2-AS1
LINC00963
RNASEH1-AS1
TTC28-AS1
ZFAS1-AS1
CDC6
Actin
% input
IgG
E2F1
WT E2F1
E2F1Cr
:T1-44
exon1
b)

Supplementary Figure 4. Differential expression analysis of lncRNA transcripts in HCT116 cells. (A) A bar chart to represent the total number of lncRNA transcripts that are differentially up-and down-regulated at a statistically significant level ($q < 0.05$) in each cell line and treatment, with respect to WT E2F1 DMSO treated cells. This analysis complements Fig. 3A **(B)** The percentage of non-regulated lncRNA genes that score as potential direct E2F1 target genes (using ChIP-seq data from ENCODE, reads within 500 bp of the TSS) from the HCT116 RNA-seq analysis. **(C)** (a) WT E2F1 or E2F1 Cr cell lines were treated with 1 µM T1-44, 100 nM JNJ64619178 (JNJ), or 1 µM LLY-283 (LLY) for 48 h prior to RT-qPCR analysis to determine the expression of the indicated lncRNA transcripts (labelled with their ENSEMBL transcript name). n=5 biologically independent experiments (each with 3 technical replicates); results presented as mean values +/−SD; statistics were performed by one-way Anova with Tukey's multiple comparisons tests and * marks adjusted P value <0.05, ** marks adjusted P value <0.01, *** marks adjusted P value <0.001, **** marks adjusted P value <0.0001. (b) An immunoblot is also included to display input protein levels of E2F1, and SDMe was used as a marker for PRMT5 inhibitor activity. **(D)** (a) ChIP analysis of T1-44 treated WT E2F1 or E2F1 Cr cell lines. ChIP-seq data from ENCODE was used to identify potential E2F1 binding sites (marked by a red box), and primers around these sites were used in the qPCR. (b) An immunoblot to display input protein. n=3 biologically independent experiments (each with 3 technical replicates); results presented as mean values +/−SD; statistics were performed by two-tailed Student's t test; * marks adjusted P value <0.05, ** marks adjusted P value <0.01, *** marks adjusted P value <0.001.

LNCOC1

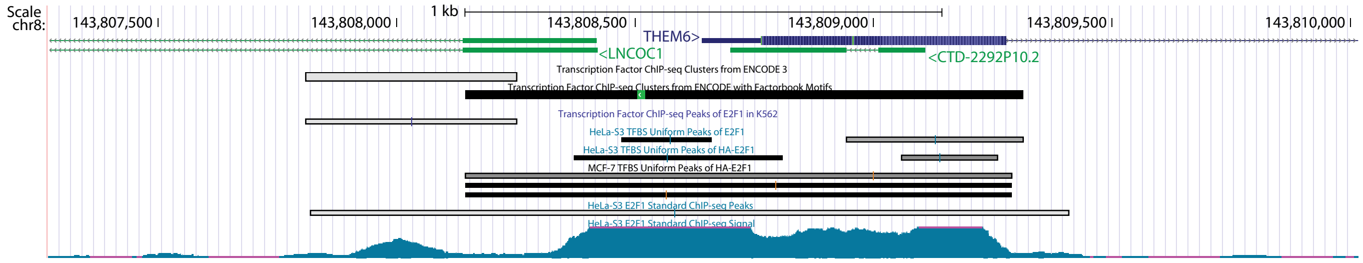

LINC01128

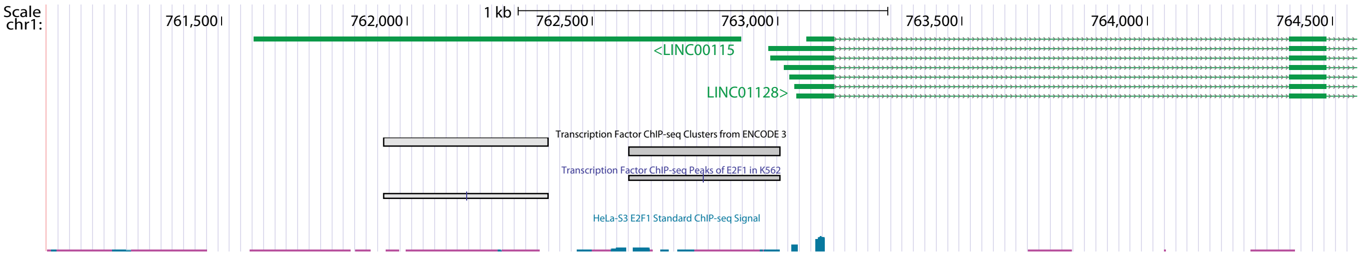

CERNA1

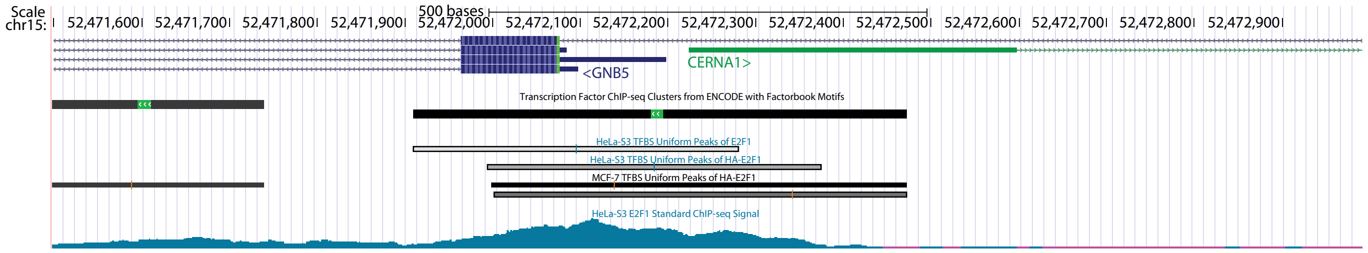

CCNT2-AS1

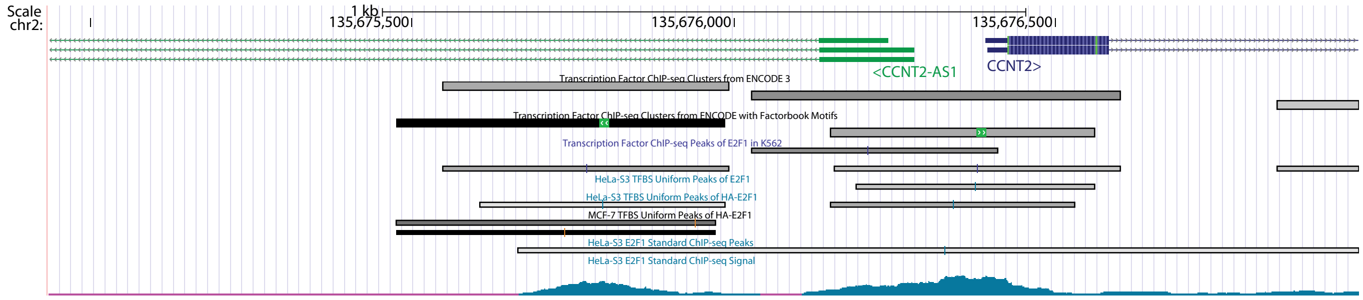

UBL7-AS1

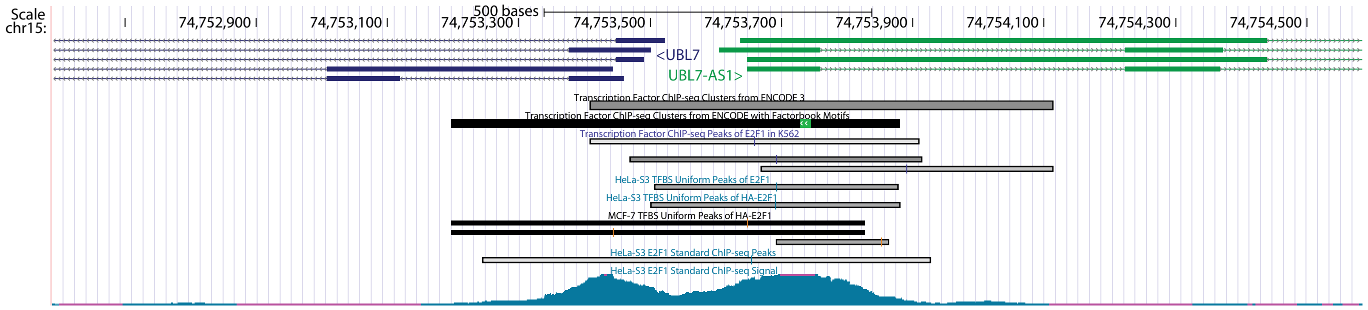

LINC00963

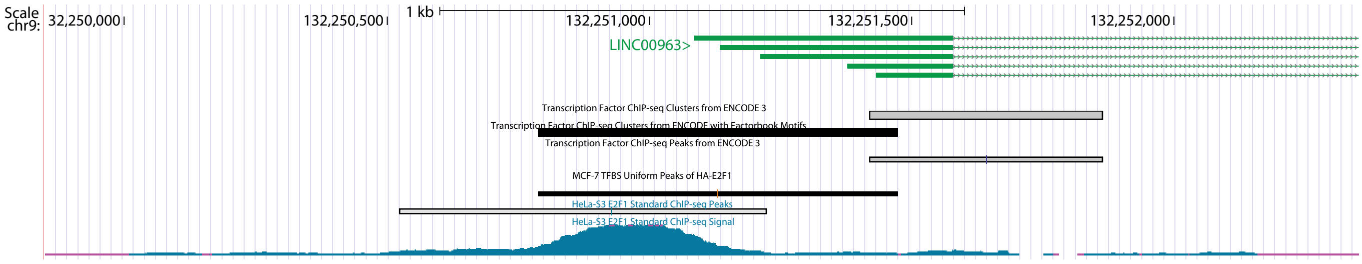

RNASEH1-AS1

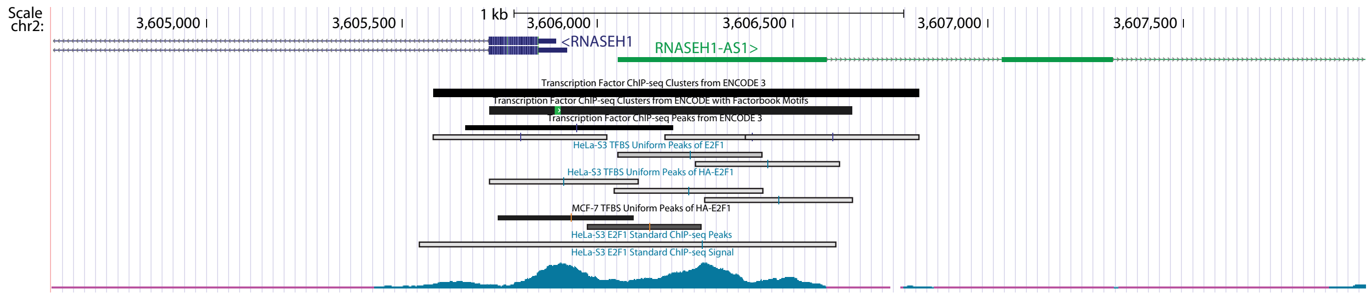

TTC28-AS1

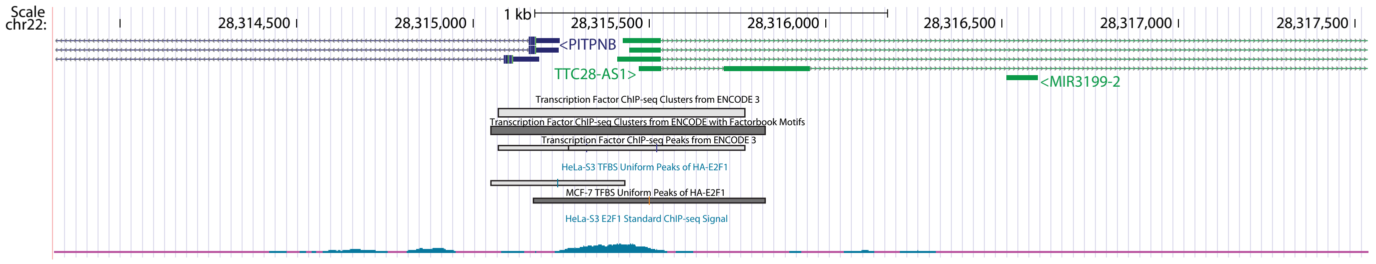

ZFAS1

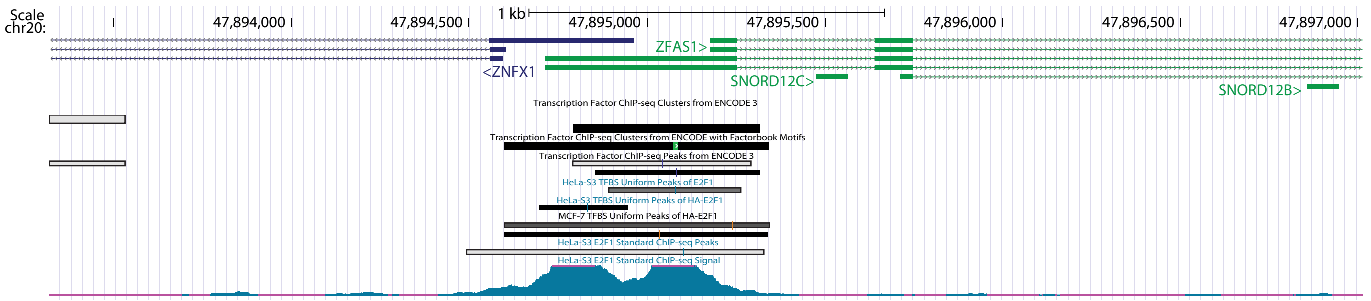

Supplementary Figure 5. Genome browser profiles for a selection of lncRNA genes identified as being differentially expressed in the HCT116 RNA-seq. Genome browser profiles for a selection of lncRNA genes identified as being differentially expressed in the HCT116 RNA-seq analysis (and examined by RT-qPCR in Fig. 3C). Gene structure is displayed at the top of the figures, with green rectangles and lines representing exons and introns of lncRNA transcripts, and blue rectangles and lines representing exons and introns of neighbouring protein coding genes. The positions of E2F1 ChIP-seq clusters and peaks derived from several datasets in ENCODE are displayed as shaded rectangles in the central part of the figure. Each rectangle encloses a peak cluster of E2F1 occupancy, with the darkness of the box being proportional to the level of enrichment observed in any cell type contributing to the cluster. At the bottom of each figure is a blue graph displaying enrichment for E2F1 binding (standard ChIP-seq signal). Examples for *LNCOC1*, *LINC01128*, *CERNA1*, *CCNT2- AS1*, *UBL7-AS1*, *LINC00963*, *RNASEH1-AS1*, *TTC28-AS1* and *ZFAS1* are displayed.

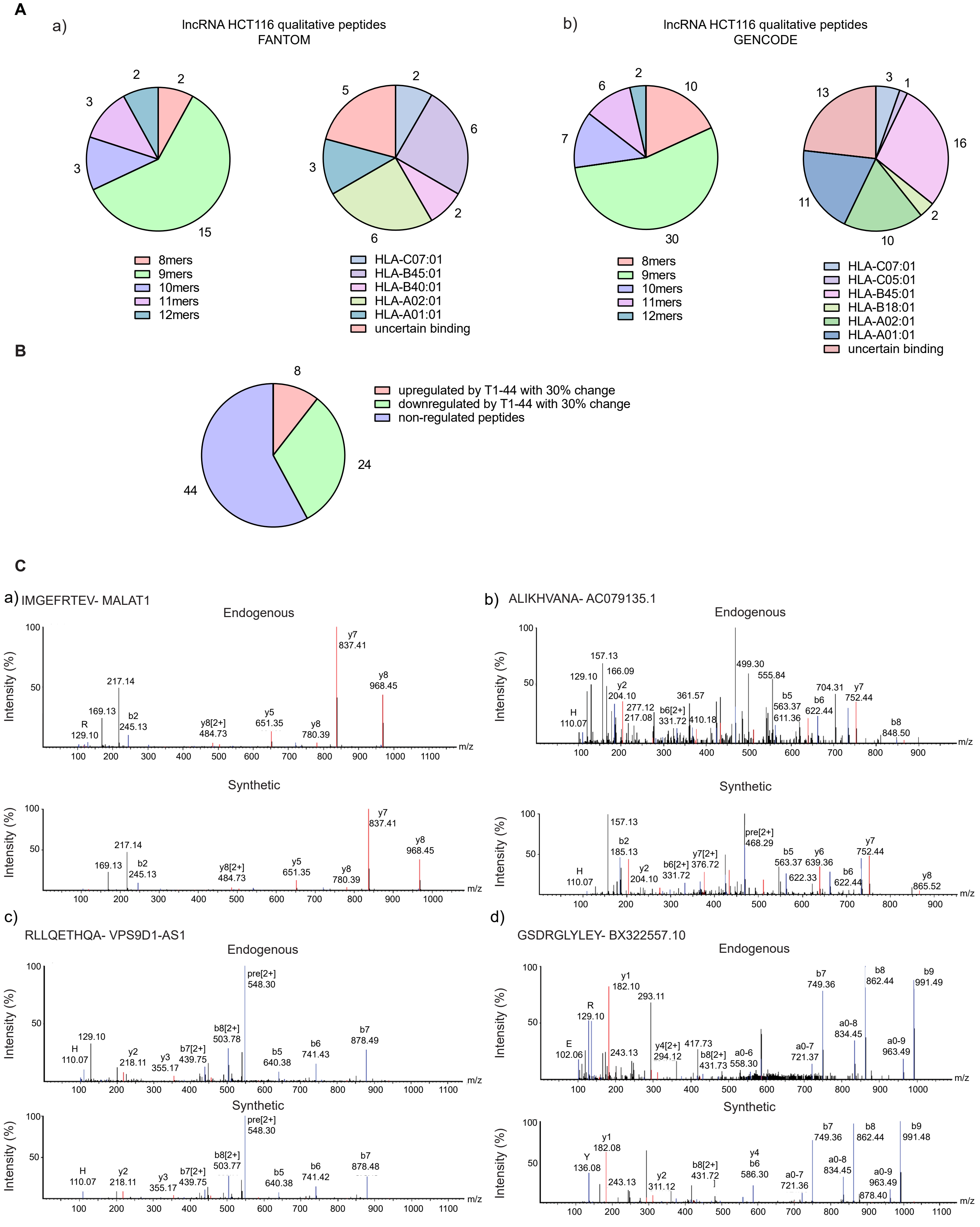
A
a) lncRNA HCT116 qualitative peptides FANTOM
8mers
9mers
10mers
11mers
12mers
HLA-C07:01
HLA-B45:01
HLA-B40:01
HLA-A02:01
HLA-A01:01
uncertain binding
b) lncRNA HCT116 qualitative peptides GENCODE
8mers
9mers
10mers
11mers
12mers
HLA-C07:01
HLA-C05:01
HLA-B45:01
HLA-B18:01
HLA-A02:01
HLA-A01:01
uncertain binding
B
upregulated by T1-44 with 30% change
downregulated by T1-44 with 30% change
non-regulated peptides
C
a) IMGEFRTEV- MALAT1
b) ALIKHVANA- AC079135.1
c) RLLQETHQA- VPS9D1-AS1
d) GSDRGLYLEY- BX322557.10
Endogenous
Synthetic
Intensity (%)
m/z

Supplementary Figure 6. Immunopeptidomic analysis of lncRNA-derived peptides in HCT116 cells. (A) An immunopeptidomics analysis was performed on HCT116 cells, and MHC class I bound peptides were compared against an lncRNA database generated using FANTOM (a) or GENCODE (b) annotation. Displayed on the left are the total numbers of peptides (pooled from two independent experiments) derived from lncRNAs, separated by their size from 8-mers to 12-mers. The predicted MHC allele binding preference for each lncRNA derived peptide calculated using NetMHCpan4.1 software are displayed on the right. The data reported here was derived from an immunopeptidomic experiment performed on two biologically independent replicates. **(B)** The total number of lncRNA derived peptides (pooled from two independent experiments) that were differentially presented or non-regulated after T1-44 treatment (quantitative analysis performed on the GENCODE lncRNA database). The data reported here was derived from an immunopeptidomic experiment performed on two biologically independent replicates **(C)** Synthetic peptides corresponding to MHC class I bound peptides identified from the HCT116 immunopeptidomics analysis were synthesised and analysed by mass spectrometry to confirm the identifications made. Examples are shown of the peptide-encoding lncRNAs, *MALAT1* (a), *AC079135.1* (b), *VPS9D1-AS1* (c), and *BX322557* (d). The top mass spectrometry profile represents the peptide identification from the endogenous CT26 experiment, whilst the lower spectra represents the ion profile from the synthetic peptide.

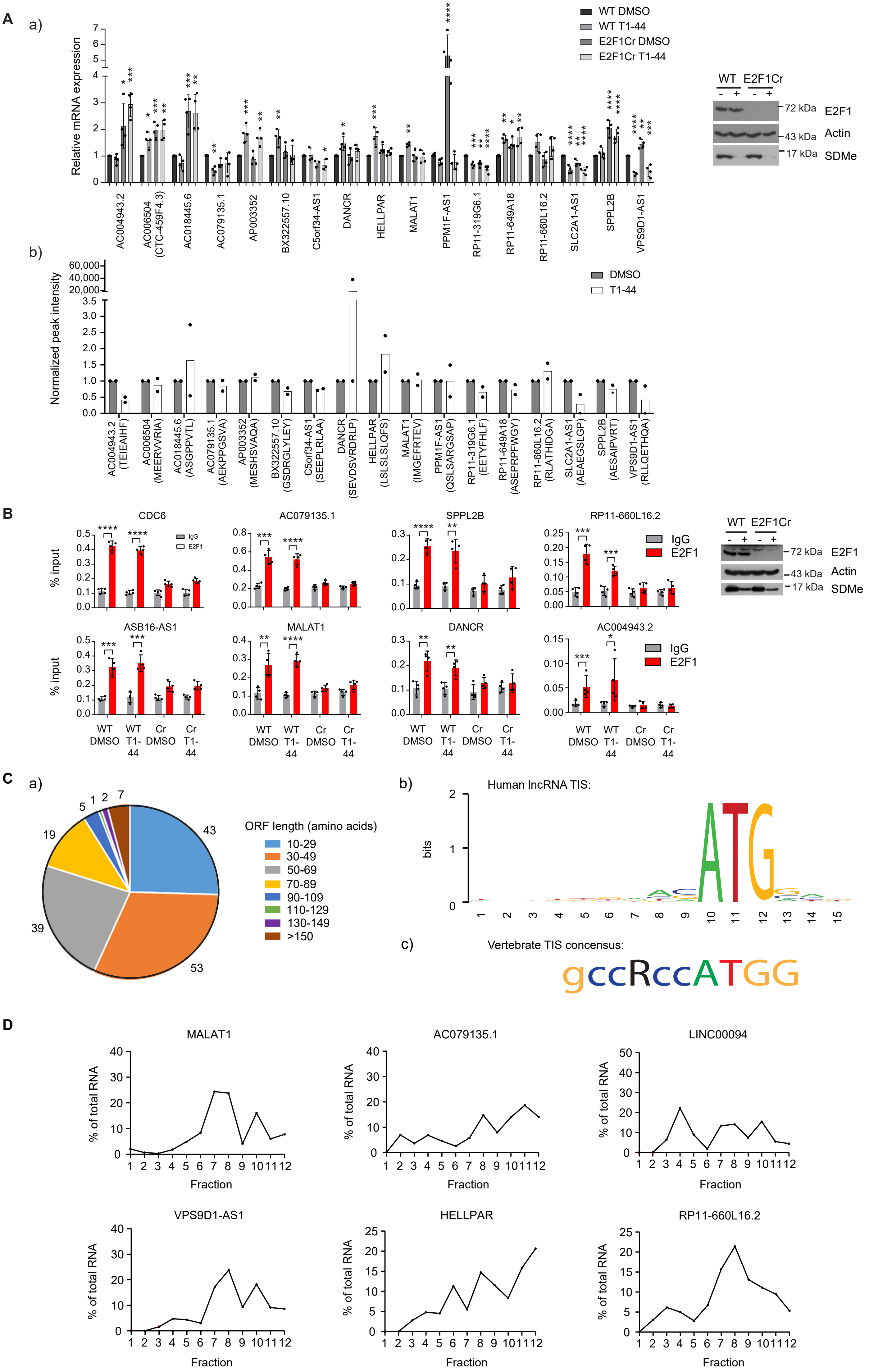

A
a)
Relative mRNA expression
WT DMSO
WT T1-44
E2F1Cr DMSO
E2F1Cr T1-44
AC004943.2
AC006504 (CTC-459F4.3)
AC018445.6
AC079135.1
AP003352
BX322557.10
C5orf34-AS1
DANCR
HELLPAR
MALAT1
PPM1F-AS1
RP11-319G6.1
RP11-649A18
RP11-660L16.2
SLC2A1-AS1
SPPL2B
VPS9D1-AS1
WT E2F1Cr
- + - +
72 kDa E2F1
43 kDa Actin
17 kDa SDMe
b)
Normalized peak intensity
DMSO
T1-44
AC004943.2 (TEIEAIHF)
AC006504 (MEERVVRIA)
AC018445.6 (ASGPPVTL)
AC079135.1 (AEKPPGSVA)
AP003352 (MESHSVAQA)
BX322557.10 (GSDRGLYLEY)
C5orf34-AS1 (SEEPLRLAA)
DANCR (SEVDSVRDRLP)
HELLPAR (LSLSLSLQFS)
MALAT1 (IMGEFRTEV)
PPM1F-AS1 (QSLSARGSAP)
RP11-319G6.1 (EETYFHLF)
RP11-649A18 (ASEPRPFWGY)
RP11-660L16.2 (RLATHIDGA)
SLC2A1-AS1 (AEAEGSLGP)
SPPL2B (AESAIPVRT)
VPS9D1-AS1 (RLLQETHQA)
B
CDC6
AC079135.1
SPPL2B
RP11-660L16.2
ASB16-AS1
MALAT1
DANCR
AC004943.2
% input
IgG
E2F1
WT DMSO
WT T1-44
Cr DMSO
Cr T1-44
WT E2F1Cr
72 kDa E2F1
43 kDa Actin
17 kDa SDMe
C
a)
ORF length (amino acids)
10-29
30-49
50-69
70-89
90-109
110-129
130-149
>150
43
53
39
19
5
1
2
7
b)
Human lncRNA TIS:
bits
c)
Vertebrate TIS concensus:
gccRccATGG
D
MALAT1
AC079135.1
LINC00094
VPS9D1-AS1
HELLPAR
RP11-660L16.2
% of total RNA
Fraction

Supplementary Figure 7. Characterisation of the lncRNA transcripts giving rise to peptides identified in the HCT116 immunopeptidomics. (A) a) WT E2F1 HCT116 and E2F1Cr cells were treated for 48 h with 1 µM T1-44 or DMSO as indicated. RNA was isolated and used in qRT-PCR analysis with primers against the indicated lncRNAs (all of which encode peptides presented on MHC class I). Results are expressed as fold change in expression as compared to the WT E2F1 HCT116 cells treated with DMSO. Stars represent statistical significance as calculated by two-tailed Student’s t-test comparing the indicated treatment to the corresponding WT DMSO control sample. An immunoblot is included to demonstrate input levels of E2F1, and SDMe is used as a control for T1-44 activity; n=4 biologically independent experiments (each with 3 technical triplicates); results presented as mean values +/−SD; statistics were performed by one-way Anova with Tukey’s multiple comparisons tests and * marks adjusted P value <0.05, ** marks adjusted P value <0.01, *** marks adjusted P value <0.001, **** marks adjusted P value <0.0001; (b) Quantitation of peptide abundance from the immunopeptidomics analysis derived from selected lncRNAs is presented on the graph as a normalised peak intensity (please see supplementary Data 5-7). n=2 biologically independent experiments (each performed in technical duplicate). **(B)** WT E2F1 HCT116 and E2F1Cr cells were treated for 48h with 1 µM T1-44 or DMSO as indicated. Chromatin was then extracted and immunoprecipitated with control IgG or antibodies against E2F1. The percentage enrichment of chromatin around the promoters of the indicated lncRNA genes as compared to input samples is presented. These targets represent a selection of those lncRNAs analysed in supplementary Fig. 7A. An immunoblot is also included to demonstrate input levels of E2F1, and SDMe is used as a control for T1-44 activity. n=4 biologically independent experiments (each with 3 technical replicates); results presented as mean values +/−SD; statistics were performed by two-tailed Student’s t test; * marks adjusted P value <0.05, ** marks adjusted P value <0.01, *** marks adjusted P value <0.001, **** marks adjusted P value <0.0001; **(C)** (a) LncRNAs giving rise to peptides identified in the immunopeptidomics analysis were translated in all 3 frames to aid identification of potential open reading frames (ORFs) containing the peptide sequence. Where a predicted ORF could be identified, its length (in amino

acids) was scored and the data plotted on a pie chart. ORF lengths were binned into groupings as indicated. (b) A sequence logo demonstrating the amino acid conservation around the translation initiation sequence of potential ORFs identified from human lncRNAs (Human lncRNA TIS) giving rise to MHC class I associated peptides. (c) The consensus vertebrate TIS is displayed. Upper case letters indicate highly conserved bases, whilst a lower case letter denotes the most common base at that position where the base can nevertheless vary. ‘R’ indicates that a purine (adenine or guanine) is always observed at this position. **(D)** Polysome profile assays for lncRNAs giving rise to MHC class I peptides: *MALAT1* (a), *AC079135.1* (b), *LINC00094* (c), *VPS9D1-AS1* (d), *HELLPAR*, (e) and *RP11-660L16.2* (f). Data are presented as percentage of total RNA in each fraction; n=3 biologically independent experiments (each with 3 technical replicates); results presented as mean values.

A

Sequence	Accession	Gene name	Length	NetMHCpan score	NetMHCpan allele	Fold change (T1-44/DMSO)	Expression in CT26	Expression in thymus
RGPSHFSRL	ENSMUST00000185727.1	Gm29253	9	0.0022	H-2-Dd	Infinity	high	low
KYLRLHERI	ENSMUST00000205748.1	EU599041	9	0.0136	H-2-Kd	1.91	low	below cutoff
RGPLLEKLF	ENSMUST00000180751.1	4732463B04Rik	9	0.0091	H-2-Dd	1.05	medium	below cutoff
SLPVRSLSL	ENSMUST00000219716.1	Gm47341	9	0.1018	H-2-Dd	1.01	low	below cutoff
TGPRRPQI	ENSMUST00000176338.1<br>ENSMUST00000177166.1<br>ENSMUST00000176618.1	Gm20621	8	0.0174	H-2-Dd	0.87	medium/low	below cutoff
SHPQKYERI	ENSMUST00000108007.4<br>ENSMUST00000234295.1	Gm20939	9	0.0931	H-2-Dd	1.43	high/medium	low
RPLISIKHGL	ENSMUST00000222952.1	Gm48381	10	0.2216	H-2-Ld	-	high/medium	low/ below cutoff
SNISYKNGI	ENSMUST00000188474.1	Platr1	9	1.7108	H-2-Dd	1.08	medium	below cutoff
GFLGSNTDI	ENSMUST00000228099.1<br>ENSMUST00000227204.1	Gm48957	9	0.5172	H-2-Kd	0.77	low	below cutoff
LNPSALSAL	ENSMUST00000200427.1	Gm32736	9	0.156	H-2-Dd	1.09	low	low
RATPEVTLI	ENSMUST00000214496.1	Gm38575	9	0.2693	H-2-Qa1	0.98	low	below cutoff
GSPSLVHQV	ENSMUST00000225122.1	9630015K15Rik	9	0.1296	H-2-Dd	0.88	low	low
YNPILSKL	ENSMUST00000207061.1	A230057D06Rik	8	0.0753	H-2-Dd	0.95	low	below cutoff
YYIPGLKGI	ENSMUST00000219259.1<br>ENSMUST00000217920.1	Gm47761	9	0.0079	H-2-Kd	1.18	high/medium	high
WPQDGTFNL	ENSMUST00000199958.1	Gm43305	9	0.0918	H-2-Ld	0.95	high/medium	high
RGPSLSRDL	ENSMUST00000185727.1	Gm29253	9	0.0141	H-2-Dd	1.42	high	low
MGPPRAAGI	ENSMUST00000173811.7<br>ENSMUST00000174039.7	1110038B12Rik	9	0.0193	H-2-Dd	0.99	high	high
SYLQLHENF	ENSMUST00000188038.2	Gm37494	9	0.0684	H-2-Kd	1.07	high	high
HPTVPNPYNLL	ENSMUST00000219259.1	Gm47761	11	0.6056	H-2-Ld	1.05	high/medium	high
REGPVRGLSI	ENSMUST00000185494.1	Gm28653	10	0.0813	H-2-Dd	1.01	high	below cutoff

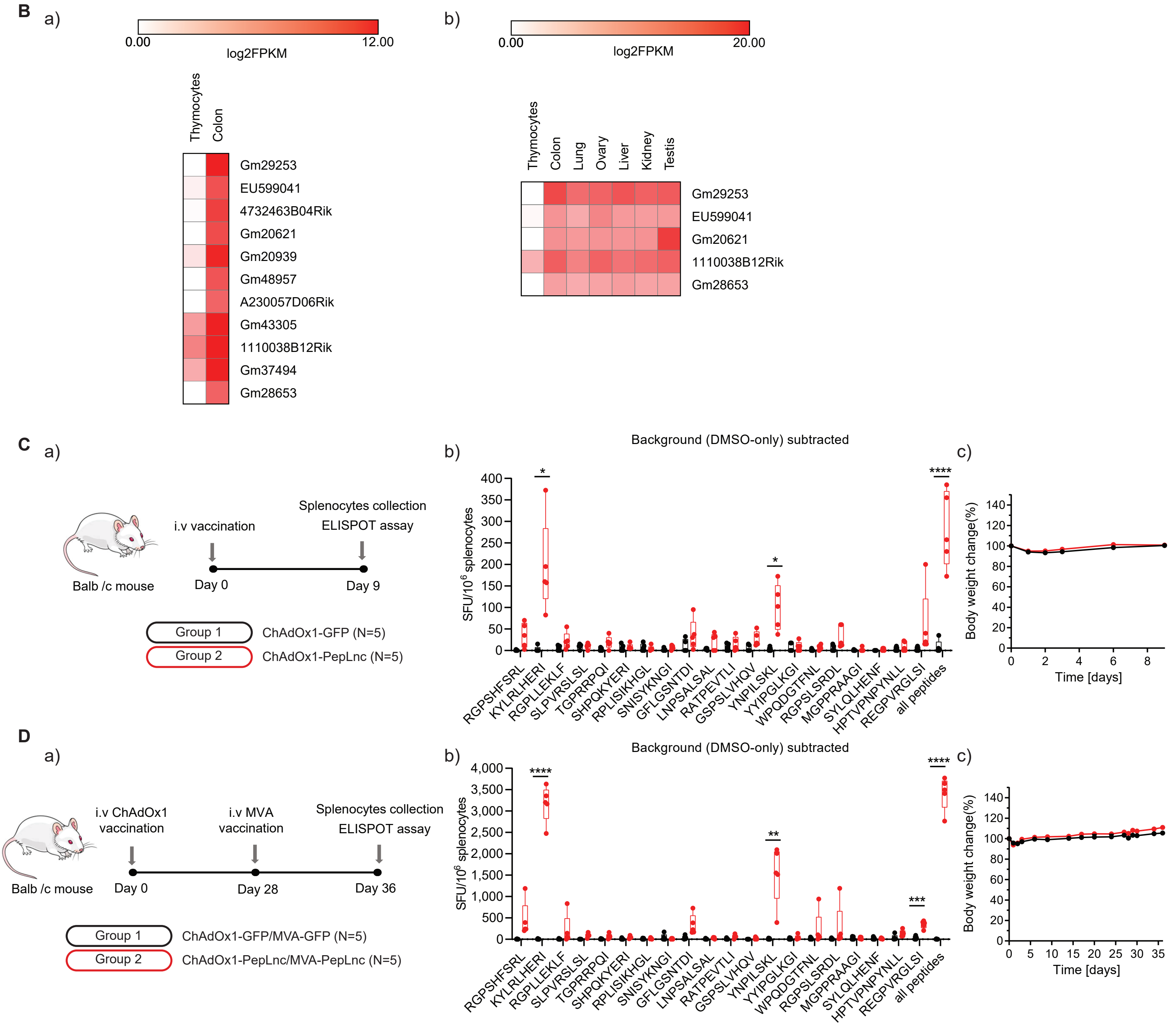

Supplementary Figure 8. Characterisation of the 20 selected peptides encoded by murine lncRNAs. (A) Characterisation of the 20 selected peptides encoded by murine lncRNAs identified in the immunopeptidomics experiment on CT26 cells treated with T1-44, with respect to DMSO control. These peptides were used to generate the poly-antigen cassette expressed from the ChAdOx1 and MVA viral vectors. In order from the left, columns characterise: sequence of the peptide; transcript accession ID; lncRNA gene name; peptide length; Net MHCpan score and allele columns show the results from binding affinity prediction analysis; Peptide abundance fold change (T1-44 treated vs DMSO treated) (derived using PROGENESIS software); expression level in CT26 cells (based on our in house RNA-seq and other databases – GENEVESTIGATOR software) (Low - log2TPM11.5); expression in thymus (based on EXPRESSION ATLAS - www.ebi.ac.uk/gxa/home and GENEVESTIGATOR software)(Low - log2TPM11.5; below cut-off - no expression). **(B)** Expression of murine lncRNAs giving rise to peptides in thymocytes and normal tissue using two different datasets; (a) The heatmap represents lncRNA expression levels in thymocytes (GEO: GSE79174 (subseries of GSE79179)) and normal mouse colon tissue (GEO: GSE71632 and GSE63299); (b) The heatmap represents lncRNA expression levels in thymocytes (GEO: GSE79174 (subseries of GSE79179) and several normal mouse tissues (GEO GSE9954). Expression presented as log2(fpkm). All data were collected using Genevestigator software. **(C)** (a) Groups of 5 BALB/c mice were vaccinated with ChAdOx1-PepLnc adenoviral vectors expressing a poly-antigen cassette containing the selected 20 lncRNA-derived peptides (in red), or a control ChAdOx1-GFP adenoviral vector (in black). At 9 days post vaccination, the mice were sacrificed and their splenocytes collected for ELIspot assay. (b) Splenocytes from each mouse were stimulated with the indicated individual peptides, or a pool of all 20, and activity was measured in interferon gamma-based ELIspot assay; n=5 mice (multiple comparison two-tailed Student's t test; * marks adjusted P value <0.05, **** marks adjusted P value <0.0001), box and whiskers are defined as minimum, first quartile, median, third quartile, and maximum of data, (c) Relative body weight of BALB/c mice presented as a mean value +/- SEM, n=5 mice from one experiment. These data complement the experiment presented in Fig. 5A. **(D)** (a) Groups of 5 BALB/c mice were vaccinated with ChAdOx1-PepLnc or ChAdOx1-GFP as detailed above. 28 days later, the mice received a booster vaccination with MVA-PepLnc (in red) or MVA-GFP (in black). At 9 days post-booster the mice were

sacrificed and their splenocytes collected for ELIspot assay. (b) Splenocytes from each mouse were stimulated with the indicated individual peptide, or a pool of all 20, and activity was measured in interferon gamma-based ELIspot assay. n=5 mice, (multiple comparison two-tailed Student’s t test; ** marks adjusted P value <0.01, *** marks adjusted P value <0.001, **** marks adjusted P value <0.0001), box and whiskers are defined as minimum, first quartile, median, third quartile, and maximum of data (c) Relative body weight of BALB/c mice presented as a mean value +/- SEM, n=5 mice from one experiment. These data complement the experiment presented in Fig. 5A.

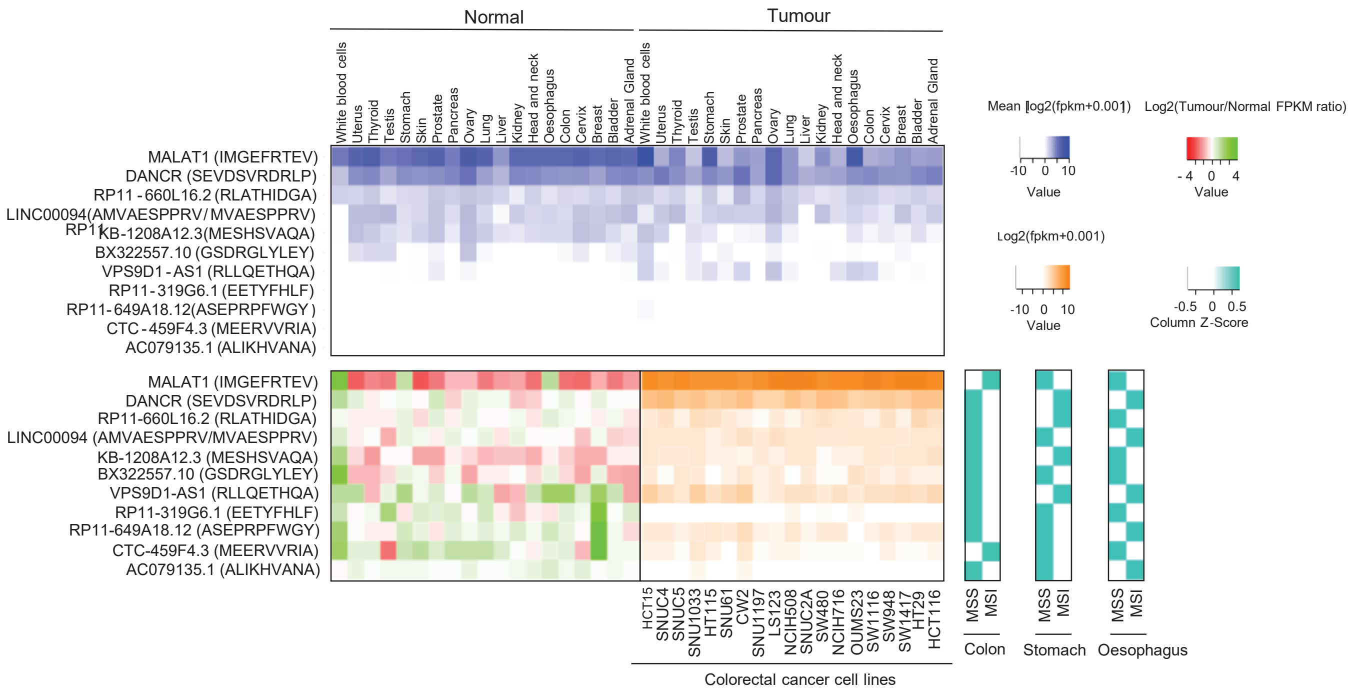

Normal
Tumour
White blood cells
Uterus
Thyroid
Testis
Stomach
Skin
Prostate
Pancreas
Ovary
Lung
Liver
Kidney
Head and neck
Oesophagus
Colon
Cervix
Breast
Bladder
Adrenal Gland
MALAT1 (IMGEFRTEV)
DANCR (SEVDSVRDRLP)
RP11-660L16.2 (RLATHIDGA)
LINC00094(AMVAESPPRV/MVAESPPRV)
KB-1208A12.3(MESHSVAQA)
BX322557.10 (GSDRGLYLEY)
VPS9D1-AS1 (RLLQETHQA)
RP11-319G6.1 (EETYFHLF)
RP11-649A18.12(ASEPRPFWGY)
CTC-459F4.3 (MEERVVRIA)
AC079135.1 (ALIKHVANA)
Mean log2(fpkm+0.001)
-10 0 10
Value
Log2(Tumour/Normal FPKM ratio)
-4 0 4
Value
Log2(fpkm+0.001)
-10 0 10
Value
-0.5 0 0.5
Column Z-Score
HCT15
SNUC4
SNUC5
SNU1033
HT115
SNU61
CW2
SNU1197
LS123
NCIH508
SNUC2A
SW480
NCIH716
OUMS23
SW1116
SW948
SW1417
HT29
HCT116
Colorectal cancer cell lines
MSS
MSI
Colon
Stomach
Oesophagus

Supplementary Figure 9. Comparison of human lncRNA transcripts giving rise to peptides in tumour versus normal tissues using TCGA and Cancer Cell Line datasets. Heat maps of peptide encoding lncRNA transcripts comparing expression in tumour versus normal tissues using TCGA and Cancer Cell Line datasets. Blue heatmap – Expression presented as a mean of all samples [log2(fpkm+0.001)] dependent on anatomical site of the tumour corresponding to normal tissue; red/green heatmap – representation of tumour/normal ratio [Log2(Tumour/Normal FPKM ratio)], red represents higher expression in normal when green represents the higher expression in tumour tissue; orange heatmap – expression level in different colorectal cancer cell lines [Log2(fpkm+0.001)]; light blue – Row Z-score normalised expression level in microsatellite stable vs instable patients.

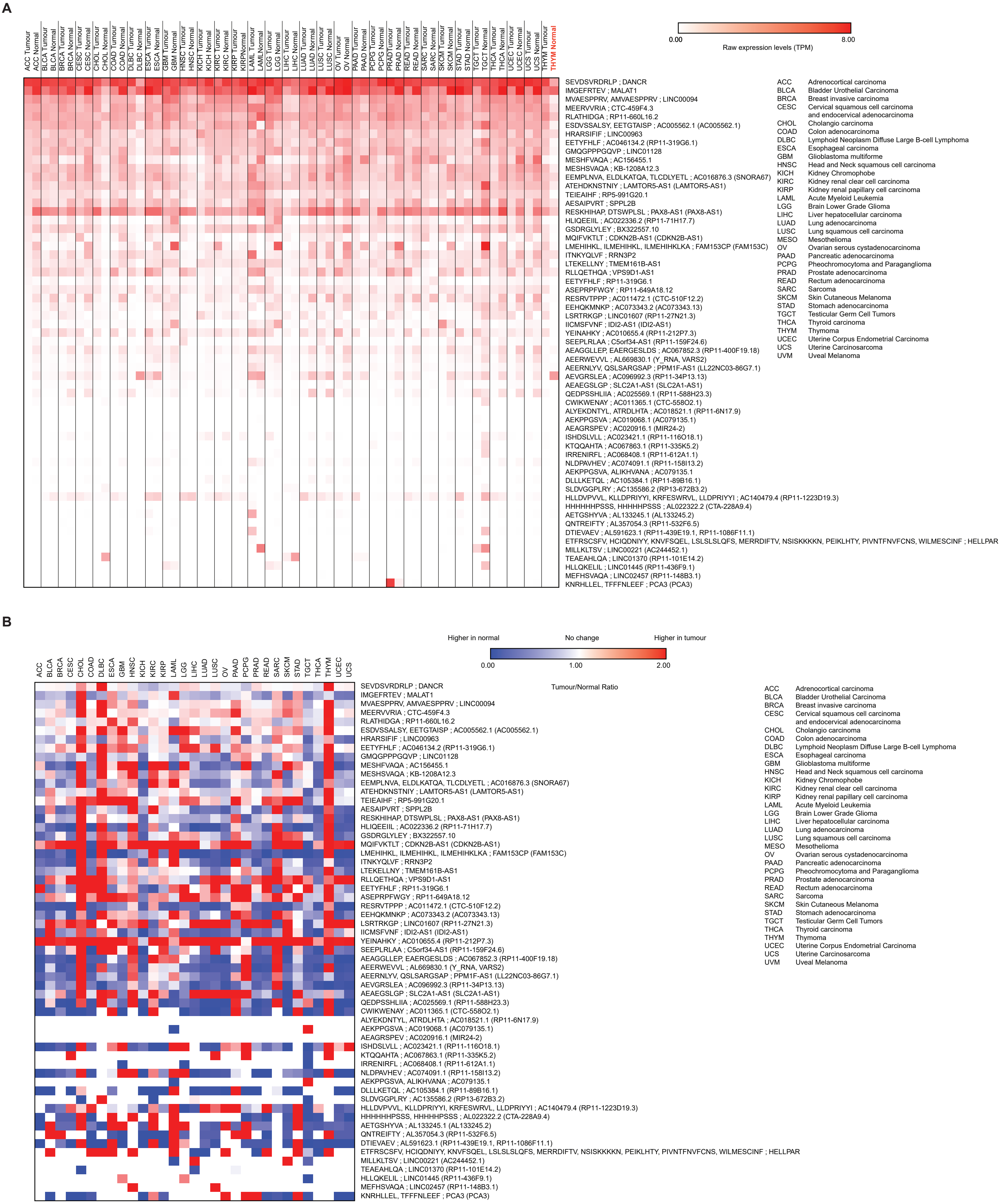
A
ACC Tumour
ACC Normal
BLCA Tumour
BLCA Normal
BRCA Tumour
BRCA Normal
CESC Tumour
CESC Normal
CHOL Tumour
CHOL Normal
COAD Tumour
COAD Normal
DLBC Tumour
DLBC Normal
ESCA Tumour
ESCA Normal
GBM Tumour
GBM Normal
HNSC Tumour
HNSC Normal
KICH Tumour
KICH Normal
KIRC Tumour
KIRC Normal
KIRP Tumour
KIRPNormal
LAML Tumour
LAMLNormal
LGG Tumour
LGG Normal
LIHC Tumour
LIHC Normal
LUAD Tumour
LUAD Normal
LUSC Tumour
LUSC Normal
OV Tumour
OV Normal
PAAD Tumour
PAAD Normal
PCPG Tumour
PCPG Normal
PRAD Tumour
PRAD Normal
READ Tumour
READ Normal
SARC Tumour
SARC Normal
SKCM Tumour
SKCM Normal
STAD Tumour
STAD Normal
TGCT Tumour
TGCT Normal
THCA Tumour
THCA Normal
UCEC Tumour
UCEC Normal
UCS Tumour
UCS Normal
THYM Tumour
THYM Normal
0.00
8.00
Raw expression levels (TPM)
SEVDSVRDRLP ; DANCR
IMGEFRTEV ; MALAT1
MVAESPPRV, AMVAESPPRV ; LINC00094
MEERVVRIA ; CTC-459F4.3
RLATHIDGA ; RP11-660L16.2
ESDVSSALSY, EETGTAISP ; AC005562.1 (AC005562.1)
HRARSIFIF ; LINC00963
EETYFHLF ; AC046134.2 (RP11-319G6.1)
GMQGPPPGQVP ; LINC01128
MESHFVAQA ; AC156455.1
MESHSVAQA ; KB-1208A12.3
EEMPLNVA, ELDLKATQA, TLCDLYETL ; AC016876.3 (SNORA67)
ATEHDKNSTNIY ; LAMTOR5-AS1 (LAMTOR5-AS1)
TEIEAIHF ; RP5-991G20.1
AESAIPVRT ; SPPL2B
RESKHIHAP, DTSWPLSL ; PAX8-AS1 (PAX8-AS1)
HLIQEEIIL ; AC022336.2 (RP11-71H17.7)
GSDRGLYLEY ; BX322557.10
MQIFVKTLT ; CDKN2B-AS1 (CDKN2B-AS1)
LMEHIHKL, ILMEHIHKL, ILMEHIHKLKA ; FAM153CP (FAM153C)
ITNKYQLVF ; RRN3P2
LTEKELLNY ; TMEM161B-AS1
RLLQETHQA ; VPS9D1-AS1
EETYFHLF ; RP11-319G6.1
ASEPRPFWGY ; RP11-649A18.12
RESRVTPPP ; AC011472.1 (CTC-510F12.2)
EEHQKMNKP ; AC073343.2 (AC073343.13)
LSRTRKGP ; LINC01607 (RP11-27N21.3)
IICMSFVNF ; IDI2-AS1 (IDI2-AS1)
YEINAHKY ; AC010655.4 (RP11-212P7.3)
SEEPLRLAA ; C5orf34-AS1 (RP11-159F24.6)
AEAGGLLEP, EAERGESLDS ; AC067852.3 (RP11-400F19.18)
AEERWEVVL ; AL669830.1 (Y_RNA, VARS2)
AEERNLYV, QSLSARGSAP ; PPM1F-AS1 (LL22NC03-86G7.1)
AEVGRSLEA ; AC096992.3 (RP11-34P13.13)
AEAEGSLGP ; SLC2A1-AS1 (SLC2A1-AS1)
QEDPSSHLIIA ; AC025569.1 (RP11-588H23.3)
CWIKWENAY ; AC011365.1 (CTC-558O2.1)
ALYEKDNTYL, ATRDLHTA ; AC018521.1 (RP11-6N17.9)
AEKPPGSVA ; AC019068.1 (AC079135.1)
AEAGRSPEV ; AC020916.1 (MIR24-2)
ISHDSLVLL ; AC023421.1 (RP11-116O18.1)
KTQQAHTA ; AC067863.1 (RP11-335K5.2)
IRRENIRFL ; AC068408.1 (RP11-612A1.1)
NLDPAVHEV ; AC074091.1 (RP11-158I13.2)
AEKPPGSVA, ALIKHVANA ; AC079135.1
DLLLKETQL ; AC105384.1 (RP11-89B16.1)
SLDVGGPLRY ; AC135586.2 (RP13-672B3.2)
HLLDVPVVL, KLLDPRIYYI, KRFESWRVL, LLDPRIYYI ; AC140479.4 (RP11-1223D19.3)
HHHHHHPSSS, HHHHHPSSS ; AL022322.2 (CTA-228A9.4)
AETGSHYVA ; AL133245.1 (AL133245.2)
QNTREIFTY ; AL357054.3 (RP11-532F6.5)
DTIEVAEV ; AL591623.1 (RP11-439E19.1, RP11-1086F11.1)
ETFRSCSFV, HCIQDNIYY, KNVFSQEL, LSLSLSLQFS, MERRDIFTV, NSISKKKKN, PEIKLHTY, PIVTNFVNFCNS, WILMESCINF ; HELLPAR
MILLKLTSV ; LINC00221 (AC244452.1)
TEAEAHLQA ; LINC01370 (RP11-101E14.2)
HLLQKELIL ; LINC01445 (RP11-436F9.1)
MEFHSVAQA ; LINC02457 (RP11-148B3.1)
KNRHLLEL, TFFFNLEEF ; PCA3 (PCA3)
ACC Adrenocortical carcinoma
BLCA Bladder Urothelial Carcinoma
BRCA Breast invasive carcinoma
CESC Cervical squamous cell carcinoma and endocervical adenocarcinoma
CHOL Cholangio carcinoma
COAD Colon adenocarcinoma
DLBC Lymphoid Neoplasm Diffuse Large B-cell Lymphoma
ESCA Esophageal carcinoma
GBM Glioblastoma multiforme
HNSC Head and Neck squamous cell carcinoma
KICH Kidney Chromophobe
KIRC Kidney renal clear cell carcinoma
KIRP Kidney renal papillary cell carcinoma
LAML Acute Myeloid Leukemia
LGG Brain Lower Grade Glioma
LIHC Liver hepatocellular carcinoma
LUAD Lung adenocarcinoma
LUSC Lung squamous cell carcinoma
MESO Mesothelioma
OV Ovarian serous cystadenocarcinoma
PAAD Pancreatic adenocarcinoma
PCPG Pheochromocytoma and Paraganglioma
PRAD Prostate adenocarcinoma
READ Rectum adenocarcinoma
SARC Sarcoma
SKCM Skin Cutaneous Melanoma
STAD Stomach adenocarcinoma
TGCT Testicular Germ Cell Tumors
THCA Thyroid carcinoma
THYM Thymoma
UCEC Uterine Corpus Endometrial Carcinoma
UCS Uterine Carcinosarcoma
UVM Uveal Melanoma
B
Higher in normal
No change
Higher in tumour
0.00
1.00
2.00
Tumour/Normal Ratio
ACC
BLCA
BRCA
CESC
CHOL
COAD
DLBC
ESCA
GBM
HNSC
KICH
KIRC
KIRP
LAML
LGG
LIHC
LUAD
LUSC
OV
PAAD
PCPG
PRAD
READ
SARC
SKCM
STAD
TGCT
THCA
THYM
UCEC
UCS
SEVDSVRDRLP ; DANCR
IMGEFRTEV ; MALAT1
MVAESPPRV, AMVAESPPRV ; LINC00094
MEERVVRIA ; CTC-459F4.3
RLATHIDGA ; RP11-660L16.2
ESDVSSALSY, EETGTAISP ; AC005562.1 (AC005562.1)
HRARSIFIF ; LINC00963
EETYFHLF ; AC046134.2 (RP11-319G6.1)
GMQGPPPGQVP ; LINC01128
MESHFVAQA ; AC156455.1
MESHSVAQA ; KB-1208A12.3
EEMPLNVA, ELDLKATQA, TLCDLYETL ; AC016876.3 (SNORA67)
ATEHDKNSTNIY ; LAMTOR5-AS1 (LAMTOR5-AS1)
TEIEAIHF ; RP5-991G20.1
AESAIPVRT ; SPPL2B
RESKHIHAP, DTSWPLSL ; PAX8-AS1 (PAX8-AS1)
HLIQEEIIL ; AC022336.2 (RP11-71H17.7)
GSDRGLYLEY ; BX322557.10
MQIFVKTLT ; CDKN2B-AS1 (CDKN2B-AS1)
LMEHIHKL, ILMEHIHKL, ILMEHIHKLKA ; FAM153CP (FAM153C)
ITNKYQLVF ; RRN3P2
LTEKELLNY ; TMEM161B-AS1
RLLQETHQA ; VPS9D1-AS1
EETYFHLF ; RP11-319G6.1
ASEPRPFWGY ; RP11-649A18.12
RESRVTPPP ; AC011472.1 (CTC-510F12.2)
EEHQKMNKP ; AC073343.2 (AC073343.13)
LSRTRKGP ; LINC01607 (RP11-27N21.3)
IICMSFVNF ; IDI2-AS1 (IDI2-AS1)
YEINAHKY ; AC010655.4 (RP11-212P7.3)
SEEPLRLAA ; C5orf34-AS1 (RP11-159F24.6)
AEAGGLLEP, EAERGESLDS ; AC067852.3 (RP11-400F19.18)
AEERWEVVL ; AL669830.1 (Y_RNA, VARS2)
AEERNLYV, QSLSARGSAP ; PPM1F-AS1 (LL22NC03-86G7.1)
AEVGRSLEA ; AC096992.3 (RP11-34P13.13)
AEAEGSLGP ; SLC2A1-AS1 (SLC2A1-AS1)
QEDPSSHLIIA ; AC025569.1 (RP11-588H23.3)
CWIKWENAY ; AC011365.1 (CTC-558O2.1)
ALYEKDNTYL, ATRDLHTA ; AC018521.1 (RP11-6N17.9)
AEKPPGSVA ; AC019068.1 (AC079135.1)
AEAGRSPEV ; AC020916.1 (MIR24-2)
ISHDSLVLL ; AC023421.1 (RP11-116O18.1)
KTQQAHTA ; AC067863.1 (RP11-335K5.2)
IRRENIRFL ; AC068408.1 (RP11-612A1.1)
NLDPAVHEV ; AC074091.1 (RP11-158I13.2)
AEKPPGSVA, ALIKHVANA ; AC079135.1
DLLLKETQL ; AC105384.1 (RP11-89B16.1)
SLDVGGPLRY ; AC135586.2 (RP13-672B3.2)
HLLDVPVVL, KLLDPRIYYI, KRFESWRVL, LLDPRIYYI ; AC140479.4 (RP11-1223D19.3)
HHHHHHPSSS, HHHHHPSSS ; AL022322.2 (CTA-228A9.4)
AETGSHYVA ; AL133245.1 (AL133245.2)
QNTREIFTY ; AL357054.3 (RP11-532F6.5)
DTIEVAEV ; AL591623.1 (RP11-439E19.1, RP11-1086F11.1)
ETFRSCSFV, HCIQDNIYY, KNVFSQEL, LSLSLSLQFS, MERRDIFTV, NSISKKKKN, PEIKLHTY, PIVTNFVNFCNS, WILMESCINF ; HELLPAR
MILLKLTSV ; LINC00221 (AC244452.1)
TEAEAHLQA ; LINC01370 (RP11-101E14.2)
HLLQKELIL ; LINC01445 (RP11-436F9.1)
MEFHSVAQA ; LINC02457 (RP11-148B3.1)
KNRHLLEL, TFFFNLEEF ; PCA3 (PCA3)
ACC Adrenocortical carcinoma
BLCA Bladder Urothelial Carcinoma
BRCA Breast invasive carcinoma
CESC Cervical squamous cell carcinoma and endocervical adenocarcinoma
CHOL Cholangio carcinoma
COAD Colon adenocarcinoma
DLBC Lymphoid Neoplasm Diffuse Large B-cell Lymphoma
ESCA Esophageal carcinoma
GBM Glioblastoma multiforme
HNSC Head and Neck squamous cell carcinoma
KICH Kidney Chromophobe
KIRC Kidney renal clear cell carcinoma
KIRP Kidney renal papillary cell carcinoma
LAML Acute Myeloid Leukemia
LGG Brain Lower Grade Glioma
LIHC Liver hepatocellular carcinoma
LUAD Lung adenocarcinoma
LUSC Lung squamous cell carcinoma
MESO Mesothelioma
OV Ovarian serous cystadenocarcinoma
PAAD Pancreatic adenocarcinoma
PCPG Pheochromocytoma and Paraganglioma
PRAD Prostate adenocarcinoma
READ Rectum adenocarcinoma
SARC Sarcoma
SKCM Skin Cutaneous Melanoma
STAD Stomach adenocarcinoma
TGCT Testicular Germ Cell Tumors
THCA Thyroid carcinoma
THYM Thymoma
UCEC Uterine Corpus Endometrial Carcinoma
UCS Uterine Carcinosarcoma
UVM Uveal Melanoma

Supplementary Figure 10. Expression of human lncRNAs giving rise to peptides in normal and tumour tissue using TCGA database. **(A)** LncRNA expression is presented as the mean of all samples [log2(fpkm)] dependent on anatomical site of the tumour and normal tissue; **(B)** representation of lncRNA expression tumour/normal ratio [Log2(Tumour/Normal FPKM ratio)], red colouring represents higher expression in tumour and blue colour represents higher expression in normal tissue. Ivory colour represents no change.

Fig 1C uncropped blots

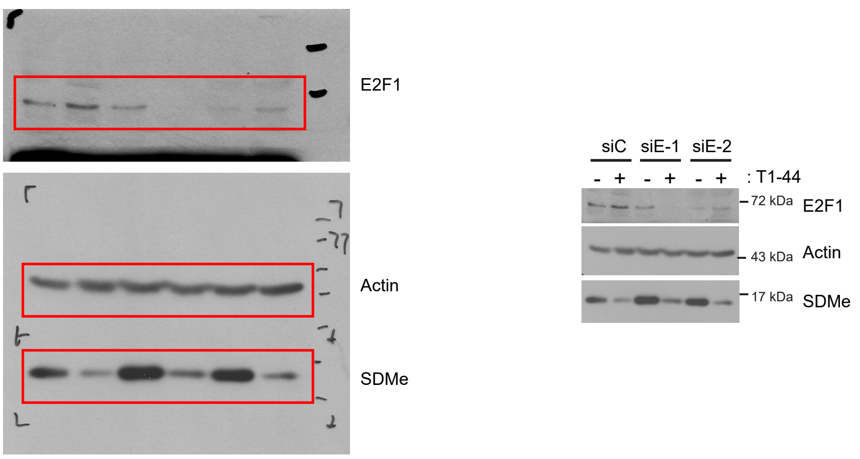

Fig 3C uncropped blots

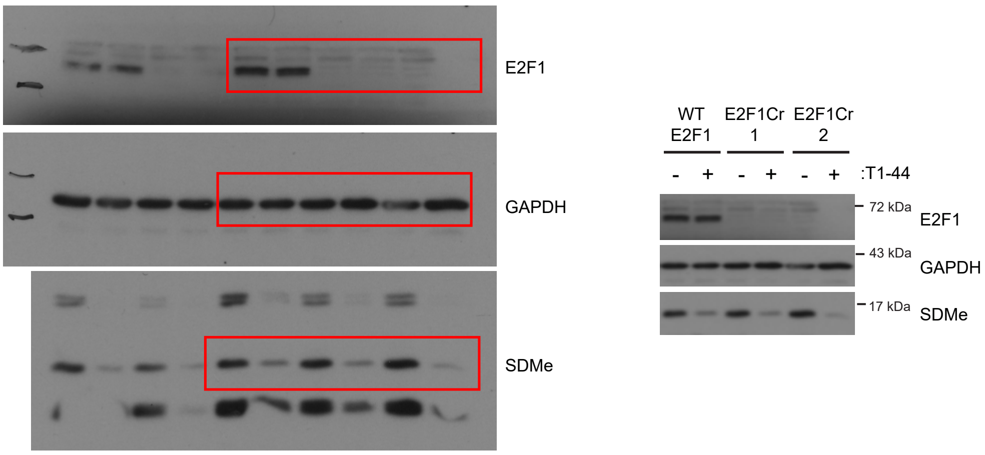

Fig 4G uncropped blots

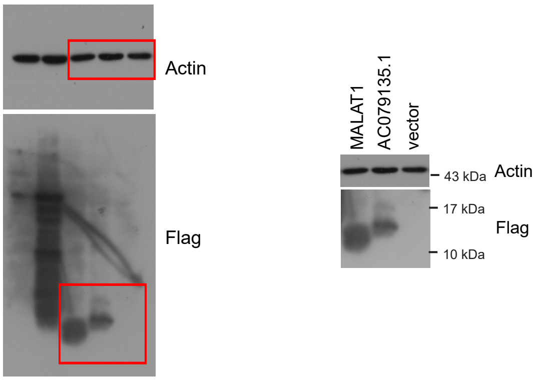

Figure S2C - uncropped blots

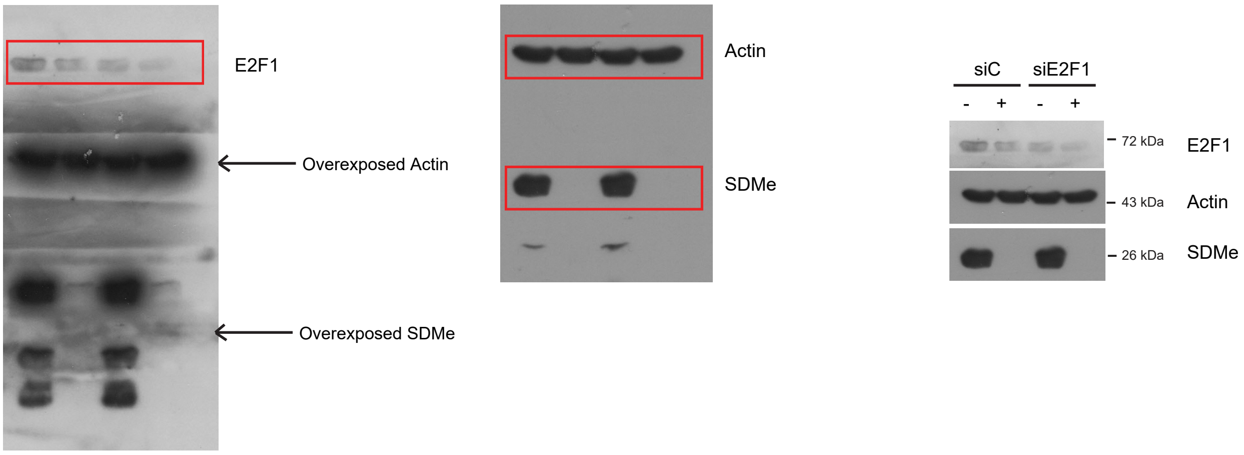

Fig S3D uncropped blots

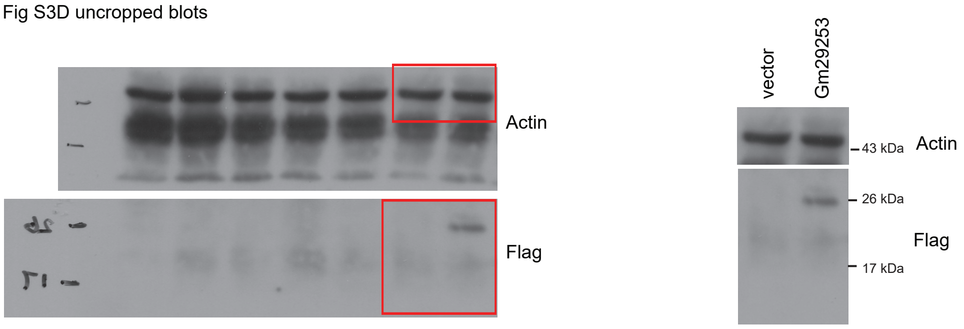

Figure S4C - uncropped blots

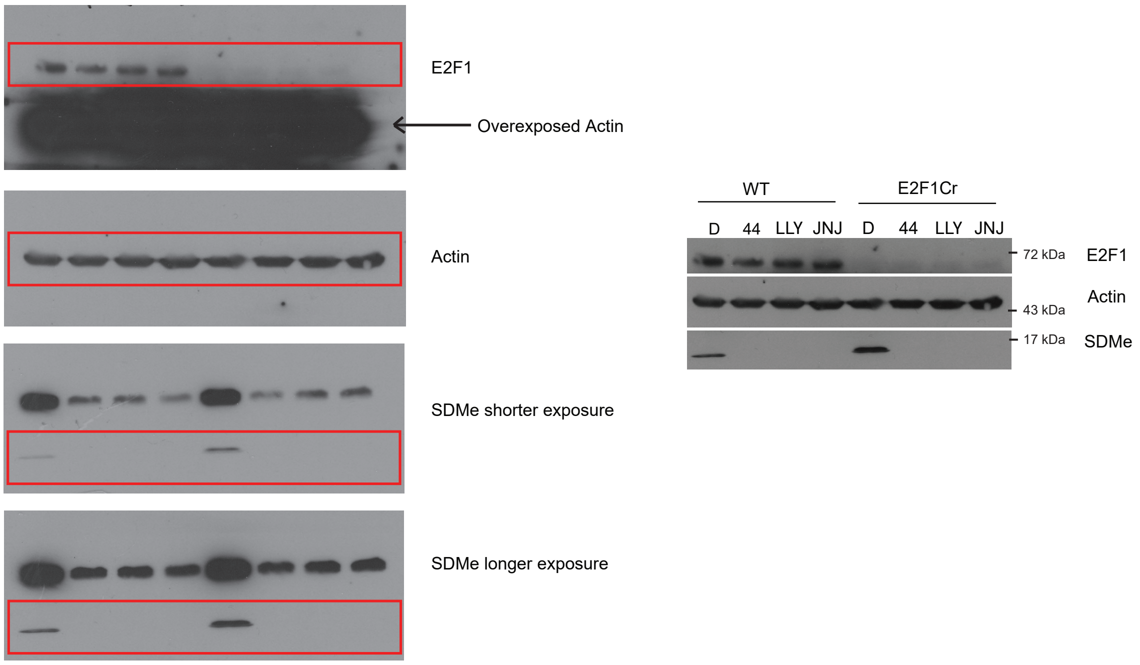

Figure S4D - uncropped blots

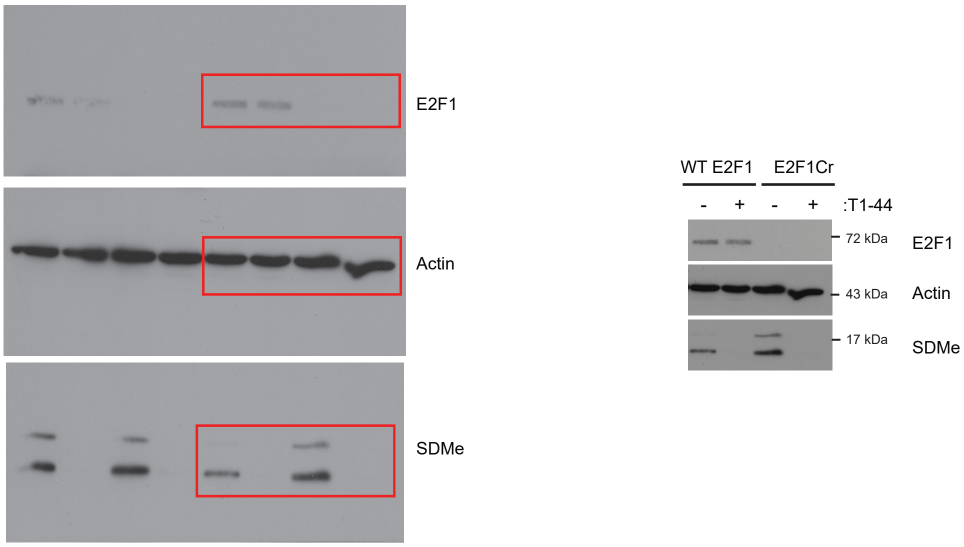

Figure S7A - uncropped blots

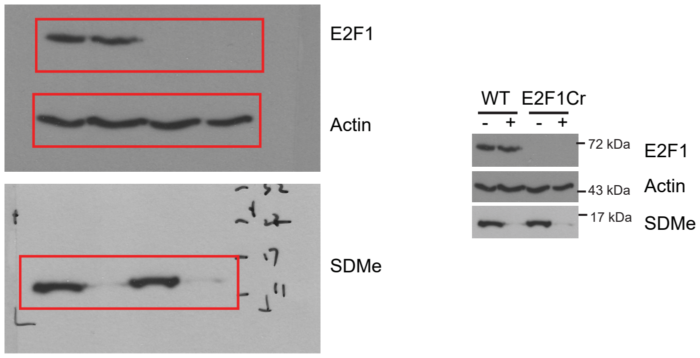

Figure S7B - uncropped blots

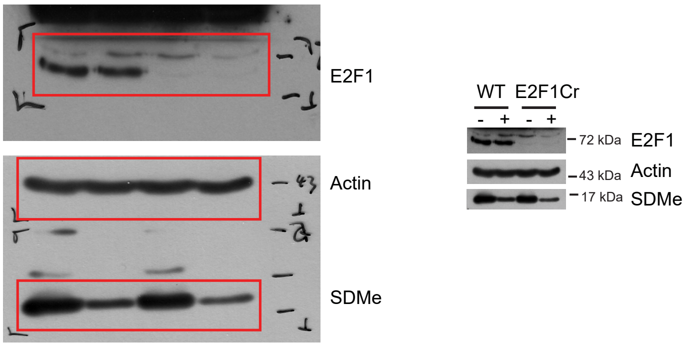

Supplementary Figure 11. Uncropped versions of immunoblots used in Figures 1C, 3C, 4G, S2C, S3D, S4C, S4D, S7A and S7B.

Supplementary Table 1. Primer list for mRNA expression (RT-PCR) experiments.

Target	Forward	Reverse
4930473A02Rik-202	CACTGCGACAGGAAAGCCAT,	AGGTGCCATAATTTTAGTGTCCTGA
Gm45441-201	TCCTGGCTCAGCCCCTG,	CCCACCGCATGGATTTCCTA
Gm15156-201	TAAGATCAGGCTGTGCTGGG,	CAGCTTCAGCCGCTACCAG
Lncppara-201	CTATGGCTTCCCTGTGGAGG,	AAGGCCGTCAGTCAGTCTTG
Kcnmb4os1-201	GCTCTCGAATCAATCATTACTGAGG,	TTCACTGGCTGGGTTGTTCC
Lncenc1-201	GACCTGCTCTAAGCCTGACC,	GACGCTGGCCTGGTTATAAC
Epb41l4aos-202	GTGGGATCTAGGGTGTCTGCAG,	CAAGGACACCCGTGTGACC
Gm36445-205	GTGATGTCAGTATCATGCTTTGTG,	CCAATTGTAGCCTATAGCAATGAG
G630030J09Rik-201	CAGCTGTCAAGACAAAGCCAC,	CAAGCTGGTGCATGTAGGAG
Ptprv-210	GAAGAGCAGCCTACAACAGC,	CTTCAGAATCGTCTCAGTGGAG
Gm44148-201	CTTTGAAGGTGCACTGAACG,	GAGTTGCCTGGCTGTTCTTAC
Gm46565-202	GGTCTCTAGCTGTGATGCTGAC,	GAAGCGTCTTGCTAACAGCTC
Gm37283	CACATTCCTGAGGGCAGGTT,	TCAGCGACTGTGTGTGAGTT
4933406J09Rik	GGATCTAGCTGTGCATCCCA,	AGTCAGGTGCAGCAACAAGT
Gm20621	TTTGTGGAGCCTCACCCTCT,	GTCACCGGTAACGGGCTTAT
Etos1	CATCGAAGCTTGCTGGCAC,	AACCTGTAGGCCCATTGTGG
Gm29253	AGGTTAGAGACCAGGGAGGC,	CCTCCACAGAGTAGTGCGTC
Gm17173	CTCTCCAGTGTTGGGCAAGT,	CACTTCTGAAAGTGTGGCCTG
Gm37494	TCAAATACCTGAAAGGGCTCG,	ACTGAATATTGCACCACATTGC
Gm15635	TGGAGGCCTATGAATCTGAGGG,	GATGTCTTCCGTGTGCCTGAG
E330035G20Rik	CCCGTCCGTTAGCTTCTCAG,	ACAAAAACCCAGTTTGCACCA
Gm47341	GCCGGCCCTTGTCTCTATC,	GGCAGACTCATCTCGTGCTT
Gm20939	TGGAAGGCATGAAAGAAGTCG,R:	ACAGTGATGTACAAAGGCTTCA
Gm35867	CAACTGGAGTCTCAAGGGCCA,	AGCACTGGTGATCCAGAGAACA
Platr1	AGTGCTGGGATCAAAGGAATTT,	CTTCCAAAGAAAACTTCATGGGG
Gm37855	GGAAAACTGAAAGGTCCTGAGC,	AGAACGGCCAGTGTCAGAGA
1110038B12Rik	GGTGATTCTGAGTGTCTCGCT,	CCACTGCCGATTTTGGACCC
EU599041	CCTCGCGACATGCCTTCTAT,	ATGCAGGAAGGTCTTACAGGC
Gm45025	GTAAGTGCCATCTTCTCCAGG,	GAGTTTGTCTGACGGTAAAAGC
9630015K15Rik	TGTTCTGGCTATACAGGGGC,	TGAGGCCCACATAGACAGGT
Gm16538	TAGCCTATCGGCATCTTCAGC,	CTCTCCGGCTCACCTATGTC
Gm47761	CCACTGCTCGCCAGAATGTT,	TCATTCGGGCGTTGGTCAT
4732463B04Rik	CCCAATGTCTGGGCCTTTTAC,	ACCCCAAGGTTTAGCATGGTC
Gm48957	GGAGCCATGGAATGGATCGT,	AGTGTTTAGCCAGGAAGTCTCC
Gm42047	GTTACACCCCAACAGCCAG,	GGCGAGTTCTTGAATCCCTG
Gm32736	GTTGCTAAAGACGCTGAACCTG,	GGCTAGAAAGGGCTCAAGGG
Gm48381	TCTTGGCATTTAGGAGGCCAG,	CCCATGCCGCTTTTGTTGAG
AC004943.2	CCGAAAAGAAAGCTTACAATCTG,	CCAACAGACGACTATTCGGAG
AC006504(CTC-459F4.3)	GGAGCAGTAGCATCTTAGCTG,	CCTCCCTCAACGTATCCATAC

AC018445.6	CCTTGACTGAAGACTCGGGG,	AACAGAGGCTGGAGGTCTCA
AC079135.1	GATGAAAGCTTTATGATGTTTGC,	CAGTTAGCAAAGAGGAACCG
AP003352	GACCACATACTTTATATTTCCATGAC,	CAGGAGAATCACTTGAACTCG
BX322557.10	CGTTTCTGGTCTTTGAAAGAC,	GCTGGCTACCATCTTTATGTC
C5orf34-AS1	GATGACATAGCTGGACTGTACTGC,	CGAAACCCCTTCTCCACC
DANCR	CTTCATGTTCACCTTTTCAACC,	CAGAGTATTCAGGGTAAGGGTC
HELLPAR	TGCTGAAAATGGTATGTCCCCA,	CCCAAGCCCATCTGGCAATA
MALAT1	GTCGGCAATATGTTGTTTTTC,	CCTGAAAAAGAGAAACCTACAAC
PPM1F-AS1	GGGCCACCTCAGAAGAAAC,	GGCAGGAGTTCAAGACCAG
RP11-319G6.1	GGACTCAGGACATAGACTCGAG,	GGTTCTGAAGTCCCTAGATGG
RP11-649A18	GTGTCCCTCTGCTCTGGTAAC,	CTCAGTGCAGAGCATGCTG
RP11-660L16.2	GATCATCGTGCCTCAGTTTC,	CCTAGGACCAAGAACTGTGTC
SLC2A1-AS1	ACAATTTGGGAACCCTCAAAG,	GCCCTGCAGATATTCTTTACCTC
SPPL2B	CCAGTGTTGCACCCTGAG,	GAGGGCCTCTCTCAACTACG
VPS9D1-AS1	CAAGCCATGGGTAACCAG,	CTAGCACAGCAGTGTCTGGAG
TTC28-AS1-214	GTGTGACATTTTCTGACTATGGAAATGATA	GCAAAACTTCTTGAATCACAGCTGC
RNASEH1-AS1-202	TACATTGGCGTGGGTCCATT	TGTATCATACAGCACATCTCAATAGCCA
LINC01128-228	CTATGTAGAAGCGGAATCTCACCAGT	CCTCACACACCTTCCAGGTCA
CCNT2-AS1-203	CGGAAGGAAAAGATCACTCACTCTTG	CATGAGTGGCATGTCAGTGCTT
UBL7-AS1-207	GCAATTGAGCAGGAATGTCACATAA	CGAACATCAGAAGGAACAAACTCC
LNCOC1-206	AGCCAAGGAAACCTGGTCTTTC	TGGCGGTGGAGAAGTCAAA
CERNA1-203	CTTGGCCGCAGAGAATGAGA	GCAAGTGGAGGCTGGCTTC
ZFAS1-201	ATACATATAAAATTGAAACTGGCGATGGAA	TCAAAGTCTAACAATGACATTCTTGAAGTG
LINC00963-262	AATCTTCCAGAGAGAAGCAAGGTCTC	AATGACTCAGGCTGGGCTCTGT

Supplementary Table S2. Primer list for ChIP experiments and lncRNA ORF cloning.

**ChIP primers**		
**Target**	**Forward**	**Reverse**
AC079135.1	GGCCTCATCCTCCAGCTC,	GGCTTCGCTCGGTGAGTC;
SPPL2B	GCTCACCGCCATCTTGTC,	GATGTTTCCCAGCAACGC
RP11-660L16.2	GCCGGACTCGAGATTGAC,	GCTGGGATCCCGAAGAAG
ASB16-AS1	GACGGGCTGACGTAAAAGG,	CACCCTGGATTGCCTAAGG
MALAT1	CCGAAGAACTACTTTTTGCCTC,	CTTATCTGCGGTTTCCTCAAG
DANCR	GACACCGACAGCCAATGG,	CAATCCCGGGAAGACTCTG
AC004943.2	GTCTAAGGCGATAGGCGTTGCT	CCTAGTCTCTCTAGCAGGGAGTTTTCC
UBL7-AS1	GCGTTCCAACCTGGCAGA	TGGGTGCTTTGGTTGGAGAG
CERNA1	TGATGGAGAAAGCCAGACGG	GAACGGATCGCGTTCTTGC
CCNT2-AS1	ACAGCCATGGAGCGTGACTT	CAGTCTCGTAGGCGTGCGA
LINC00963	GTGGGGACATTTTTCGTGG	CAGATGACATCAGCCGGC
RNASEH1-AS1	TGTCGGTACTTGAAGAAGCGG	GGAGAGAAGGGGCCCAAC
TTC28-AS1	CCTAGCTCCGCCCAGTTTC	CTCGTAAGCAGACAAGAGTGCG
ZFAS1-AS1	GCACTTTCGGTTTCCGTTC	CTCGTGCTCTCCACCCTG
CDC6	GGCCTCACAGCGACTGTAAGA	CTCGGACTCACCACAAGC
ACTIN	CCCTCCTCCTCTTCCTCAATCTC	AGCCATAAAAGGCAACTTTCGG
**LncRNA ORF cloning primers**		
**Target**	**Forward**	**Reverse**
MALAT1	ACTGACGAATTCGGCGTTGTGCGTAGA	ACTGACCTCGAGCACCTCAGTACGAAA
AC079135.1	ATATATGAGCTCGACGCGGAGAAGCCG	ACTGACGATATCAGAAGTGATTCCATT
Gm29253	ATTAATGAATTCGCGGCCCCTCAAGGC	ACCGGCCTCGAGTCAATTGTGCACTTG
