# Supplementary material for: Long non-coding RNA-derived peptides are immunogenic and drive a potent anti-tumour response
Source: Nat Commun. 2023 Feb 25;14:1078. doi: 10.1038/s41467-023-36826-0 (PMC9968330; doi:10.1038/s41467-023-36826-0)
Supplement: Supplementary file 2 — Reporting Summary [file 41467_2023_36826_MOESM2_ESM.pdf]

nature portfolio

Corresponding author(s): Nicholas B La Thangue

Last updated by author(s): Jan 30, 2023

Reporting Summary

Nature Portfolio wishes to improve the reproducibility of the work that we publish. This form provides structure for consistency and transparency in reporting. For further information on Nature Portfolio policies, see our Editorial Policies and the Editorial Policy Checklist.

Statistics

For all statistical analyses, confirm that the following items are present in the figure legend, table legend, main text, or Methods section.

n/a	Confirmed	
☐	☒	The exact sample size (*n*) for each experimental group/condition, given as a discrete number and unit of measurement
☐	☒	A statement on whether measurements were taken from distinct samples or whether the same sample was measured repeatedly
☐	☒	The statistical test(s) used AND whether they are one- or two-sided<br>*Only common tests should be described solely by name; describe more complex techniques in the Methods section.*
☒	☐	A description of all covariates tested
☒	☐	A description of any assumptions or corrections, such as tests of normality and adjustment for multiple comparisons
☐	☒	A full description of the statistical parameters including central tendency (e.g. means) or other basic estimates (e.g. regression coefficient) AND variation (e.g. standard deviation) or associated estimates of uncertainty (e.g. confidence intervals)
☐	☒	For null hypothesis testing, the test statistic (e.g. *F*, *t*, *r*) with confidence intervals, effect sizes, degrees of freedom and *P* value noted<br>*Give P values as exact values whenever suitable.*
☒	☐	For Bayesian analysis, information on the choice of priors and Markov chain Monte Carlo settings
☒	☐	For hierarchical and complex designs, identification of the appropriate level for tests and full reporting of outcomes
☒	☐	Estimates of effect sizes (e.g. Cohen's *d*, Pearson's *r*), indicating how they were calculated

Our web collection on statistics for biologists contains articles on many of the points above.

Software and code

Policy information about availability of computer code

Data collection	Data was collected using commercially available software (AriaMX real-time qPCR instrument (AriaMX v1.8 software, Agilent), Synergy 2 microplate reader (Biotek), Illumina HiSeq Platform (Illumina), Orbitrap Fusion Lumos Tribrid (Thermo Scientific) or Q Exactive HF-X (Thermo Scientific) mass spectrometers (Peaks v8.5 software, Bioinformatics Solutions), ELIspot counter (AID ELIspot software v7, Autoimmun Diagnostika), Agilent 2100 Bioanalyzer (Agilent), Ultimate 3000 HPLC system (ThermoFisher Scientific), nano EASY-Spray source at 2000 V (Thermo Scientific), Nanodrop (Thermo Fisher Scientific), MoFlo cell sorter (Beckman Coulter)
Data analysis	Data was analysed using commercial softwares (AriaMX v1.8 software (Agilent), TrimGalore v.0.4.3 (Babraham Bioinformatics), Kallisto v.0.44.0 (Pachter Lab), Sleuth package v.0.30.0 (Pachter Lab), Peaks v8.5 software (Bioinformatics Solutions), Progenesis QI v2.0 for proteomics software (Waters), NetMHC4.0 software (DTU Health Tech), ImageJ software v1 Fiji package (NIH), Graphpad prism v8 software (Dotmatics), Xena Browser v1 (University of California), Heatmapper tool v1 (University of Alberta), DESeq2 R Bioconductor package (v.1.25.17) (Bioconductor), GEPIA v2 (http://gepia2.cancer-pku.cn/), Broad Institute Cancer Cell Line Encyclopedia v1 (portals.broadinstitute.org › ccle), Genevestigator tool v9.7.0 (Nebion AG), Morpheus software v1 (Broad Institute; https:// software.broadinstitute.org/morpheus/)

For manuscripts utilizing custom algorithms or software that are central to the research but not yet described in published literature, software must be made available to editors and reviewers. We strongly encourage code deposition in a community repository (e.g. GitHub). See the Nature Portfolio guidelines for submitting code & software for further information.

Data

Policy information about availability of data

All manuscripts must include a data availability statement. This statement should provide the following information, where applicable:

- Accession codes, unique identifiers, or web links for publicly available datasets
- A description of any restrictions on data availability
- For clinical datasets or third party data, please ensure that the statement adheres to our policy

Source data are provided with this paper. Additional data and materials are available from the corresponding author upon reasonable request. The RNA-seq data have been deposited in the Gene Expression Omnibus (GEO) under accession codes GSE142430 [https://www.ncbi.nlm.nih.gov/geo/query/acc.cgi?acc=GSE142430] and GSE181401 [https://www.ncbi.nlm.nih.gov/geo/query/acc.cgi?acc=GSE181401]. Sequencing reads for colon26 tumour tissue experiments were aligned to the mm10 version of the mouse genome using GENCODE mouse lncRNA annotation version M22. Sequencing reads for HCT116 cells were aligned to GENCODE human lncRNA annotation version 34 and FANTOM5. The Immunopeptidomics data have been deposited in ProteomeXchange (PRIDE database) under accession codes PXD029613 [https://www.ebi.ac.uk/pride/archive/projects/PXD029613] and PXD029594 [https://www.ebi.ac.uk/pride/archive/projects/PXD029594]. All lncRNA-derived peptide sequences were reviewed with human and mouse SwissProt protein database. UCSC Genome Browser was used for human (databases: ENCODE Regulation Txn Factr ChIP E3 Track Settings, ENCODE Regulation Txn Factor ChIP Track Settings, ENC TF Binding ENCODE 3 TFBS Track Settings, ENC TF Binding Uniform TFBS Track Settings, ENC TF Binding SYDH TFBS Track Settings) and mouse lncRNA promoter analysis, (GSM288349 [https://www.ncbi.nlm.nih.gov/geo/query/acc.cgi?acc=GSM288349]). Functional genomics analysis, Xena browser (The TCGA TARGET GTEx database) and GEPIA v2 (http://gepia2.cancer-pku.cn/) were used. Broad Institute Cancer Cell Line Encyclopedia was used to analyse the expression of lncRNA genes in colorectal cancer cell lines. For the normal tissue and thymocyte expression analysis of murine lncRNAs giving rise to peptides, the Genevestigator tool was used.

Human research participants

Policy information about studies involving human research participants and Sex and Gender in Research.

Reporting on sex and gender	N/A
Population characteristics	N/A
Recruitment	N/A
Ethics oversight	N/A

Note that full information on the approval of the study protocol must also be provided in the manuscript.

Field-specific reporting

Please select the one below that is the best fit for your research. If you are not sure, read the appropriate sections before making your selection.

[x] Life sciences [ ] Behavioural & social sciences [ ] Ecological, evolutionary & environmental sciences

For a reference copy of the document with all sections, see nature.com/documents/nr-reporting-summary-flat.pdf

Life sciences study design

All studies must disclose on these points even when the disclosure is negative.

Sample size	Cells: No sample size was pre-determined. Sample sizes were chosen according to the standard generally accepted in the field (at least three biological replicates) and based on our previous experience with the HCT116 and CT26 cells (Barczak, W., Jin, L., Carr, S. M., Munro, S., Ward, S., Kanapin, A., Samsonova, A., & La Thangue, N. B. (2020). PRMT5 promotes cancer cell migration and invasion through the E2F pathway. Cell death & disease, 11(7), 572. https://doi.org/10.1038/s41419-020-02771-9; Blaszczak, W., Liu, G., Zhu, H., Barczak, W., Shrestha, A., Albayrak, G., Zheng, S., Kerr, D., Samsonova, A., & La Thangue, N. B. (2021). Immune modulation underpins the anti-cancer activity of HDAC inhibitors. Molecular oncology, 15(12), 3280–3298. https://doi.org/10.1002/1878-0261.12953). Mouse experiments: Size of the groups was determined based on power calculations to reach >80% power for the outputs required, and previous experience.
Data exclusions	One mouse from Fig. 5B experiment was excluded since it did not develop tumour. No other data were excluded.
Replication	Cells: All experiments were reproduced at least three times with similar results. All attempts at replication were successful. Mouse experiments: All experiments were performed with an appropriate number of mice as based on power calculations. All mouse tumour challenge and immunogenicity experiments (except experiment in Figure 5C - only one time) we performed twice with successful replication. In the paper we are presenting one representative experiment.
Randomization	Experiment Fig. 1D - Animals were randomly assigned to treatment or control groups based on tumour volume at the Day 0 of the treatment<br>Experiment Fig. 5B - animals were randomly assigned to treatment or control groups.<br>Experiment Fig. 5C - The animals were assigned into groups using an Excel-based randomization software performing stratified randomization based upon their tumour volumes

Experiments S8C and S8D - animals were randomly assigned to treatment or control groups.

Cells were randomly allocated to the experimental groups.

Blinding

Cells: No blinding was required, since most experiments were performed and repeated independently by at least two researchers.
Mouse experiments in Figs 1D and 5C: The scientist performing tumour volume measurements was not involved in the mouse handling and and was blinded to the experimental protocols and animal allocation. No blinding was used in experiment Fig. 5B S8C, and S8D since only one operator was available at the time to perform the experiments and all mice/cages were identified by a label and number to ensure proper treatments.

Reporting for specific materials, systems and methods

We require information from authors about some types of materials, experimental systems and methods used in many studies. Here, indicate whether each material, system or method listed is relevant to your study. If you are not sure if a list item applies to your research, read the appropriate section before selecting a response.

Materials & experimental systems

n/a	Involved in the study
☐	☒ Antibodies
☐	☒ Eukaryotic cell lines
☒	☐ Palaeontology and archaeology
☐	☒ Animals and other organisms
☒	☐ Clinical data
☒	☐ Dual use research of concern

Methods

n/a	Involved in the study
☒	☐ ChIP-seq
☒	☐ Flow cytometry
☒	☐ MRI-based neuroimaging

Antibodies

Antibodies used

B-actin (clone AC-74, Sigma-Aldrich; dilution 1:2000), E2F1 (3742S, Cell Signaling Technology, dilution 1:1000), symmetric di-methyl arginine (SDMe) (13222S, Cell Signaling Technology, dilution 1:1000), FLAG (clone M2, F1804, Sigma, 1: 1000), GAPDH (clone 6C5, MAB374, Millipore, 1:2000), W6/32 antibody (ATCC, HB-95), clone 34.1.2s (ATCC, HB-79), anti-E2F1 (A300-766A, dilution 1:1000, Bethyl Laboratories), INFγ (clone AN18, Mabtech, 3321-3-1000, dilution 1:200), biotin conjugated anti-INFγ (MabTech, 3321-6-100, clone R4-6A2-Biotin, dilution 1:2000), streptavidin-alkaline phosphatase (Mabtech, 3310-10-1000, dilution 1:750), SDMe (13222S, dilution 1:5000, Cell signaling), CD8 (ab203035, dilution 1:11000, Abcam), CD4 (ab183685, dilution 1:8000, Abcam, clone: EPR19514), CD163 (ab182422, dilution 1:5000, Abcam, clone: EPR19518).

Validation

All antibodies are commercially available and have been validated by the manufacturer (datasheets can be found on the respective suppliers websites) and in previous publications.

B-actin - was validated and use before in our previous paper (Roworth, A. P., Carr, S. M., Liu, G., Barczak, W., Miller, R. L., Munro, S., Kanapin, A., Samsonova, A., & La Thangue, N. B. (2019). Arginine methylation expands the regulatory mechanisms and extends the genomic landscape under E2F control. Science advances, 5(6), eaaw4640. https://doi.org/10.1126/sciadv.aaw4640)

E2F1 (3742S) - was validated by using siRNA and CRISPR E2F1 cells in this and our previous paper (Barczak, W., Jin, L., Carr, S. M., Munro, S., Ward, S., Kanapin, A., Samsonova, A., & La Thangue, N. B. (2020). PRMT5 promotes cancer cell migration and invasion through the E2F pathway. Cell death & disease, 11(7), 572. https://doi.org/10.1038/s41419-020-02771-9.
symmetric di-methyl arginine (SDMe) (13222S) - was validated by using PRMT5 inhibitor in this study and the previous ones (Barczak, W., Jin, L., Carr, S. M., Munro, S., Ward, S., Kanapin, A., Samsonova, A., & La Thangue, N. B. (2020). PRMT5 promotes cancer cell migration and invasion through the E2F pathway. Cell death & disease, 11(7), 572. https://doi.org/10.1038/s41419-020-02771-9)

FLAG (clone M2, F1804) was validated and use before in our previous paper (Roworth, A. P., Carr, S. M., Liu, G., Barczak, W., Miller, R. L., Munro, S., Kanapin, A., Samsonova, A., & La Thangue, N. B. (2019). Arginine methylation expands the regulatory mechanisms and extends the genomic landscape under E2F control. Science advances, 5(6), eaaw4640. https://doi.org/10.1126/sciadv.aaw4640)
GAPDH (clone 6C5, MAB374) - was validated and used in several publications including: A plastic relationship between vinculin-mediated tension and adhesion complex area defines adhesion size and lifetime. Hernández-Varas, P; Berge, U; Lock, JG; Strömblad, S Nature communications 6 7524 2015; Connexin43 Mediated Delivery of ADAMTS5 Targeting siRNAs from Mesenchymal Stem Cells to Synovial Fibroblasts. Liu, S; Niger, C; Koh, EY; Stains, JP PloS one 10 e0129999 2015)

W6/32 antibody (ATCC, HB-95) - was validated and used in several publications including Mayer, R. L., Verbeke, R., Asselman, C., Aernout, I., Gul, A., Eggermont, D., Boucher, K., Thery, F., Maia, T. M., Demol, H., Gabriels, R., Martens, L., Bécavin, C., De Smedt, S. C., Vandekerckhove, B., Lentacker, I., & Impens, F. (2022). Immunopeptidomics-based design of mRNA vaccine formulations against Listeria monocytogenes. Nature communications, 13(1), 6075. https://doi.org/10.1038/s41467-022-33721-y.

clone 34.1.2s (ATCC, HB-79) - was validated and used in several publications including Tailor, A., Estephan, H., Parker, R., Woodhouse, I., Abdulghani, M., Nicastri, A., Jones, K., Salatino, S., Muschel, R., Humphrey, T., Giaccia, A., & Ternette, N. (2022). Ionizing Radiation Drives Key Regulators of Antigen Presentation and a Global Expansion of the Immunopeptidome. Molecular & cellular proteomics :

MCP, 21(11), 100410. https://doi.org/10.1016/j.mcpro.2022.100410

anti-E2F1 (A300-766A) - was validated by using CRISP E2F1 cells in this and our previous paper (Roworth, A. P., Carr, S. M., Liu, G., Barczak, W., Miller, R. L., Munro, S., Kanapin, A., Samsonova, A., & La Thangue, N. B. (2019). Arginine methylation expands the regulatory mechanisms and extends the genomic landscape under E2F control. Science advances, 5(6), eaaw4640. https://doi.org/10.1126/sciadv.aaw4640)

INFγ (clone AN18) - was validated and used in several publications including Wen, J., Tang, W., Sheets, N. et al. Identification of Zika virus epitopes reveals immunodominant and protective roles for dengue virus cross-reactive CD8+ T cells. Nat Microbiol 2, 17036 (2017). https://doi.org/10.1038/nmicrobiol.2017.36

biotin conjugated anti-INFγ (clone R4-6A2-Biotin) - Obermajer, N., Urban, J., Wieckowski, E., Muthuswamy, R., Ravindranathan, R., Bartlett, D. L., & Kalinski, P. (2018). Promoting the accumulation of tumor-specific T cells in tumor tissues by dendritic cell vaccines and chemokine-modulating agents. Nature protocols, 13(2), 335–357. https://doi.org/10.1038/nprot.2017.130.

streptavidin-alkaline phosphatase (Mabtech, 3310-10-1000) - Angyal A, Longet S, Moore SC, et al. T-cell and antibody responses to first BNT162b2 vaccine dose in previously infected and SARS-CoV-2-naive UK health-care workers: a multicentre prospective cohort study. Lancet Microbe 2021; published online Nov 9. https://doi.org/10.1016/S2666-5247(21)00275-5

CD8 (ab203035) - was validated and used before in our previous paper (Blaszczak, W., Liu, G., Zhu, H., Barczak, W., Shrestha, A., Albayrak, G., Zheng, S., Kerr, D., Samsonova, A., & La Thangue, N. B. (2021). Immune modulation underpins the anti-cancer activity of HDAC inhibitors. Molecular oncology, 15(12), 3280–3298. https://doi.org/10.1002/1878-0261.12953)

CD4 (ab183685) - was validated and used before in our previous paper (Blaszczak, W., Liu, G., Zhu, H., Barczak, W., Shrestha, A., Albayrak, G., Zheng, S., Kerr, D., Samsonova, A., & La Thangue, N. B. (2021). Immune modulation underpins the anti-cancer activity of HDAC inhibitors. Molecular oncology, 15(12), 3280–3298. https://doi.org/10.1002/1878-0261.12953)

CD163 (ab182422) - was validated and used before in our previous paper (Blaszczak, W., Liu, G., Zhu, H., Barczak, W., Shrestha, A., Albayrak, G., Zheng, S., Kerr, D., Samsonova, A., & La Thangue, N. B. (2021). Immune modulation underpins the anti-cancer activity of HDAC inhibitors. Molecular oncology, 15(12), 3280–3298. https://doi.org/10.1002/1878-0261.12953)

Eukaryotic cell lines

Policy information about cell lines and Sex and Gender in Research

Cell line source(s)	HCT116 p53-/- cells (RRID: CVCL_HD97) and CT26 cells (ATCC, CRL-2638). All CRISPR cell lines were generated from the parental HCT116 p53-/- cells
Authentication	Authentication was certified by ATCC. CRISPR HCT116 p53-/- cell lines were confirmed by sequencing genomic regions targeted by the sgRNA used. CT26 cells were provided by ATCC which authenticates the cells by morphology, immunology or DNA fingerprint. HCT116 TP53(-/-) (RRID:CVCL_HD97) were used and authenticated previously by morphology and genotyping (Barczak, W. et al. PRMT5 promotes cancer cell migration and invasion through the E2F pathway. Cell Death 872 Dis 11, doi:10.1038/s41419-020-02771-9 (2020); Sur, S., Pagliarini, R., Bunz, F., Rago, C., Diaz, L. A., Jr, Kinzler, K. W., Vogelstein, B., & Papadopoulos, N. (2009). A panel of isogenic human cancer cells suggests a therapeutic approach for cancers with inactivated p53. Proceedings of the National Academy of Sciences of the United States of America, 106(10), 3964–3969. https://doi.org/10.1073/pnas.0813333106)
Mycoplasma contamination	All cell lines tested negative for mycoplasma contamination
Commonly misidentified lines (See ICLAC register)	No commonly misidentified cell lines were used

Animals and other research organisms

Policy information about studies involving animals; ARRIVE guidelines recommended for reporting animal research, and Sex and Gender in Research

Laboratory animals	Balb/c female mice at 6-8 weeks of age<br><br>All facilities have similar housing conditions: Temperature: 22-24ºC, 12h day/night cycle, Humidity 40-70%.
Wild animals	No wild animals were used
Reporting on sex	Female Balb/c mice were used
Field-collected samples	No field collected samples were used
Ethics oversight	Experiments performed at Charles Rivers were approved by the animal Charles River Animal Care and Use Committee at Charles River Discovery Research Services Germany (where each experiment was performed) and the National Committee for the Protection of Animals Used for Scientific Purposes for the Federal Republic of Germany, and were conducted according to all applicable international, national and local laws and guidelines. Similarly, experiments performed at WuXi AppTec were approved by WuXi

AppTec Institutional Animal Care and Use Committee.

Oxford University experiments were performed according to UK Home Office regulations and after review and approval by the The Committee on Animal Care and Ethical Review at the University of Oxford. Mice were cared for in accordance with institutional guidelines.

Note that full information on the approval of the study protocol must also be provided in the manuscript.
