# Supplementary material for: Long non-coding RNA-derived peptides are immunogenic and drive a potent anti-tumour response
Source: Nat Commun. 2023 Feb 25;14:1078. doi: 10.1038/s41467-023-36826-0 (PMC9968330; doi:10.1038/s41467-023-36826-0)
Supplement: Supplementary file 3 — Description of Additional Supplementary Files [file 41467_2023_36826_MOESM3_ESM.docx]

**Description of Additional Supplementary Files**

**Supplementary Data 1.** List of lncRNA transcripts differentially expressed in HCT116 cells.

**Supplementary Data 2.** List of lncRNA transcripts differentially expressed in CT26 cells and colon26 tumours.

**Supplementary Data 3.** List of identified lncRNA-derived peptides from the CT26 immunopeptidomics qualitative analysis.

**Supplementary Data 4.** List of identified lncRNA-derived peptides from the CT26 immunopeptidomics quantitative analysis.

**Supplementary Data 5.** List of identified lncRNA-derived peptides from the HCT116 immunopeptidomics analysis (FANTOM annotation).

**Supplementary Data 6.** List of identified lncRNA-derived peptides from the HCT116 immunopeptidomics qualitative analysis (GENCODE annotation).

**Supplementary Data 7.** List of identified lncRNA-derived peptides from the HCT116 immunopeptidomics quantitative analysis (GENCODE annotation).

**Supplementary Data 8.** In-house database of predicted murine lncRNA-derived peptides (GENCODE annotation).

**Supplementary Data 9.** In-house database of predicted human lncRNA-derived peptides (FANTOM annotation).

**Supplementary Data 10.** In-house database of predicted human lncRNA-derived peptides (GENCODE annotation).
